# Genome-wide differentially methylated genes associated with posttraumatic stress disorder and longitudinal change in methylation in rape survivors

**DOI:** 10.1038/s41398-021-01608-z

**Published:** 2021-11-19

**Authors:** Jani Nöthling, Naeemah Abrahams, Sylvanus Toikumo, Matthew Suderman, Shibe Mhlongo, Carl Lombard, Soraya Seedat, Sian Megan Joanna Hemmings

**Affiliations:** 1 Department of Psychiatry, Faculty of Medicine and Health Sciences Stellenbosch University, Cape Town, South Africa; 2 Gender and Health Research Unit, South African Medical Research Council, Cape Town, South Africa; 3 South African Medical Research Council Unit on the Genomics of Brain Disorders, Stellenbosch University, Cape Town, South Africa; 4 Division of Social and Behavioural Sciences, School of Public Health and Family Medicine, University of Cape Town, Cape Town, South Africa; 5 MRC Integrative Epidemiology Unit, Population Health Sciences, Bristol Medical School, University of Bristol, Bristol, United Kingdom; 6 Biostatistics Unit, South African Medical Research Council, Cape Town, South Africa; 7 Division of Epidemiology and Biostatistics, Department of Global Health, Stellenbosch University, Cape Town, South Africa

**Keywords:** Prognostic markers, Clinical genetics, Clinical genetics, Psychiatric disorders, Epigenetics and behaviour

## Abstract

Rape is associated with a high risk for posttraumatic stress disorder (PTSD). DNA methylation changes may confer risk or protection for PTSD following rape by regulating the expression of genes implicated in pathways affected by PTSD. We aimed to: (1) identify epigenome-wide differences in methylation profiles between rape-exposed women with and without PTSD at 3-months post-rape, in a demographically and ethnically similar group, drawn from a low-income setting; (2) validate and replicate the findings of the epigenome-wide analysis in selected genes (*BRSK2* and *ADCYAP1*); and (3) investigate baseline and longitudinal changes in *BRSK2* and *ADCYAP1* methylation over six months in relation to change in PTSD symptom scores over 6 months, in the combined discovery/validation and replication samples (*n* = 96). Rape-exposed women (*n* = 852) were recruited from rape clinics in the Rape Impact Cohort Evaluation (RICE) umbrella study. Epigenome-wide differentially methylated CpG sites between rape-exposed women with (*n* = 24) and without (*n* = 24) PTSD at 3-months post-rape were investigated using the Illumina EPIC BeadChip in a discovery cohort (*n* = 48). Validation (*n* = 47) and replication (*n* = 49) of *BRSK2* and *ADCYAP1* methylation findings were investigated using EpiTYPER technology. Longitudinal change in *BRSK2* and *ADCYAP1* was also investigated using EpiTYPER technology in the combined sample (*n* = 96). In the discovery sample, after adjustment for multiple comparisons, one differentially methylated CpG site (chr10: 61385771/ cg01700569, *p* = 0.049) and thirty-four differentially methylated regions were associated with PTSD status at 3-months post-rape. Decreased *BRSK2* and *ADCYAP1* methylation at 3-months and 6-months post-rape were associated with increased PTSD scores at the same time points, but these findings did not remain significant in adjusted models. In conclusion, decreased methylation of *BRSK2* may result in abnormal neuronal polarization, synaptic development, vesicle formation, and disrupted neurotransmission in individuals with PTSD. PTSD symptoms may also be mediated by differential methylation of the *ADCYAP1* gene which is involved in stress regulation. Replication of these findings is required to determine whether *ADCYAP1* and *BRSK2* are biomarkers of PTSD and potential therapeutic targets.

## Introduction

Rape and sexual assault are associated with a high risk for the development of posttraumatic stress disorder (PTSD) compared to other trauma types [1, 2]. Prospective studies have reported PTSD prevalence rates ranging between 35% and 45% at 3-months post-rape, with many survivors of sexual assault continuing to experience PTSD symptoms at 6-months and 12-months post-rape [3–6]. PTSD is a complex, multifactorial disorder and an array of environmental and genetic putative risk and protective factors mediate or contribute to the development of the disorder [3, 5, 7]. Epigenetic mechanisms, including DNA methylation, are known to respond to environmental exposures such as trauma, leading to stable changes in gene expression [8, 9]. DNA methylation responses may confer risk or protection for PTSD, as they may alter the ability to adapt to traumatic events on a molecular level [10]. Using a hypothesis-neutral, genome-wide approach to study epigenome-wide signatures (while accounting for potential environmental and biological confounding factors), and validating and replicating these findings, may bring us closer to uncovering the complexity of the disorder [10].

To date, twelve epigenome-wide association studies (EWASs) of blood DNA methylation differences in PTSD cases and controls have been published (see Table 1 for details). In sum, the majority of genes identified as differentially methylated in PTSD are linked to central nervous system functioning (e.g., neuron development, axonal outgrowth, synaptic connectivity, neurotransmitter release, neuroinflammation, and apoptosis) [11–17] and the immune response (T cell expression, cytokine and interferon release, phagocytosis) [13, 14, 18, 19].

**Table 1:** Summary of epigenome-wide association studies investigating posttraumatic stress disorder as the outcome.

Reference	Array and tissue type	Design and sample size	Setting and trauma type	Ethnicity	Gender and mean age	PTSD measure	PTSD associated genes/networks
Uddin et al. [18]	HM27; Blood	Cross-sectional; 23 PTSD cases 77 trauma-exposed controls	Civilians from the DNHS cohort; mixture of trauma types	79 African American, 14 Caucasian, 7 other ethnicities (not specified)	40 Male (40%) 60 (60%) Female; 45.8 years	PCL-C	Functional annotation clustering of differentially methylated genes implicated genes associated with the immune system in the development of PTSD.
Smith et al. [13]	HM27; Blood	Cross-sectional; 51 PTSD cases 53 trauma-exposed controls	Civilians from the GTP cohort; mixture of trauma types	104 African American	64 Male (61.5%) 40 Female (38.5%); 42.7 years	CAPS	Epigenome-wide significant differences in methylation at CpG sites in the *APC5*, *TLR8*, *TPR*, *CLEC9A*, *ANXA2* genes.
Mehta et al. [11]	450 K; Blood	Cross-sectional; 32 PTSD cases with CT 29 PTSD cases without CT	Civilian; mixture of trauma types	150 African Americans, 19 other ethnicities	18 Male (29.5%), 43 Female (70.5%); 41.6 years	PSS	Pathways affected by PTSD were related to apoptosis and cellular growth rate. Pathways uniquely affected in those with PTSD and CT were related to nervous system development and tolerance induction.
Chen, Kobayasji and Mellman, 2016 [19]	450 K; Blood	Cross-sectional; 12 PTSD cases 12 trauma-exposed controls	Civilian; index traumas: 8 childhood physical or sexual abuse (33.3%); 3 sexual assault (12.5%); 9 violent crime (37.5%); 2 IPV (8.3%); 2 witnessed a violent death (8.3%)	24 African American	13 Male (54.2%) 11 Female (45.8%); 22 years	CAPS	No epigenome-wide significant differences in methylation levels. Expression of genes associated with olfactory receptors, immune activation, GABAA receptor, and vitamin D synthesis was upregulated in PTSD cases.
Hammamieh et al. 2017 [12]	450 K; Blood	Cross-sectional; 79 PTSD cases 80 trauma-exposed controls	Combat exposed veterans previously deployed to Iraq or Afghanistan	159 American ethnically matched participants (not otherwise specified)	159 Males (100%); 33.9 years	CAPS	Functional enrichment analysis of differentially methylated genes implicated genes related to nervous system development/functioning, somatic complications, and endocrine signaling in the development of PTSD.
Kuan et al. 2017 [20]	450 K; Blood	Cross-sectional; 171 current PTSD cases 100 past PTSD cases 202 trauma-exposed controls	Civilian responders to the September 11^th^ World Trade Centre Disaster from the WTC cohort	382 Caucasian Americans, 91 other ethnicities (not specified)	473 Males (100%); 49.5 years	SCID	No epigenome-wide significant differences in methylation levels. Differential methylation at CpG sites in the *ZDHHC11*, *CSMD2, COL9A3, PDCD6IP, TBC1D24,* and *FAM164A* genes were associated with current PTSD at a nominal level.
Mehta et al. [14]	EPIC; Blood	Cross-sectional; 8 PTSD cases 48 trauma-exposed controls	Treatment seeking Vietnam veterans with combat exposure	96 Australian (not otherwise specified)	96 Males (100%); 68.67 years	CAPS	Epigenome-wide significant differences in methylation at CpG sites in the *BRSK1*, *NGF*, *LCN8*, *DOCK2* genes and at an intergenic site (closest gene *LRRC3B*).
Kryzewska et al. [24]	450 K, Blood	Cross-sectional;v34 PTSD cases 39 trauma-exposed controls	Police officers	73 Dutch	38 (52.1%) Males, 35 (47.9%) Females	CAPS	No epigenome-wide significant differences in methylation levels.
Maddox et al. [17]	450 K; Blood	Cross-sectional; 109 PTSD cases, 169 trauma-exposed controls	Civilians from the GTP cohort; mixture of trauma types	278 predominately African American	278 (100%) Females	PSS	Genome-wide significant difference in methylation at one CpG site in *HDAC4*.
Rutten et al. [15]	450 K; Blood	Discovery dataset: longitudinal; 32 high PTSD, high trauma 29 low PTSD, high trauma 32 low PTSD, low trauma Replication dataset: longitudinal; 35 cases with PTSD 63 trauma exposure controls	Military soldiers with combat exposure, pre-deployment and post-deployment (minimum of 4 months) to Afghanistan from the PRISMO cohort. Marines with combat exposure, pre-deployment and post-deployment to Iraq or Afghanistan from the MRS cohort	93 Dutch Caucasian soldiers and 98 North American marines	93 Males (100%); 27.5 years and 98 Males (100%); 22 years	SRIP or CAPS	Longitudinal changes in PTSD symptoms were associated with differential methylation at CpG sites in the *DUSP22*, *NINJ2*, *HOOK2*, *SDK1*, *MYT1L*, *PAX8*, *COL1A2,* and *HIST1H2APS2* genes in the PRISMO cohort. The finding related to *HIST1H2APS2* was replicated in the MRS cohort.
Uddin et al. [21]	450 K, Blood	Cross-sectional, meta-analysis; 198 with PTSD 347 trauma-exposed controls	Civilians from the DNHS, GTP, and WTC cohorts; mixture of trauma types	343 African American, 164 Caucasian American, 38 other ethnicities (not specified)	294 Males (54%), 251 Females (46%), 46.6 years	PCL-C CAPS SCID	Epigenome-wide significant differences in methylation of CpG sites in the *NRG1* and *HGS* genes.
Logue et al. [25]	EPIC, Blood	Cross-sectional; 378 PTSD cases 135 trauma-exposed controls	War veterans exposed to combat trauma in Iraq and/or Afghanistan form the TRACTS cohorts and veterans recruited from TBI-VA-Boston	513 American veterans (not otherwise specified)	467 (91%) Males, 46 (9%) Females, 32.7 years	CAPS	Epigenome-wide significant difference in methylation of a CpG site in the *G0S2* gene.
Snijders et al. [16]	450 K; Blood	Longitudinal; 123 PTSD cases 143 trauma-exposed controls	Military (marine and army) combat exposed personnel from the MRS, STARRS, and PRISMO cohorts, deployed to Iraq or Afghanistan for 4 to 7 months	126 predominately Caucasian American marines, 78 Caucasian American army soldiers, 62 Dutch army soldiers	266 (100%) Males; 24.5 years	CAPS, PCL/CIDI-SC and SRIP	Epigenome-wide significant differences in methylation of CpG sites in the *SPRY4*, *SDK1*, *CTRC*, *CDH15*, *MAD1L1*, *HEXDC* genes.
Smith et al. [22]	450 K, Blood	Cross-sectional, meta-analysis; 878 PTSD cases 1018 trauma-exposed controls	Three civilian samples and seven combat samples all exposed to trauma including combat and various civilian traumas from the DNHS, GTP, WTC, STARRS, MRS, INTRusST, PRISMO, VA-M-EA, VA-M-AA, and VA-NCPTSD cohorts	986 Caucasian American, 62 Dutch, 777 African American, 57 Hispanic, 76 other ethnicities (not specified)	1303 (68.7%) Males, 593 (31.3%) Females, 35.8 years	PCL-C, DSM-IV, CAPS, MINI, SCID, CIDI-SC, SRIP	Epigenome-wide significant differences in methylation of CpG sites in the *AHRR*, *RNF6*, *MIR3170*, *ATP9A*, *AC011899.9*, *FLJ46321,* and *LINC00599* genes.

*HM27* HumanMethylation27 BeadChip, *PTSD* posttraumatic stress disorder, *DNHS* Detroit Neighborhood Health Study, *PCL-C* PTSD Checklist–Civilian Version, *GTP* Grady Trauma Project, *CAPS* Clinician-Administered PTSD Scale, *APC5* acid phosphatase 5, tartrate resistant, *TLR8* toll-like receptor 8, *TPR* translocated promoter region, *CLEC9A* C-type lectin domain family 9, *ANXA2* annexin A2, *450* *K* HumanMethylation 450 K BeadChip, *CT* childhood trauma, *PSS* PTSD Symptom Scale, *IPV* intimate partner violence, *GABAA* gamma-aminobutyric acid A, *WTC* World Trade Centre 9/11 responders study, *SCID* Structured Clinical Interview for DSM Disorders, *ZDHHC11* zinc finger DHHC-type containing 11, *2CSMD2* CUB and sushi domain-containing protein, *COL9A3* collagen type IX alpha 3 chai, *PDCD6IP* programmed cell death 6 interacting protein, *TBC1D24* TBC1 domain family member 24, *FAM164A* family with sequence similarity 164, member A, *EPIC* Illumina EPIC BeadChip, *BRSK1* brain-specific serine/threonine-protein kinase 1, *NGF* nerve growth factor, *LCN8* lipocalin 8, *DOCK2* dedicator of cytokinesis 2, *LRRC3B* leucine rich repeat containing 3B, *HDAC4* histone deacetylase 4, *PRISMO* Prospective Research in Stress-related Military Operations, *MRS* Marine Resiliency Study, *SRIP* Self-Rating Inventory for PTSD, *DUSP22* dual specificity phosphatase 22, NINJ2 ninjurin 2, *HOOK2* hook microtubule tethering protein 2, *SDK1* sidekick cell adhesion molecule 1, *MYT1L* myelin transcription factor 1 like, *PAX8* paired box 8, *COL1A2* collagen type I alpha 2 chain, *HIST1H2APS2* H2A histone family, member T, pseudogene, *NRG1* neuregulin 1, *HGS* hepatocyte growth factor-regulated tyrosine kinase substrate, *TRACTS* Translational Research Centre for TBI and Stress Disorders, *VA-RR&D* Department of Veterans Affairs Rehabilitation Research and Development, *TBI-VA-Boston* Traumatic Brain Injury Centre of Excellence–Veteran Affairs Boston Healthcare System, *G0S2* G0/G1 switch 2, *STARRS* Study to Assess Risk and Resiliency in Service members, *CIDI-SC* Composite International Diagnostic Interview–Screening Scales, *SPRY4* sprouty RTK signaling antagonist 4, *SDK1* sidekick cell adhesion molecule 1, *CTRC* chymotrypsin C, *CDH15* cadherin 15, *MAD1L1* mitotic arrest deficient 1 like 1, *HEXDC* hexosaminidase glycosyl hydrolase family 20 catalytic domain containing, *INTRusST* Injury and Traumatic Stress Study, *VA-M-EA* Mid-Atlantic Mental Illness Research Education and Clinical Center PTSD Study European American cohort and *VA-M-AA* African American cohort, *VA-NCPTSD* Boston Veterans Affairs National Center for PTSD, *DSM-IV* Diagnostic and Statistical Manual of Mental Disorders IV, *MINI* Mini-International Neuropsychiatric Interview, *AHRR* human aryl hydrocarbon receptor repressor, *RNF6* ring finger protein 6, *MIR3170* microRNA 3170, *ATP9A* ATPase phospholipid transporting 9A, *FLJ46321* family with sequence similarity 75, member D1, *LINC00599* long intergenic non-protein coding RNA 599.

A meta-analysis of three North American mixed-gender civilian EWASs [13, 17, 18, 20] found that PTSD was associated with the neuregulin1 *(NRG1)* and hepatocyte growth factor-regulated tyrosine kinase substrate *(HGS)*, both of which are related to central nervous system functioning [21]. The largest EWAS meta-analysis to date included 796 participants with PTSD and 1100 healthy controls [22]. North American and European male and female participants were drawn from three civilian cohorts [13, 17, 18, 20] and seven combat-exposed cohorts [15, 16] were included. Associations with PTSD were observed at four CpG sites of the human aryl hydrocarbon receptor repressor *(AHRR)* gene, which has been linked to both pro-inflammatory and anti-inflammatory immune regulation [22, 23]. Ring finger protein 6 *(RNF6)* associated with immune function, ATPase phospholipid transporting 9A *(ATP9A)*, associated with glucose metabolism, family with sequence similarity 75-like protein FLJ46321 *(FLJ46321)*, associated with cell differentiation; microRNA 3170 *(MIR3170)*, and the long intergenic non-protein coding RNA 599 *(LINC00599)* genes were also associated with PTSD [22].

None of the gene-specific findings have been replicated across EWASs. Heterogeneity between and within EWASs may explain the lack of consistent findings. The majority of EWASs have been cross-sectional studies [11–15, 17–20, 22, 24, 25] and have investigated differential methylation in combat-exposed populations and first responders [12, 14–16, 20, 24, 25]. PTSD symptoms may manifest differently in combat-exposed samples (increased hypervigilance and compulsive behavior) compared to civilian samples [26, 27]. In civilians, PTSD symptom presentation, severity and recovery rates also differ depending on trauma type [26, 28, 29]. Civilian EWASs have investigated a mixture of traumas and none have investigated rape exclusively [30]. Civilian EWASs have also been predominantly conducted in mixed-gender [11, 13, 18, 19, 25, 31], North American samples [11–13, 17–20, 25].

Ethnicity-specific and sex-specific characteristics may influence methylation profiles [32–34]. Women have a two-fold increased risk of developing PTSD compared to men [34]. Increased risk for PTSD in women may be X-chromosome linked, given that PTSD heritability is considerably higher among women compared to men [35, 36]. Sex-specific expression of reproductive genes may also mediate the increased risk for PTSD in women, for example, estrogen levels have been associated with an altered hypothalamic-pituitary-adrenal (HPA) axis stress response in women [17, 37, 38] and differential methylation of estrogen response elements (EREs) in genes associated with HPA-axis functioning has been reported [17, 39].

We sought to address the design shortcomings and demographic differences in prior EWASs by conducting a cross-sectional EWAS study, complemented by validation of the results, replication, and longitudinal investigation of a demographically similar group of rape-exposed African black women in a low-income setting. Specific aims were to: (1) identify genome-wide differentially methylated CpG sites/regions associated with PTSD status at 3-months post-rape using an EWAS approach in a discovery sample; (2) validate the significant EWAS results in selected genes using an alternate methodology; (3) replicate the findings in 2 using a larger sample; (4) determine whether methylation levels of selected genes at baseline predict PTSD status change over 6-months; and (5) determine whether methylation changes in selected genes covary with PTSD symptom scores over 6 months.

## Methods

### Participant recruitment and setting

Participants were recruited through the Rape Impact Cohort Evaluation (RICE) study conducted in South Africa (*n* = 852). A detailed description of the methods of the RICE study has been published elsewhere [40]. In short, female survivors of rape were recruited from rape clinics. Interested participants were invited to the study site to enrol in the study following informed consent procedures. Recruitment was restricted to female participants between 18 and 40 years who reported rape in the preceding 20 days of the baseline visit. In this study, we excluded women who: (1) were pregnant or lactating during the course of the study; (2) met criteria for PTSD at the baseline visit, as this would be indicative of PTSD due to a past traumatic event other than the rape; and (3) had HIV-seroconverted. Samples from 48 participants comprised the “discovery” sample, i.e., those that were included in the epigenome-wide DNA methylation analysis. These samples were subsequently utilized to technically validate the results from the EWAS study using EpiTYPER Sequenom MassARRAY technology (Agena Bioscience, California, United States). The “replication” sample comprised 96 participants, 47 from the discovery sample and 49 additional samples.

Ethical approval for the RICE parent study was obtained from the Human Research Ethics Committee at the South African Medical Research Council (SAMRC; EC019-10/2013) and approval to conduct the sub-study was obtained from the Health Research Ethics Committee at Stellenbosch University (S16/08/146).

### Clinical measures

At the baseline visit, a research assistant supervised by a registered trauma counselor or registered nurse assessed for PTSD (in relation to prior criterion A traumas other than the rape) on the Mini-International Neuropsychiatric Interview (MINI) version 7.0.0 [41]. An HIV rapid test, pregnancy test, blood collection for DNA analysis, and assessment of body mass index (BMI) were undertaken by a nurse at all time points (baseline, 3-months, and 6-months post-rape).

A research assistant administered a demographic questionnaire, a modified version of the Childhood Trauma Questionnaire-Short Form (CTQ-SF) [42], and a modified version of the Life Events Checklist (LEC) [43, 44] at baseline. The Davidson Trauma Scale (DTS) [45], the Alcohol Use Disorders Identification Test, alcohol consumption subscale (AUDIT-C) [46], and the Center for Epidemiologic Studies Depression Scale (CES-D) [47] was administered at all time points. The DTS was used to measure PTSD symptoms with a cut-off score of forty or more considered indicative of PTSD [45]. This cut-off was used to group participants into PTSD cases and controls at 3-months post-rape (see supplementary material for more details) [45]. All assessments were completed face-to-face and responses were recorded and electronically captured in real-time on a secure server. Item-level missing values were imputed using a multiple imputation model whilst maintaining a multivariate normal distribution.

### Demographic and clinical characteristics of the sample

The baseline demographic and clinical characteristics of the sample were investigated using descriptive statistics. Differences in baseline demographic and clinical characteristics between the discovery/validation sample and the replication sample were investigated using non-parametric tests since most of the variables did not conform to a normal distribution. Mann–Whitney U tests were used to compare groups on continuous variables, i.e., age, body mass index (BMI), childhood trauma score, number of childhood traumas endorsed, number of lifetime traumas endorsed, alcohol use, and depression symptom scores. Chi-square statistics were used to compare groups on several categorical variables (completed secondary education, relationship status, smoking status, HIV status, medication use, childhood neglect, witnessed domestic violence in the childhood home, childhood emotional abuse, childhood physical abuse, childhood sexual abuse, imprisonment, civil unrest or war, serious injury, being close to death, murder of a family member or friend, unnatural death of a family member or friend, murder of a stranger, robbed at gun/knifepoint, kidnaped, hazardous alcohol use and depression status).

The same variables and methods used to investigate baseline demographic and clinical differences between the discovery/validation and replication samples were used to investigate differences between those with and without PTSD at 3-months post-rape.

### Cross-sectional analyses (3 months post-rape)

#### Discovery sample

Forty-eight participants, 24 with PTSD and 24 without PTSD at 3-months post-rape, were included in the discovery sample. We selected the 3-months post-rape time point since it was the first time point, in the parent study, at which a PTSD diagnosis could be made, based on DSM-5 criteria [48] We implemented a cross-sectional, case-control design to identify genome-wide differentially methylated positions (DMPs) and differentially methylated regions (DMRs) between individuals with and without PTSD. Consecutive cases of PTSD at 3-months post-rape were identified until the target number was reached. Controls were perfectly matched to cases, based on HIV status and as closely as possible (in descending hierarchical order of importance) on age, childhood trauma scores, lifetime trauma exposure, BMI, smoking, education, and income. DNA was extracted from peripheral blood samples and assayed using the Human Illumina EPIC BeadChip array (Illumina, California, United States) [49].

Raw probe intensity data (iDAT) files produced by Illumina GenomeStudio were decompressed and parsed into text format using the *meffil* R package [50] in R statistics version 3.6.2 [51]. All EWAS analyses, including quality control measures and beta normalization, were completed using the *meffil* R package [50].

All samples passed the quality control checks (see Supplementary Material for more details). Probes not passing the quality control checks (*n* = 29936) were excluded from the downstream analyses. Previously identified cross-reactive probes for 43254 CpG sites were also excluded [52]. Probes targeting CpG sites on the X chromosome were retained since all participants included in the study were female.

The percentage of methylated alleles for each CpG site in each sample was calculated as *β* = *M*/(*M* + *U* + 100) where *M* and *U* symbolize raw probe fluorescent intensities for methylated and unmethylated signals, respectively [53]. Technical bias and batch effects were corrected for using functional normalization (Supplementary Material, Supplementary Figs. 1–5, Supplementary Tables 1 and 2) [54]. Any residual effects were handled by including surrogate variables as covariates in the EWAS models. These were estimated following functional normalization using surrogate variable analysis (SVA) [55]. Cell type composition was estimated by applying the Houseman algorithm to the normalized DNA methylation profiles and a publicly available blood cell type reference dataset (Gene Expression Omnibus accession number GSE35069) [56]. Cell type composition was included in the final EWAS models (Supplementary Fig. 6). Epigenome-wide associations were investigated using logistic regression models to identify DMPs associated with PTSD status. A Bonferroni correction was applied to correct for multiple testing with an adjusted *p*-value < 0.05 indicating genome-wide significance [57].

The *dmrff* R package was applied to EWAS summary statistics to identify DMRs [58]. DMRs were defined as a region covering two or more CpG sites with less than 100 bp between consecutive sites showing the same direction of effect with an uncorrected *p*-value < 0.05 (see supplementary material for more details) [58]. A DMR was considered significant on an epigenome-wide level if a Bonferroni-adjusted *p* < 0.05 was observed. Coordinates resulting from the DMP and DMR analyses were annotated using the Illumina EPIC_v-1-0_B4 manifest [53]. Co-variation in methylation levels between blood and brain tissue was explored using the online Blood-Brain DNA Methylation Comparison Tool [59]. Prior findings reporting a link between any exposure or phenotype and the CpG sites identified from the EWAS were identified using the Medical Research Council Integrative Epidemiology Unit (MRC-IEU) catalog of epigenome-wide association studies [60] and the China National Center for Bioinformation National Genomics Data Center epigenome-wide association studies atlas [61]. Prior findings reporting a link between mood, anxiety, or trauma-related disorders and any CpG site in the genes identified from the EWAS were identified using the aforementioned databases for EWAS studies, and the European Molecular Biology Laboratory-European Bioinformatics Institute (EMBL-EBI) genome-wide association study (GWAS) Catalog for GWAS studies [62]. Prior findings reporting a link between PTSD and any CpG site in the genes identified from the EWAS were identified through a literature search in PubMed [63]. All genomic coordinates reported in this study are in reference to the Hg19/GRCh37 human genome assembly (see Supplementary Material for more details).

#### Validation analysis

A candidate gene approach was used to validate the findings of the EWAS in 47 of the 48 participants included in the discovery sample. One participant was excluded from the validation sample due to incomplete data at the time of validation analysis. Samples were assayed using EpiTYPER. DNA methylation was investigated at CpG sites in two selected regions at 3-months post-rape. Brain-specific serine/threonine-protein kinase 2 (*BRSK2)* and adenylate cyclase-activating polypeptide 1 *(ADCYAP1)* were selected for validation since they contained CpG sites found to be differentially methylated (prior to correction for multiple testing) between cases and controls in the EWAS. Both genes were also found to contain differentially methylated regions. A CpG site in *BRSK1*, a paralog of *BRSK2*, was found to be differentially methylated in a prior PTSD EWAS study [14] and *ADCYAP1* receptor 1 *(ADCYAP1R1)* has been linked to the development of PTSD in several prior studies [64–66].

DNA methylation percentages were exported using the EpiTYPER Analyzer software. The validation analyses were completed using IBM SPSS Statistics 27.0. Logistic regression models were used to determine if differential methylation of *BRSK2* and *ADCYAP1* at 3-months post-rape was associated with PTSD status at 3-months post-rape.

The relationship between baseline confounding variables, PTSD status at 3-months post-rape, *BRSK2* methylation at 3-months post-rape, and *ADCYAP1* methylation at 3-months post-rape was investigated using Mann−Whitney U tests, Chi-square tests, and Spearman’s correlations. Potential confounders included continuous variables (age, BMI, childhood trauma score, number of lifetime traumas endorsed, alcohol use, and depression) and categorical variables (HIV status, smoking, and medication use). Confounding variables significantly associated with PTSD or *BRSK2/ADCYAP1* methylation were entered in logistic regression models as covariates, in a stepwise manner.

#### Replication analysis

To replicate the validation analysis, an additional 49 consecutively selected participants from the parent study were included in the DNA methylation replication analyses. These participants were not matched on PTSD status or potential methylation covariates. Samples were assayed using EpiTYPER.

Logistic regression models, including potential confounding variables, were used to determine if differential methylation of *BRSK2* and *ADCYAP1* at 3-months post-rape was associated with PTSD status at 3-months post-rape in the replication sample, following the same procedure applied in the validation analyses.

#### Comparison of previous findings from candidate gene studies and EWASs

Candidate gene studies and EWASs investigating the relationship between methylation and PTSD were identified from published literature. For EWASs, the Illumina CpG identification number for significant findings was manually recorded and cross-checked against the findings of the current EWAS. For candidate gene studies, the genomic coordinates of the sites were identified from the publications and converted to Hg19/GRCh37 positions using the BLAT function of the University of California, Santa Cruz (UCSC) genome browser (if not already indicated as Hg19/GrCh37 positions). The genomic locations were manually recorded and cross-checked with the Illumina EPIC_v-1-0_B4 manifest to determine if the sites were included on the Illumina EPIC array. Significant CpG sites resulting from the current EWAS and corresponding to prior findings are reported in the results.

#### Agreement between the Illumina EPIC array and EpiTYPER

Spearman’s correlation coefficients were used to investigate the level of agreement between methylation levels resulting from the Illumina EPIC array at 3-months post-rape and methylation levels resulting from EpiTYPER at 3-months post-rape.

### Longitudinal investigation (baseline, 3-months, and 6-months post-rape)

#### Combined sample

The validation and replication samples were combined and methylation data from the baseline and 6-month post-rape samples were added to the dataset, for the same combined group. The group consisted of 96 participants with methylation data at all time points (baseline, 3-months, and 6-months). The samples were assayed using EpiTYPER. We investigated the same *BRSK2* and *ADCYAP1* CpG sites investigated in the validation and replication samples but followed a longitudinal cohort design with PTSD symptom scores as the outcome, instead of a cross-sectional case-control design with PTSD status at 3-months as the outcome.

PTSD scores at each time point were compared between the discovery/validation sample and the replication sample using Mann-Whitney U tests. The relationship between PTSD, *BRSK2* methylation, *ADCYAP1* methylation (at all time points), and potential baseline confounders (age, BMI, childhood trauma, lifetime traumas, alcohol use, depression, HIV status, smoking, and medication use) was investigated using Mann-Whitney U tests, Chi-square tests and Spearman’s correlations.

Baseline *ADCYAP1* and *BRSK2* methylation levels were investigated as predictors of change in PTSD symptom scores over six months, in the first set of mixed regression models. In the second set of mixed regression models, we investigated change in *BRSK2* and *ADCYAP1* methylation levels over six months in relation to change in PTSD symptom scores over six months. Confounding variables significantly associated with PTSD or *BRSK2/ADCYAP1* methylation at any time point were entered in the mixed regression models as covariates, in a stepwise manner.

## Results

### Baseline demographic and clinical characteristics of the sample

Table 2 presents the baseline demographic and clinical characteristics of the discovery/validation and replication samples. The samples were similar with regard to demographic and clinical characteristics. The only variable that differed between the samples was the prevalence of lifetime exposure to the murder of a family member or friend, which was more frequently endorsed in the discovery/validation sample compared to the replication sample (25.5% vs. 8.2%, respectively; *χ*^*2*^ = 5.2, *p* = 0.022).

**Table 2:** Baseline demographic and clinical characteristics of the discovery/validation and replication samples.

	Discovery/validation sample (*n* = 47)		Replication sample (*n* = 49)		Comparison of discovery/validation sample to replication sample		
	*n* (%)	*M (SD)*	*n* (%)	*M (SD)*	*χ*^2^	*z*	*p*
Age^a^	47 (100)	25.9 (5.4)	49 (100)	24.6 (5.5)		−1.3	0.178
Secondary education completed^b^	32 (68.1)		25 (51)		2.9		0.089
Employed^b^	13 (27.7)		9 (18.4)		1.2		0.279
In a relationship/married^b^	38 (80.9)		38 (77.6)		0.2		0.691
BMI^a^	47 (100)	26.0 (6.5)	49 (100)	25.8 (5.7)		−0.1	0.956
Smoker^b^	5 (10.6)		7 (14.3)		0.3		0.589
HIV positive^b^	27 (57.4)		19 (38.8)		3.4		0.067
On ARVs^b^	12 (25.5)		14 (28.6)		0.1		0.738
On medications for STI^b^	2 (4.3)		2 (4.1)		0.0		0.966
Other medication use^b,c^	1 (2.1)		2 (4.1)		0.3		0.582
Childhood trauma score^a^	47 (100)	17.2 (4.1)	49 (100)	16.2 (2.5)		−0.8	0.410
Neglect^b^	23 (48.9)		18 (36.7)		1.5		0.227
Domestic violence^b^	10 (21.3)		8 (16.3)		0.4		0.534
Emotional abuse^b^	12 (25.5)		11 (22.4)		0.1		0.724
Physical abuse^b^	18 (38.3)		19 (38.8)		0.0		0.962
Sexual abuse^b^	10 (21.3)		11 (22.4)		0.0		0.890
Number of childhood traumas^a^	47 (100)	1.6 (1.6)	49 (100)	1.4 (1.5)		−0.6	0.530
Number of lifetime traumas^a,d^	47 (100)	1.6 (1.5)	49 (100)	1.13(1.2)		−1.7	0.092
Imprisonment^b^	2 (4.3)		1 (2.0)		0.4		0.533
Civil unrest or war^b^	3 (6.4)		1(2.0)		1.1		0.287
Serious injury^b^	8 (17.0)		3 (6.1)		2.8		0.094
Being close to death^b^	13 (27.7)		14 (28.6)		0.0		0.921
Murder of family/friend^b^	12 (25.5)		4 (8.2)		5.2		0.022*
Unnatural death of family/friend^b^	9 (19.1)		5 (10.2)		1.5		0.214
Murder of stranger^b^	10 (21.3)		5 (10.2)		2.2		0.135
Robbed with gun/knife used^b^	17 (36.2)		18 (36.7)		0.0		0.954
Kidnaped^b^	3 (6.4)		4 (8.2)		0.1		0.737
PTSD symptom score^a^	47 (100)	67.1 (21.7)	49 (100)	65.7 (18.6)		−0.8	0.431
Alcohol use severity score^a^	47 (100)	1.4 (2.2)	49 (100)	1.9 (2.5)		−1.2	0.242
Hazardous alcohol use^b^	12 (25.5)		15 (30.6)		0.3		0.580
Depression symptom score^a^	47 (100)	32.4 (13.9)	49(100)	31.7 (12.1)		−0.2	0.854
Depression status^b^	41 (87.2)		45 (91.8)		0.5		0.461

*PTSD* Posttaumatic stress disorder*,* *M* mean, *SD* standard deviation, *BMI* body mass index, *ARV* antiretrovirals, *STI* sexually transmitted infection.

^a^Continous variables.

^b^Categorical variables.

^c^Medication prescribed for chronic sinusitis (*n* = 1) and hypertension (*n* = 2).

^d^Lifetime traumas refer to directly experiencing the trauma; *p < 0.05.

### Comparison of baseline demographic and clinical characteristics between the PTSD groups at 3-months post-rape

Table 3 presents group comparisons by PTSD status (at 3-months post-rape) in the discovery/validation sample and the replication sample, consecutively. Participants with and without PTSD had similar baseline demographic and clinical characteristics in the discovery/validation and replication samples. However, in the discovery/validation sample, those with PTSD were more likely to endorse being robbed with a gun or knife compared to those without PTSD (50% and 21.7%, respectively; *z* = 4.1, *p* = 0.044). In the replication sample, those with PTSD endorsed less lifetime traumas (*M* = 0.5, *SD* = 0.7) compared to those without PTSD (*M* = 1.4, *SD* = 1.3, *z* = −2.5, *p* = 0.014).

**Table 3:** Baseline demographic and clinical characteristics of rape-exposed participants with and without posttraumatic stress disorder at 3-months post-rape in the discovery/validation and replication samples.

	Discovery/validation sample (*n* = 47)							Replication sample (*n* = 49)						
	With PTSD at 3-months^a^ (*n* = 24)		Without PTSD at 3-months^a^ (*n* = 23)		Group difference			With PTSD at 3-months^a^ (*n* = 15)		Without PTSD at 3-months^a^ (*n* = 34)		Group difference		
	*n* (%)	*M (SD)*	*n* (%)	*M (SD)*	*χ*^*2*^	*z*	*p*	*n* (%)	*M (SD)*	*n* (%)	*M (SD)*	*χ*^*2*^	*z*	*p*
Age^b^	24 (100)	25.1 (5.3)	23 (100)	26.7 (5.5)		−1.0	0.296	15 (100)	24.7 (4.7)	34 (100)	24.5 (5.9)		−0.5	0.616
Secondary education completed^c^	16 (66.7)		16 (69.6)		0.1		0.831	10 (66.7)		15 (44.1)		2.1		0.146
Employed^c^	4 (16.7)		9 (39.1)		3.0		0.085	1 (6.7)		8 (23.5)		2.0		0.160
In a relationship/married^c^	19 (79.2)		19 (82.6)		0.1		0.764	11 (77.3)		27 (79.4)		0.2		0.638
BMI^b^	24 (100)	24.8 (5.4)	23 (100)	27.2 (7.4)		−1.1	0.268	15 (100)	25.3 (4.8)	34 (100)	26.0 (6.1)		−0.3	0.745
Smoker^c^	3 (12.5)		2 (8.6)		0.2		0.672	2 (13.3)		5 (14.7)		0.0		0.899
HIV positive^c^	14 (58.3)		13 (56.5)		0.0		0.900	7 (46.7)		12 (35.3)		0.6		0.451
On ARVs^c^	6 (25.0)		6 (26.1)		0.0		0.932	4 (26.7)		10 (29.4)		0.0		0.845
On medications for STI^c^	1 (4.2)		1 (4.3)		0.0		0.975	0 (0.0)		2 (5.9)		0.9		0.338
Other medication use^c,d^	0 (0.0)		1 (4.3)		1.1		0.302	0 (0.0)		2 (5.9)		0.9		0.338
Childhood trauma score^b^	24 (100)	18.2 (4.6)	23 (100)	16.2 (3.3)		−1.7	0.098	15 (100)	15.7 (2.5)	34 (100)	16.4 (2.6)		−1.0	0.299
Neglect^c^	13 (54.2)		10 (43.5)		0.5		0.464	5 (33.3)		13 (38.2)		0.1		0.743
Domestic violence^c^	7 (29.2)		3 (13.0)		1.8		0.177	3 (20.0)		5 (14.7)		0.2		0.644
Emotional abuse^c^	9 (37.5)		3 (13.0)		3.7		0.055	3 (20.0)		8 (23.5)		0.1		0.785
Physical abuse^c^	10 (41.7)		8 (34.8)		0.2		0.627	5 (33.3)		14 (41.2)		0.3		0.604
Sexual abuse^c^	7 (29.2)		3 (13.0)		1.8		0.177	2 (13.3)		9 (26.5)		1.0		0.310
Number of childhood traumas^b^	24 (100)	1.9 (1.7)	23 (100)	1.2 (1.3)		−1.6	0.120	15 (100)	1.2 (1.7)	34 (100)	1.4 (1.5)		−0.8	0.430
Number of lifetime traumas^b,e^	24 (100)	2.0 (1.6)	23 (100)	1.2 (1.2)		−1.9	0.063	15 (100)	0.5 (0.7)	34 (100)	1.4 (1.3)		−2.5	0.014
Imprisonment^c^	2 (8.3)		0 (0.0)		2.0		0.157	0 (0.0)		1 (2.9)		0.5		0.502
Civil unrest or war^c^	2 (8.3)		1 (4.3)		0.3		0.576	0 (0.0)		1 (2.9)		0.5		0.502
Serious injury^c^	6 (25.0)		2 (8.6)		2.2		0.137	1 (6.7)		2 (5.9)		0.0		0.916
Being close to death^c^	7 (29.2)		6 (26.1)		0.1		0.813	3 (20.0)		11 (32.4)		0.8		0.378
Murder of family/friend^c^	5 (20.8)		7 (30.4)		0.6		0.450	0 (0.0)		4 (11.8)		1.9		0.166
Unnatural death of family/friend^c^	5 (20.8)		4 (17.4)		0.1		0.764	0 (0.0)		5 (14.7)		2.5		0.117
Murder of stranger^c^	7 (29.2)		3 (13.0)			1.8	0.177	0 (0.0)		5 (14.7)		2.5		0.117
Robbed with a gun/knife used^c^	12 (50.0)		5 (21.7)			4.1	0.044	3 (20.0)		15 (44.1)		2.6		0.107
Kidnaped^c^	3 (12.5)		0 (0.0)			3.1	0.080	1 (6.7)		3 (8.8)		0.1		0.799
PTSD symptom score^b^	24 (100)	75.7 (17.9)	23 (100)	58.1 (22.0)		−2.9	0.004	15 (100)	63.4 (20.4)	34 (100)	66.7 (18.0)	−0.1		0.914
Alcohol use severity score^b^	24 (100)	1.7 (2.4)	23 (100)	1.2 (1.9)		−0.9	0.394	15 (100)	1.6 (2.6)	34 (100)	2.1 (2.5)		−1.0	0.299
Hazardous alcohol use^c^	7 (29.2)		5 (21.7)			0.3	0.559	4 (26.7)		11 (32.4)		0.2		0.691
Depression symptom score^b^	24 (100)	35.1 (12.9)	23 (100)	29.5 (14.7)		−1.4	0.173	15 (100)	28.4 (13.5)	34 (100)	33.1 (11.4)		−1.4	0.149
Depression status^c^	22 (91.7)		19 (82.6)			0.9	0.352	13 (86.7)		32 (94.1)		0.8		0.380

*PTSD* Posttraumatic stress disorder, *M* mean, *SD* standard deviation, *BMI* body mass index, *ARV* antiretrovirals, *STI* sexually transmitted infection.

^a^The 3-month post-rape time point was used in the analysis since it is the first time point in the parent study at which a PTSD diagnosis can be made. PTSD status at 3-months post-rape was used as the outcome to address the first three aims of the study. PTSD symptom score rather than PTSD status was used as the outcome in the longitudinal analysis to address aim four and aim five of the study. One participant included in the discovery sample (*n* = 48) was not included in the validation sample (*n* = 47).

^b^Continous variables.

^c^Categorical variables.

^d^Medication prescribed for chronic sinusitis (*n* = 1) and hypertension (*n* = 2).

^e^Lifetme traumas refer to directly experiencing the trauma.

### Discovery sample: genome-wide differentially methylated genes associated with PTSD status at 3-months post-rape

Table 4 presents selected findings from the top twenty DMPs that were associated with PTSD before correction for multiple comparisons (*p* < 0.05) (see Supplementary Table 3 and Supplementary Figs. 7–9 for more details). Only one DMP, cg01700569, remained significant after correcting for multiple testing (adjusted *p* < 0.05). This intergenic site (cg01700569) is located 24694 bases downstream of solute carrier family 16 member 9 (*SLC16A9*). Other genes previously linked to mood, anxiety, or trauma-related disorders included protein zeta-1 *(FEZ1)*, *ADCYAP1, BRSK2*, catenin alpha 3 *(CTNNA3),* and par-3 family cell polarity regulator *(PARD3)*.

**Table 4:** Genome-wide differentially methylated positions (DMPs) and regions (DMRs) associated with posttraumatic stress disorder in the discovery sample.

Gene Name^a^	Position^b^	Probe	Location in Gene^c,d^	*β*	SE	*t/z*	*p*	Adj. *p*	Other exposures/phenotypes associated with the CpG site^e^	Mood, anxiety or trauma-related disorders previously associated with the site in EWAS or GWAS studies^e,f^	Reference of prior candidate gene study linking the gene to PTSD^g^
*Differentially methylated positions (DMPs)*											
NA, *SLC16A9*^h^	Chr10:61385771	cg01700569	Intergenic	0.031	0.004	7.119	6.187e−08	0.049233	None	NA	NA
*FEZ1*	Chr11:125365803	cg06309855	5’UTR	0.022	0.004	5.727	2.930e−06	0.777284	Gestational age	Depression, Bipolar	None
*ADCYAP1*	Chr18:905177	cg22388954	5’UTR; TSS200	−0.025	0.005	−5.181	1.371e−05	0.999998	B acute lymphoblastic leukemia	None	Ressler et al. [64]
*BRSK2*	Chr11:1431833	cg09450823	Body	0.036	0.007	5.115	1.653e−05	0.999998	None	PTSD (paralog *BRSK1*)	None
*CTNNA3*	Chr10:68940214	cg23307744	Body	−0.012	0.002	−5.012	2.208e−05	0.999998	None	Depression	None
*PARD3*	Chr10:35016204	cg18026072	Body	−0.009	0.002	−4.961	2.534e−05	0.999998	None	Depression	None
*Differentially methylated regions (DMRs)*											
*CC2D2A*	Chr4:15471214-15471399			0.015	0.002	7.856	3.964e−15	3.285e−09		Depression	None
		cg21329975	TSS1500						Smoking status; air population exposure; gestational age		
		cg16509355	TSS1500						Fetal vs. adult liver; smoking status; gestational age; Down syndrome		
		cg21123203	Intergenic						Smoking status; fruit consumption		
		cg02964094	TSS200						Gingivobuccal oral squamous cell carcinoma; Gulf War illness		
		cg18470593	TSS200						Fetal vs. adult liver; smoking status; obesity		
		cg20184469	TSS200						None		
*BRSK2*	Chr11:1463541-1463670			0.112	0.016	7.131	9.935e−13	8e−07		PTSD (paralog *BRSK1*)	None
		cg12186219	Body						Childhood stress; ethnicity		
		cg14064268	Body						Aging; childhood stress; ethnicity		
		cg10590925	Body						Aging; childhood stress; ethnicity		
		cg17429870	Body						Aging; childhood stress; ethnicity		
		cg18651858	Body						Ethnicity		
*ADCYAP1*	Chr18:905177-905180			−0.022	0.003	−6.761	1.370e−11	1.13e−05		None	Ressler et al. [64]
		cg22388954	5’UTR; TSS200						B Acute lymphoblastic leukemia		
		cg11773720	5’UTR; TSS200						None		

*SE* Standard error, *Adj* adjusted, *EWAS* epigenome-wide association study, *GWAS* genome-wide association study, *PTSD* posttraumatic stress disorder, *SLC16A9* solute carrier family 16 member 9, *Chr* chromosome, *NA* not applicable, *FEZ1* fasciculation and elongation protein zeta 1, *5’UTR* 5’ untranslated region, *ADCYAP1* adenylate cyclase activating polypeptide 1, *TSS200* transcription start site 200, *BRSK2* brain-specific serine/threonine-protein kinase 2, *BRSK1* brain-specific serine/threonine-protein kinase 1, *MCEE* methylmalonyl-CoA epimerase, *CTNNA3* catenin alpha 3, *PARD3* PAR-3 family cell polarity regulator, *CC2D2A* coiled-coil and C2 domain-containing protein 2A, *TSS1500* transcription start site 1500.

^a^Identified using the GENECODE database.

^b^identified using the Human Genome 19 (HG19) build from the Genome Reference Consortium.

^c^identified using the University of California Santa Cruz (UCSC) Genomic Institute/Genome Browser.

^d^multiple listings indicate splice variants.

^e^identified using the Medical Research Council’s Integrative Epidemiology Unit (MRC-IEU) catalog of epigenome-wide association studies (EWAS) [60] and the China National Center for Bioinformation National Genomics Data Center EWAS atlas [61].

^f^identified using the European Molecular Biology Laboratory-European Bioinformatic Institute (EMBL-EBI) genome-wide association studies (GWAS) Catalog for GWAS studies [62].

^g^identified through a literature search in PubMed [63].

^h^CpG sites located in a region not attributed to a gene, the gene closest to the CpG site is provided.

Thirty-four DMRs were identified from the regional analysis after Bonferroni correction for multiple testing. The regions previously linked to mood, anxiety, or trauma-related disorders included coiled-coil and C2 domain-containing protein 2 A *(CC2D2A)*, *BRSK2,* and *ADCYAP1*. The findings related to these genes are also presented in Table 4.

### Validation and replication sample: differential methylation of *BRSK2* in relation to PTSD status at 3-months post-rape

**Fig. 1:**
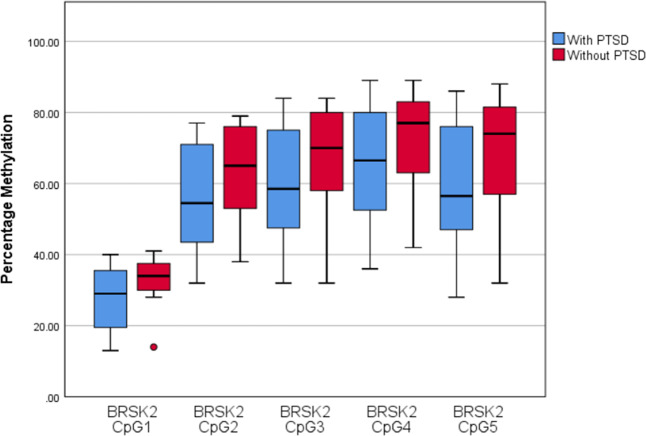
Posttraumatic stress disorder (PTSD) status and BRSK2 methylation percentage. Boxplots indicating methylation levels between participants with and without PTSD for the five CpG sites in the *BRSK2* region found to be associated with PTSD at 3-months post-rape in the epigenome-wide association study.

The *BRSK2* region (chr11:1463541-1463670; adjusted *p* < 0.05) identified from the EWAS included five CpG sites (CpG1-cg12186219, CpG2-cg14064268, CpG3-cg10590925, CpG4-cg17429870, CpG5-cg18651858) that showed decreased methylation in participants with PTSD (see Fig. 1). Based on prior findings, DNA methylation of these CpG sites in blood was highly correlated with DNA methylation in the prefrontal cortex, superior temporal gyrus, and the cerebellum (see Supplementary Fig. 10a–d) [59] Three of the five CpG sites (CpG3, CpG4, and CpG5) were investigated in the validation and replication sample. We could not investigate CpG1 or CpG2, as the mass of CpG1 was too low to be measured by the EpiTYPER mass spectrometer, and CpG2 contained a silent peak that overlapped with the non-methylated peak for this site (see Supplementary Table 4 for the genomic coordinates and sequence for CpG3, CpG4, and CpG5).

Baseline age, HIV status, BMI, smoking status, childhood trauma score, lifetime trauma, alcohol use, depression, and medication use were not associated with *BRSK2* methylation at 3-months post-rape in either the validation or replication samples. PTSD status at 3-months post-rape was associated with lifetime trauma (*z* = −2.47, *p* = 0.014) in the replication sample only (see Supplementary Tables 5 and 6).

In the validation analysis, methylation levels of *BRSK2* CpG3 (*β* = −0.04, *p* = 0.050, OR 0.96) and CpG4 (*β* = −0.04, *p* = 0.052, OR 0.96) at 3-months post-rape were not significantly associated with PTSD status at 3-months post-rape. Decreased methylation of *BRSK2* CpG5 (*β* = −0.04, *p* = 0.048, OR 0.96) at 3-months post-rape was significantly associated with PTSD status at 3-months post-rape, but the association was no longer significant when lifetime trauma was added as a covariate to the model (see Supplementary Tables 7). In the replication analysis, methylation levels of *BRSK2* CpG3 (*β* = −0.00, *p* = 0.889, OR 1.00), CpG4 (*β* = −0.01, *p* = 0.667, OR 0.99) and CpG5 (*β* = 0.00, *p* = 0.866, OR 1.00) were not significantly associated with PTSD status at 3-months post-rape (see Supplementary Table 8).

### Validation and replication samples: differential methylation of *ADCYAP1* in relation to PTSD status at 3-months post-rape

**Fig. 2:**
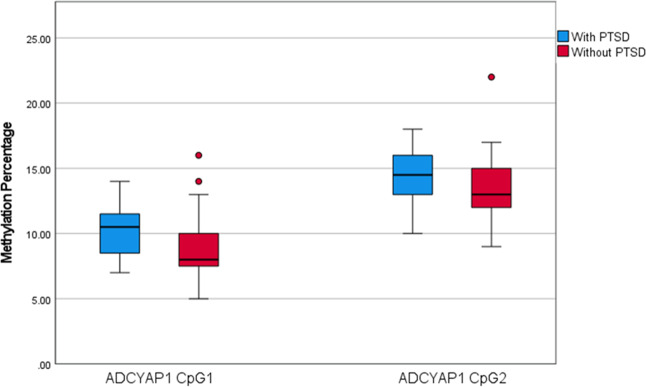
Posttraumatic stress disorder (PTSD) status and ADCYAP1 methylation percentage. Boxplots indicating methylation levels between participants with and without PTSD for the two CpG sites in the *ADCYAP1* region were found to be associated with PTSD in the epigenome-wide methylation study.

The *ADCYAP1* region (chr18:905177-905180) identified from the EWAS included only two differentially methylated CpG sites (CpG1 – cg22388954, CpG2 – cg11773720) which both showed increased methylation in participants with PTSD (see Fig. 2). Based on prior findings, DNA methylation of these CpG sites in blood was not correlated with DNA methylation in brain tissue (Supplementary Fig. 11a, b) [59]. EpiTYPER signals for *ADCYAP1* CpG1 and CpG2 were combined for analysis, due to their proximity to each other (see supplementary Table 9 for the genomic coordinates and sequence of CpG1 and CpG2).

Baseline age, HIV status, BMI, smoking status, childhood trauma score, lifetime trauma, alcohol use, depression, and medication use were not associated with *ADCYAP1* methylation at 3-months post-rape in the validation or replication samples (see Supplementary Tables 5 and 6). In the validation analysis, methylation levels of *ADCYAP1* CpG1&2 (*β* = −0.09, *p* = 0.382, OR 0.92) were not significantly associated with PTSD status at 3-months post-rape (see supplementary Tables 7). In the replication sample, methylation levels of *ADCYAP1* CpG1&2 (*β* = −0.06, *p* = 0.639, OR 0.94) were also not significantly associated with PTSD status at 3-months post-rape (see supplementary Table 8).

### Agreement between the Illumina EPIC array and EpiTYPER

Large positive correlations were found when comparing the Illumina EPIC array and EpiTYPER methylation levels for *BRSK2* CpG3 (*r* = 0.881, *p* < 0.000), CpG4 (*r* = 0.900, *p* < 0.000), and CpG5 (*r* = 0.831, *p* = 0.831) at 3-months post-rape (see Supplementary Table 10). Small, non-significant correlations were found when comparing the Illumina EPIC array and EpiTYPER methylation levels for *ADCYAP1* CpG1&2 (*r* = 0.254, *p* > 0.05; see Supplementary Table 11).

### Replication of previous candidate gene and EWAS findings

Differential methylation of five CpG sites previously investigated was replicated in this EWAS study, prior to correction for multiple testing (see Supplementary Table 12 and 13). These sites were located in the *HTR3A* (chr11:113846004, cg20621129, *p* = 0.028) [67], *AHRR* (two CpG sites: chr5:373378, cg05575921, *p* = 0.033; chr5:377358, cg26703534, *p* = 0.031) [22], *DUSP22* (chr6:291882, cg21548813, *p* = 0.032) [15] and *TPR* (chr1:186344558, cg24577137, *p* = 0.0008) genes [13]. Since decreased methylation of *AHRR* is strongly linked to smoking, [22] we investigated the link between smoking and *AHRR* methylation (based on the values obtained from our EWAS) and found decreased *AHRR* methylation levels in smokers (*M* = 78.91, *SD* = 14.95, n = 5) compared to non-smokers (*M* = 93.88, *SD* = 1.45, n = 42) at cg05575921 (*z* = −2.92, *p* = 0.001).

### Combined sample: longitudinal relationship between *BRSK2*, *ADCYAP1*, PTSD scores, and confounding variables

Baseline childhood trauma, alcohol use, and depression were associated with PTSD scores at one or more time points. Baseline childhood and lifetime trauma scores were associated with *BRSK2* methylation at one or more time points. Baseline HIV status was associated with *ADCYAP1* methylation at 3-months post-rape (see supplementary Table 14).

### Combined sample: longitudinal change in PTSD symptom scores

**Fig. 3:**
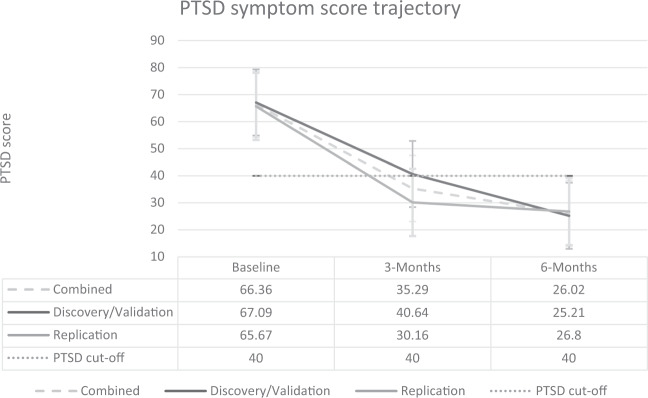
Posttraumatic stress disorder (PTSD) symptom trajectory. Symptoms over 6 months for the discovery/validation samples, replication sample, and combined sample.

The mean PTSD scores at baseline, 3-months, and 6-months, stratified by sample (discovery/validation, replication, combined), are presented in Fig. 3. There were no significant differences between the discovery/validation samples and the replication sample for either baseline (*z* = −0.79, *p* = 0.431), 3-month (*z* = −1.37, *p* = 0.172), or 6-month (*z* = −0.15, *p* = 0.883) PTSD scores. There was a significant decline in PTSD scores from baseline to 3-months (*p* < 0.000) and from 3-months to 6-months (*p* = 0.021), in the combined sample.

### Combined sample: baseline *BRSK2* and *ADCYAP1* methylation levels and longitudinal change in PTSD scores

Table 5 presents the results of the mixed regression models investigating baseline *BRSK2* and *ADCYAP1* methylation as predictors of change in PTSD symptom scores over time. Decreased baseline *BRSK2* CpG3, CpG4, and CpG5 methylation levels were significant predictors of increased PTSD symptom scores at 3-months (CpG3 *ß* = −0.39, *p* < 0.001, CpG4 *ß* = −0.33, *p* = 0.005, CpG5 *ß* = −0.27, *p* = 0.009) and 6-months (CpG3 *ß* = −0.49, *p* < 0.001, CpG4 *ß* = −0.44, *p* < 0.001, CpG5 *ß* = −0.38, *p* < 0.001) post-rape. However, the relationships between *BRSK2* CpG3, CpG4, and CpG5 methylation levels and PTSD scores at 3-and 6-months post-rape were no longer significant when childhood trauma, alcohol consumption, depression, and lifetime trauma were added to the models as covariates.

**Table 5:** Summary statistics of the mixed regression models investigating baseline *BRSK2* and *ADCYAP1* methylation as predictors of change in posttraumatic stress symptoms scores over time.

Model		*ß*	Std error	*t*	*p*	95% CI	
						Lower	Upper
	*Baseline BRSK2 CpG3 methylation*						
1A	Baseline × CpG3 (baseline)	0.07	0.10	0.71	0.482	−0.13	0.27
	3-months × CpG3 (baseline)	−0.39	0.10	−3.81	0.0002*	−0.60	−0.19
	6-months × CpG3 (baseline)	−0.49	0.10	−4.76	0.000004*	−0.70	−0.29
1B	Baseline × CpG3 (baseline)	−0.12	0.10	−1.17	0.247	−0.33	0.08
	3-months × CpG3 (baseline)	−0.16	0.15	−1.09	0.276	−0.45	0.13
	6-months × CpG3 (baseline)	−0.12	0.15	−0.81	0.418	−0.41	0.17
	Baseline × childhood trauma	1.54	0.49	3.13	0.002*	0.57	2.51
	3-months × childhood trauma	1.21	0.66	1.84	0.069	−0.09	2.52
	6-months × childhood trauma	−035	0.66	−0.53	0.598	−1.65	0.96
	Baseline × alcohol consumption	−1.36	0.76	−1.79	0.077	−2.87	0.15
	3-months × alcohol consumption	−0.96	1.23	−0.78	0.438	−3.40	1.48
	6-months × alcohol consumption	−1.65	1.23	−1.35	0.181	−4.09	0.78
	Baseline × depression	0.73	0.14	5.29	0.0000008*	0.46	1.01
	3-months × depression	0.00	0.22	0.00	1.00	−0.45	0.45
	6-months × depression	0.49	0.22	2.19	0.031*	0.05	0.93
1C	Baseline × CpG3 (baseline)	−0.10	0.10	−1.01	0.314	−0.31	0.10
	3-months × CpG3 (baseline)	−0.15	0.15	−1.03	0.305	−0.44	0.14
	6-months × CpG3 (baseline)	−0.10	0.15	−0.68	0.496	−0.39	0.19
	Baseline × childhood trauma	1.12	0.52	2.45	0.016*	0.24	2.30
	3-months × childhood trauma	1.12	0.73	1.54	0.126	−0.32	2.56
	6-months × childhood trauma	−0.67	0.72	−0.93	0.355	−2.10	0.76
	Baseline × alcohol consumption	−1.40	0.76	−1.85	0.067	−2.90	0.10
	3-months × alcohol consumption	−0.98	1.24	−0.79	0.432	−3.43	1.48
	6-months × alcohol consumption	−1.70	1.23	−1.38	0.170	−4.13	0.73
	Baseline × depression	0.77	0.14	5.50	0.0000003*	0.49	1.04
	3-months × depression	0.01	0.23	0.06	0.952	−0.44	0.47
	6-months × depression	0.53	0.23	2.35	0.021*	0.08	0.98
	Baseline × lifetime trauma	2.06	1.36	1.51	0.134	−0.65	4.76
	3-months × lifetime trauma	0.79	2.24	0.35	0.727	−3.66	5.23
	6-months × lifetime trauma	2.47	2.22	1.11	0.270	−1.95	6.88
	*Baseline BRSK2 CpG4 methylation*						
2A	Baseline × CpG4 (baseline)	0.07	0.11	0.59	0.558	−0.16	0.29
	3-months × CpG4 (baseline)	−0.33	0.12	−2.85	0.005*	−0.56	−0.10
	6-months × CpG4 (baseline)	−0.44	0.12	−3.83	0.0002*	−0.67	−0.21
2B	Baseline × CpG4 (baseline)	−0.08	0.12	−0.70	0.486	−0.31	0.15
	3-months × CpG4 (baseline)	−0.14	0.15	−0.93	0.357	−0.44	0.16
	6-months × CpG4 (baseline)	−0.19	0.15	−1.29	0.201	−0.49	0.10
	Baseline × childhood trauma	1.44	0.51	2.84	0.005*	0.43	2.44
	3-months × childhood trauma	1.20	0.70	1.73	0.086	−0.17	2.58
	6-months × childhood trauma	−0.09	0.69	−0.12	0.902	−1.45	1.28
	Baseline × alcohol consumption	−1.41	0.77	−1.84	0.069	−2.93	0.11
	3-months × alcohol consumption	−1.00	1.23	−0.81	0.421	−3.44	1.45
	6-months × alcohol consumption	−1.60	1.22	−1.32	0.191	−4.02	0.82
	Baseline × depression	0.71	0.14	5.08	0.000002*	0.43	0.98
	3-months × depression	−0.01	0.23	−0.05	0.963	−0.46	0.44
	6-months × depression	0.53	0.22	2.40	0.018*	0.09	0.98
2C	Baseline × CpG4 (baseline)	−0.04	0.12	−0.36	0.720	−0.27	0.19
	3-months × CpG4 (baseline)	−0.12	0.16	−0.75	0.458	−0.42	0.19
	6-months × CpG4 (baseline)	−0.16	0.15	−1.01	0.314	−0.46	0.15
	Baseline × childhood trauma	1.20	0.53	2.24	0.027*	0.14	2.25
	3-months × childhood trauma	1.17	0.77	1.53	0.130	−0.35	2.69
	6-months × childhood trauma	−0.34	0.76	−0.45	0.654	−1.84	1.16
	Baseline × alcohol consumption	−1.47	0.76	−1.93	0.056	−2.99	0.04
	3-months × alcohol consumption	−1.03	1.24	−0.83	0.407	−3.49	1.43
	6-months × alcohol consumption	−1.67	1.22	−1.37	0.175	−4.09	0.75
	Baseline × depression	0.74	0.14	5.28	0.0000008*	0.46	1.02
	3-months × depression	0.00	0.23	0.01	0.996	−0.45	0.46
	6-months × depression	0.57	0.22	2.52	0.013*	0.12	1.01
	Baseline × lifetime trauma	2.11	1.39	1.52	0.132	−0.65	4.88
	3-months × lifetime trauma	0.71	2.27	0.32	0.753	−3.79	5.22
	6-months × lifetime trauma	2.19	2.23	0.98	0.329	−2.24	6.62
	*Baseline BRSK2 CpG5 methylation*						
3A	Baseline × CpG5 (baseline)	0.16	0.10	1.60	0.112	−0.04	0.35
	3-months × CpG5 (baseline)	−0.27	0.10	−2.66	0.009*	−0.47	−0.07
	6-months × CpG5 (baseline)	−0.38	0.10	−3.73	0.0003*	−0.58	−0.18
3B	Baseline × CpG5 (baseline)	−0.06	0.10	−0.57	0.573	−0.25	0.14
	3-months × CpG5 (baseline)	−0.12	0.14	−0.84	0.405	−0.39	0.16
	6-months × CpG5 (baseline)	−0.06	0.14	−0.44	0.657	−0.33	0.21
	Baseline × childhood trauma	1.60	0.50	3.21	0.002*	0.61	2.58
	3-months × childhood trauma	1.33	0.66	2.01	0.047*	0.02	2.65
	6-months × childhood trauma	−0.27	0.66	−0.41	0.683	−1.58	1.04
	Baseline × alcohol consumption	−1.44	0.77	−1.88	0.064	−2.96	0.08
	3-months × alcohol consumption	−1.01	1.23	−0.82	0.412	−3.46	1.43
	6-months × alcohol consumption	−1.72	1.23	−1.41	0.163	−4.16	0.71
	Baseline × depression	0.72	0.14	5.13	0.000002*	0.44	1.00
	3-months × depression	0.00	0.23	0.01	0.991	−0.45	0.45
	6-months × depression	0.48	0.22	2.14	0.035*	0.03	0.93
3C	Baseline × CpG5 (baseline)	−0.03	0.10	−0.29	0.774	−0.23	0.17
	3-months × CpG5 (baseline)	−0.10	0.14	−0.71	0.477	−038	0.18
	6-months × CpG5 (baseline)	−0.03	0.14	−0.22	0.827	−0.31	0.25
	Baseline × childhood trauma	1.34	0.53	2.56	0.012*	0.30	2.39
	3-months × childhood trauma	1.28	0.73	1.74	0.085	−0.18	2.73
	6-months × childhood trauma	−0.58	0.73	−0.80	0.425	−2.03	0.86
	Baseline × alcohol consumption	−1.49	0.76	−1.96	0.054	−3.01	0.02
	3-months × alcohol consumption	−1.04	1.24	−0.84	0.402	−3.50	1.41
	6-months × alcohol consumption	−1.78	1.22	−1.46	0.148	−4.21	0.65
	Baseline × depression	0.75	0.14	5.33	0.0000007*	0.47	1.03
	3-months × depression	0.01	0.23	0.06	0.950	−0.44	0.47
	6-months × depression	0.52	0.23	2.29	0.024*	0.07	0.97
	Baseline × lifetime trauma	2.12	1.39	1.53	0.129	−0.63	4.88
	3-months × lifetime trauma	0.74	2.26	0.33	0.75	−3.74	5.22
	6-months × lifetime trauma	2.52	2.23	1.13	0.262	−1.92	6.96
	*Baseline ADCYAP1 CpG1&2 methylation*						
4A	Baseline × CpG1&2 (baseline)	5.34	1.02	5.26	0.0000009*	3.33	7.36
	3-months × CpG1&2 (baseline)	−1.03	1.12	−0.92	0.360	−3.25	1.19
	6-months × CpG1&2 (baseline)	−3.52	1.18	−2.97	0.004*	−5.86	−1.17
4B	Baseline × CpG1&2 (baseline)	−0.73	0.88	−0.83	0.407	−2.47	1.01
	3-months × CpG1&2 (baseline)	2.75	1.39	1.98	0.050	−0.00	5.50
	6-months × CpG1&2 (baseline)	1.66	1.39	1.20	0.235	−1.10	4.41
	Baseline × childhood trauma	1.84	0.47	3.95	0.0001*	0.92	2.76
	3-months × childhood trauma	1.11	0.59	1.88	0.062	−0.05	2.26
	6-months × childhood trauma	−0.29	0.59	−0.49	0.625	−1.45	0.87
	Baseline × alcohol consumption	−1.48	0.77	−1.93	0.057	−3.01	0.05
	3-months × alcohol consumption	−1.43	1.22	−1.18	0.242	−3.85	0.98
	6-months × alcohol consumption	−1.91	1.21	−1.57	0.120	−4.32	0.51
	Baseline × depression	0.73	0.14	5.29	0.0000008*	0.46	1.01
	3-months × depression	−0.13	0.22	−0.59	0.554	−0.56	0.30
	6-months × depression	0.42	0.22	1.96	0.052	−0.00	0.85
4C	Baseline × CpG1&2 (baseline)	−0.54	0.89	−0.61	0.541	−2.30	1.22
	3-months × CpG1&2 (baseline)	2.76	1.42	1.95	0.055	−0.05	5.57
	6-months × CpG1&2 (baseline)	1.59	1.41	1.13	0.263	−1.22	4.40
	Baseline × childhood trauma	1.87	0.47	4.01	0.0001*	0.95	2.80
	3-months × childhood trauma	1.06	0.59	1.79	0.076	−0.11	2.24
	6-months × childhood trauma	−0.36	0.59	−0.60	0.550	−1.53	0.82
	Baseline × alcohol consumption	−1.44	0.76	−1.88	0.063	−2.96	0.08
	3-months × alcohol consumption	−1.43	1.22	−1.17	0.247	−3.86	1.01
	6-months × alcohol consumption	−1.91	1.22	−1.56	0.121	−4.34	0.52
	Baseline × depression	0.74	0.14	5.33	0.0000006*	0.46	1.01
	3-months × depression	−0.13	0.22	−0.61	0.546	−0.56	0.30
	6-months × depression	0.42	0.22	1.92	0.058	−0.01	0.85
	Baseline × HIV status	−4.51	3.54	−1.27	0.206	−11.55	2.53
	3-months × HIV status	−0.39	5.65	−0.07	0.945	−11.61	10.84
	6-months × HIV status	1.24	5.65	0.22	0.827	−9.99	12.46

*CI* confidence interval, *BRSK2* brain-specific serine/threonine-protein kinase 2, *ADCYAP1* adenylate cyclase activating polypeptide 1.

Increased baseline *ADCYAP1* CpG1&2 methylation was a significant predictor of increased PTSD scores at baseline (*ß* = 5.34, *p* < 0.001) and decreased PTSD scores at 6-months (*ß* = −3.52, *p* = 0.004) post-rape, but the associations were no longer significant when covariates were added to the model.

### Combined sample: longitudinal change in *ADCYAP1* and *BRSK2* methylation levels in relation to longitudinal change in PTSD scores

Table 6 presents the results of the mixed regression models investigating change in *BRSK2* and *ADCYAP1* methylation over time as predictors of change in PTSD symptom scores over time. Decreased *BRSK2* CpG3 (*ß* = −0.39, *p* < 0.001), CpG4 (*ß* = −0.36, *p* = 0.001), and CpG5 (*ß* = −0.32, *p* = 0.001) methylation at 3-months post-rape was associated with increased PTSD scores at 3-months post-rape. Decreased *BRSK2* CpG3 (*ß* = −0.49, *p* < 0.001), CpG4 (*ß* = −0.46, *p* < 0.001), and CpG5 (*ß* = −0.43, *p* < 0.001) methylation at 6-months post-rape was also associated with increased PTSD scores at 6-months post-rape. The relationship between PTSD score at 3-month post-rape and methylation of *BRSK2* CpG3 (*ß* = −0.30, *p* = 0.049) was the only association that remained significant after the addition of covariates to the models.

**Table 6:** Summary statistics of the mixed regression models investigating change in *BRSK2* and *ADCYAP1* methylation over time as predictors of change in posttraumatic stress symptoms scores over time.

Model		*ß*	Std error	*t*	*p*	95% CI	
						Lower	Upper
	*Baseline BRSK2 CpG3 methylation*						
1A	Baseline × CpG3 (baseline)	0.07	0.10	0.71	0.482	−0.13	0.27
	3-months × CpG3 (3-months)	−0.39	0.10	−3.81	0.0002*	−0.60	−0.19
	6-months × CpG3 (6-months)	−0.49	0.10	−4.76	0.000004*	−0.70	−0.29
1B	Baseline × CpG3 (baseline)	−0.16	0.10	−1.60	0.111	−0.37	0.04
	3-months × CpG3 (3-months)	−0.31	0.15	−2.06	0.041*	−0.60	−0.01
	6-months × CpG3 (6-months)	−0.15	0.14	−1.02	0.308	−0.44	0.14
	Baseline × childhood trauma	1.43	0.49	2.93	0.004*	0.47	2.40
	3-months × childhood trauma	1.39	0.66	2.11	0.037*	0.08	2.70
	6-months × childhood trauma	−0.51	0.66	−0.78	0.436	−1.81	0.78
	Baseline × alcohol consumption	−1.31	0.76	−1.72	0.088	−2.81	0.20
	3-months × alcohol consumption	−0.86	1.22	−0.71	0.481	−3.28	1.56
	6-months × alcohol consumption	−1.60	1.24	−1.29	0.199	−4.06	0.86
	Baseline × depression	0.74	0.14	5.34	0.0000006*	0.46	1.01
	3-months × depression	0.06	0.22	0.26	0.793	−0.38	0.50
	6-months × depression	0.50	0.23	2.16	0.034*	0.04	0.97
1C	Baseline × CpG3 (baseline)	−0.15	0.10	−1.42	0.157	−0.35	0.06
	3-months × CpG3 (3-months)	−0.30	0.15	−1.99	0.049*	−0.60	−0.00
	6-months × CpG3 (6-months)	−0.12	0.15	−0.80	0.423	−0.41	0.17
	Baseline × childhood trauma	1.18	0.52	2.89	0.024*	0.16	2.21
	3-months × childhood trauma	1.34	0.73	1.84	0.068	−0.10	2.78
	6-months × childhood trauma	−0.82	0.73	−1.12	0.263	−2.25	0.62
	Baseline × alcohol consumption	−1.35	0.75	−1.79	0.077	−2.84	0.15
	3-months × alcohol consumption	−0.88	1.23	−0.71	0.477	−3.31	1.56
	6-months × alcohol consumption	−1.66	1.24	−1.34	0.184	−4.11	0.80
	Baseline × depression	0.77	0.14	5.55	0.0000002*	0.49	1.05
	3-months × depression	0.07	0.23	0.31	0.761	−0.38	0.52
	6-months × depression	0.53	0.23	2.27	0.026*	0.07	1.00
	Baseline × lifetime trauma	2.02	1.36	1.48	0.141	−0.68	4.72
	3-months × lifetime trauma	0.57	2.22	0.26	0.797	−3.84	4.98
	6-months × lifetime trauma	2.28	2.27	1.01	0.317	−2.23	6.79
	*Baseline BRSK2 CpG4 methylation*						
2A	Baseline × CpG4 (baseline)	0.03	0.11	0.32	0.749	−0.17	0.24
	3-months × CpG4 (3-months)	−0.36	0.11	−3.40	0.001*	−0.57	−0.15
	6-months × CpG4 (6-months)	−0.46	0.11	−4.92	0.00003*	−0.68	−0.25
2B	Baseline × CpG4 (baseline)	−0.13	0.11	−1.21	0.230	−0.35	0.84
	3-months × CpG4 (3-months)	−0.30	0.15	−2.04	0.043*	−0.59	−0.01
	6-months × CpG4 (6-months)	−0.22	0.15	−1.49	0.138	−0.51	0.07
	Baseline × childhood trauma	1.36	0.49	2.75	0.007*	0.38	2.34
	3-months × childhood trauma	1.48	0.69	2.14	0.034*	0.11	2.84
	6-months × childhood trauma	−0.28	0.69	−0.40	0.689	−1.64	1.09
	Baseline × alcohol consumption	−1.35	0.76	−1.77	0.080	−2.87	0.16
	3-months × alcohol consumption	−0.85	1.22	−0.70	0.488	−3.27	1.57
	6-months × alcohol consumption	−1.59	1.22	−1.30	0.197	−4.02	0.84
	Baseline × depression	0.72	0.14	5.15	0.000001*	0.44	0.99
	3-months × depression	0.07	0.22	0.32	0.751	−0.37	0.52
	6-months × depression	0.55	0.23	2.38	0.020*	0.098	1.01
2C	Baseline × CpG4 (baseline)	−0.10	0.11	−0.88	0.379	−0.32	0.12
	3-months × CpG4 (3-months)	−0.28	0.15	−1.88	0.062	−0.58	0.14
	6-months × CpG4 (6-months)	−0.19	0.15	−1.25	0.215	−0.48	0.11
	Baseline × childhood trauma	1.12	0.52	2.14	0.035*	0.08	2.15
	3-months × childhood trauma	1.46	0.76	1.93	0.056	−0.04	2.96
	6-months × childhood trauma	−0.53	0.76	−0.70	0.488	−2.03	0.97
	Baseline × alcohol consumption	−1.41	0.76	−1.85	0.067	−2.92	0.10
	3-months × alcohol consumption	−0.87	1.23	−0.71	0.478	−3.31	1.56
	6-months × alcohol consumption	−1.64	1.22	−1.34	0.183	−4.08	0.79
	Baseline × depression	0.75	0.14	5.34	0.0000006*	0.47	1.02
	3-months × depression	0.79	0.23	0.35	0.730	−0.37	0.53
	6-months × depression	0.58	0.23	2.48	0.015*	0.12	1.04
	Baseline × lifetime trauma	2.00	1.39	1.44	0.154	−0.76	4.75
	3-months × lifetime trauma	0.49	2.22	0.22	0.826	−3.93	4.91
	6-months × lifetime trauma	2.12	2.25	0.95	0.345	−2.33	6.59
	*Baseline BRSK2 CpG5 methylation*						
3A	Baseline × CpG5 (baseline)	0.10	0.09	1.07	0.285	−0.08	0.28
	3-months × CpG5 (3-months)	−0.32	0.09	−3.40	0.001*	−0.51	−0.14
	6-months × CpG5 (6-months)	−0.43	0.10	−4.42	0.00002*	−0.62	−0.24
3B	Baseline × CpG5 (baseline)	−0.11	0.10	−1.10	0.275	−0.30	0.09
	3-months × CpG5 (3-months)	−0.25	0.14	−1.81	0.073	−0.53	0.02
	6-months × CpG5 (6-months)	−0.15	0.13	−1.11	0.269	−0.42	0.12
	Baseline × childhood trauma	1.43	0.49	2.91	0.004*	0.46	2.40
	3-months × childhood trauma	1.46	0.67	2.17	0.032*	0.13	2.78
	6-months × childhood trauma	−0.35	0.66	−0.54	0.593	−1.65	0.95
	Baseline × alcohol consumption	−1.37	0.76	−1.79	0.076	−2.88	0.15
	3-months × alcohol consumption	−0.98	1.23	−0.80	0.429	−3.41	1.46
	6-months × alcohol consumption	−1.62	1.23	−1.32	0.190	−4.06	0.82
	Baseline × depression	0.72	0.14	5.18	0.000001*	0.45	1.00
	3-months × depression	0.05	0.22	0.21	0.834	−0.40	0.49
	6-months × depression	0.52	0.23	2.26	0.027*	0.06	0.98
3C	Baseline × CpG5 (baseline)	−0.08	0.10	−0.81	0.417	−0.27	0.11
	3-months × CpG5 (3-months)	−0.24	0.14	−1.68	0.095	−0.51	0.04
	6-months × CpG5 (6-months)	−0.12	0.14	−0.86	0.390	−0.39	0.15
	Baseline × childhood trauma	1.19	0.52	2.28	0.024*	0.16	2.22
	3-months × childhood trauma	1.41	0.74	1.91	0.058	−0.05	2.87
	6-months × childhood trauma	−0.62	0.73	−0.86	0.391	−2.06	0.81
	Baseline × alcohol consumption	−1.42	0.76	−1.87	0.065	−2.93	0.88
	3-months × alcohol consumption	−0.99	1.23	−0.81	0.423	−3.45	1.46
	6-months × alcohol consumption	−1.68	1.23	−1.37	0.175	−4.12	0.76
	Baseline × depression	0.75	0.14	5.37	0.0000005*	0.48	1.03
	3-months × depression	0.06	0.23	0.26	0.800	−0.40	0.51
	6-months × depression	0.55	0.23	2.36	0.020*	0.09	1.01
	Baseline × lifetime trauma	2.03	1.38	1.47	0.145	−0.71	4.77
	3-months × lifetime trauma	0.62	2.24	0.28	0.781	−3.82	5.06
	6-months × lifetime trauma	2.23	2.26	0.98	0.328	−2.27	6.72
	*Baseline ADCYAP1 CpG1&2 methylation*						
4A	Baseline × CpG5 (baseline)	4.67	0.92	5.10	0.000001*	2.86	6.49
	3-months × CpG5 (3-months)	−2.61	0.80	−3.26	0.001*	−4.20	−1.02
	6-months × CpG5 (6-months)	−5.01	1.12	−4.48	0.00002*	−7.23	−2.80
4B	Baseline × CpG5 (baseline)	−1.32	0.83	−1.16	0.113	−2.97	0.32
	3-months × CpG5 (3-months)	−1.46	0.92	−1.59	0.116	−3.29	0.37
	6-months × CpG5 (6-months)	−0.44	1.28	−0.34	0.734	−2.97	2.10
	Baseline × childhood trauma	1.77	0.47	3.76	0.0003*	0.84	2.70
	3-months × childhood trauma	1.44	0.61	2.37	0.019*	0.24	2.63
	6-months × childhood trauma	−0.19	0.60	−0.31	0.757	−1.37	1.00
	Baseline × alcohol consumption	−1.41	0.77	−1.84	0.068	−2.93	0.11
	3-months × alcohol consumption	−1.27	1.24	−1.02	0.309	−3.73	1.19
	6-months × alcohol consumption	−1.74	1.22	−1.42	0.158	−4.17	0.69
	Baseline × depression	0.74	0.14	5.35	0.0000006*	0.46	1.01
	3-months × depression	−0.03	0.22	−0.16	0.876	−0.46	0.40
	6-months × depression	0.46	0.21	2.14	0.035*	0.03	0.88
4C	Baseline × CpG5 (baseline)	−1.12	0.83	−1.35	0.182	−2.78	0.54
	3-months × CpG5 (3-months)	−1.54	0.93	−1.66	0.100	−3.38	0.30
	6-months × CpG5 (6-months)	−0.48	1.29	−0.37	0.712	−3.03	2.08
	Baseline × childhood trauma	1.80	0.47	3.84	0.0002*	0.87	2.73
	3-months × childhood trauma	1.37	0.61	2.23	0.027*	0.16	2.58
	6-months × childhood trauma	−0.26	0.61	−0.42	0.675	−1.46	0.95
	Baseline × alcohol consumption	−1.38	0.76	−1.81	0.074	−2.90	0.17
	3-months × alcohol consumption	−1.29	1.25	−1.04	0.302	−3.77	1.18
	6-months × alcohol consumption	−1.76	1.23	−1.43	0.157	−4.21	0.69
	Baseline × depression	0.74	0.14	5.40	0.0000005*	0.47	1.01
	3-months × depression	−0.04	0.22	−0.20	0.844	−0.48	0.39
	6-months × depression	0.45	0.22	2.08	0.040*	0.02	0.88
	Baseline × HIV status	−4.28	3.53	−1.21	0.229	−11.30	2.74
	3-months × HIV status	2.16	5.78	0.37	0.709	−9.32	13.65
	6-months × HIV status	1.86	5.60	0.33	0.740	−9.27	12.99

*CI* confidence interval, *BRSK2* brain-specific serine/threonine-protein kinase 2, *ADCYAP1* adenylate cyclase activating polypeptide 1.

Increased baseline *ADCYAP1* CpG1&2 methylation was associated with increased PTSD scores at baseline (*ß* = 4.67, *p* < 0.001), while decreased *ADCYAP1* CpG1&2 methylation at 3-months (*ß* = −2.61, *p* = 0.001) and 6-months (*ß* = −5.01, *p* < 0.001) was associated with increased PTSD scores at 3-months and 6-months post-rape. The associations were no longer significant when covariates were added to the model.

## Discussion

In this study, we identified one DMP (cg01700569) and thirty-four DMRs associated with PTSD at 3-months post-rape on an epigenome-wide level. The gene closest to the aforementioned DMP is *SLC16A9*. Although investigating this DMP further may have been of value, little is known about it in the context of mental health. The site (cg01700569) is located in an intergenic region, which further complicates the interpretation of the clinical significance of the finding.

We investigated two DMRs in the *BRSK2* and *ADCYAP1* genes further. We were able to validate, but not replicate, the *BRSK2* CpG5 finding, confirming decreased *BRSK2* methylation in rape-exposed participants with PTSD at 3-months post-rape, compared to those without PTSD. We also found that decreased baseline *BRSK2* CpG3, CpG4, and CpG5 methylation was associated with increased PTSD scores at 3-months and 6-months post-rape. Decreased *BRSK2* methylation at 3-months and 6-months post-rape was associated with increased PTSD scores at the same time points. However, the associations between decreased *BRSK2* CpG3 methylation at 3-months post-rape and increased PTSD scores at 3-months post-rape were the only ones that remained significant after childhood trauma, alcohol consumption, depression, and lifetime trauma were added as covariates to the models.

We were unable to validate or replicate our *ADCYAP1* CpG1&2 findings. We found that decreased baseline *ADCYAP1* CpG1&2 methylation was associated with increased PTSD scores at 6-months post-rape. Decreased *ADCYAP1* methylation at 3-and 6-months post-rape was also associated with increased PTSD scores at the same time points, while decreased baseline *ADCYAP1* CpG1&2 methylation was associated with decreased PTSD scores at baseline. The findings did not remain significant after PTSD covariates were added to the models.

Decreased methylation of the *BRSK2* paralog, *BRSK1* [68], has been associated with a PTSD diagnosis in a prior EWAS [14]. *BRSK1* and *BRSK2* share a 68% overlap in genetic sequence, both are highly expressed in the brain, and decreased expression of both has been linked to disorganized presynaptic vesicle formation, uncoordinated release and reuptake of neurotransmitters, altered axonal development, and abnormal neuronal polarization in animal studies [68–73]. In human studies, a *BRSK2* polymorphism (rs1881509) has been associated with heroin dependence [69], and functional variants of *BRSK2* have been associated with autism spectrum disorder, cognitive impairment, intellectual disability, and speech delays [74, 75].

*BRSK1* and *BRSK2* are expressed most strongly in the cerebellum and the hippocampus [69]. The hippocampus is closely linked to PTSD since it is involved in memory consolidation [76]. When memories are not consolidated into autobiographical memory networks, they may involuntarily resurface (e.g., flashbacks, intrusions, nightmares, and dissociation) and activate the limbic system, which induces the fight-or-flight response [77]. Differential methylation and expression of *BRSK2* may also alter the expression of neurotransmitters previously found to be associated with PTSD (norepinephrine, epinephrine, dopamine, and serotonin) through altered presynaptic vesicle and synaptic cleft development [78, 79].

In addition to their functions in the brain, *BRSK1* and *BRSK2* have been linked to metabolic processes and glucose homeostasis [80, 81]. Animal studies have found increased expression of *BRSK1* and *BRSK2* in pancreatic cells and knockdown of *BRSK2* resulted in a significant increase in serum insulin levels [80, 81]. In a human study, *BRSK2* was found to be highly expressed in human pancreatic insulin-producing B cells, and activation of *BRSK2* was linked to reduced insulin secretion [81]. Moreover an EWAS found that participants with type 1 diabetes and neuropathy showed decreased methylation at four CpG sites in the *BRSK2* gene compared to participants with type 1 diabetes without neuropathy [82].

The *BRSK2* CpG sites investigated in this study were located in intron 4 of the gene. The function of methylation in gene bodies is not well established, but methylation is abundant in these regions and is generally positively correlated with expression [83]. Assuming the latter, we can hypothesize that decreased methylation of *BRSK2* may contribute to adverse neuronal development, neuronal maintenance, and dysregulated blood glucose levels which may explain the increased risk for diabetes and cardiovascular disease observed in prior PTSD studies [84, 85]. The relationship between *BRSK2* methylation and adverse neuronal development and maintenance is further supported by prior findings of a high correlation between *BRSK2* blood methylation and methylation in brain tissue [59].

We investigated *ADCYAP1* further, since its protein product, PACAP, has been identified as a master regulator of the HPA-axis and the stress response [86]. The highest concentration of PACAP in the brain is found in the hypothalamus [87]. PACAP binding in the hypothalamus triggers the release of corticotrophin-releasing hormone (CRH) and signals the activation of the stress response [86]. In the adrenal medulla, PACAP binding to PAC1R (product of *ADCYAP1R1*) stimulates the release of catecholamines as part of the sympathetic nervous system (SNS) [88]. PACAP binding to PACR1 in preganglionic neurons triggers the release of phenylethanolamine-N-methyltransferase (PNMT) and tyrosine hydroxylase (TH) in effector organs of the SNS. PNMT and TH are catecholamine-synthesizing enzymes and sustain the release of catecholamines in the effector organs during the stress response [88].

Researchers investigating PACAP/*ADCYAP1* and PACR1/*ADCYAP1R1* in relation to PTSD in a predominantly African− American sample with a mixture of trauma types found that, in women more than men, increased PACAP blood levels were associated with increased PTSD symptom severity and an increased acoustic startle reflex response [64, 89]. They also found that women carrying the *ADCYAP1R1* rs2267735 CC genotype showed decreased *ADCYAP1R1* mRNA expression, increased PTSD symptom severity, increased dark-enhanced startle response, and increased amygdala and hippocampal activity in response to viewing threatening face stimuli [64–66, 89]. In both men and women, increased methylation of *ADCYAP1R1* was associated with decreased cortical mRNA expression and increased PTSD symptom severity [64, 90]. However, the functional effects of *ADCYAP1* and *ADCYAP1R1* seem to be more pronounced in women compared to men [64–66], due to the presence of several estrogen response elements (EREs) in the *ADCYAP1R1* promoter. The CC genotype of rs2267735 has been associated with decreased binding of estrogen receptor alpha to the EREs and decreased expression of *ADCYAP1R1* [91]. The role of estrogen in *ADCYAP1R1* and HPA-axis activity may in part explain why women have an increased risk of PTSD compared to men [35, 92].

The two *ADCYAP1* CpG sites investigated in this study are located in a CpG island spanning the 1st intron of the gene. Methylation in CpG islands and in the 1st intron of a gene is generally associated with decreased expression of the gene [93–95]. Our longitudinal findings, therefore, correspond with prior findings since decreased methylation of *ADCYAP1* is likely to result in increased expression of PACAP and increased PTSD symptom severity [65, 66, 91, 96]. Decreased PACAP is also likely to result in decreased binding to PAC1 and reduced activation of the HPA-axis [86, 88].

Based on prior findings, *ADCYAP1* CpG1&2 DNA methylation in blood was not significantly correlated with DNA methylation at the same sites in brain tissue [59]. However, the brain regions investigated did not specifically focus on the region where PACAP is most abundantly expressed i.e., the paraventricular nucleus of the hypothalamus, and investigating blood-brain methylation in this region may show different results [37]. It is also likely that the expression of PACAP in the endocrine system has a more profound effect on the regulation of the HPA-axis compared to PACAP expression in the brain [37].

We found that, before correction for multiple testing, CpG sites in *HTR3A* [67]*, AHRR* [22]*, DUSP22* [15], and *TPR* [13] were associated with PTSD. The results from our study are in line with recent results from the largest EWAS meta-analysis of PTSD published to date [22], where *AHRR* cg05575921 and cg26703534 were found to exhibit reduced DNA methylation in individuals with PTSD. Decreased *AHRR* methylation at these CpG sites was also associated with decreased kynurenine and kynurenic acid in the same study [22]. Kynurenine ligand binding to aryl hydrocarbon receptors has been associated with the expression of anti-inflammatory genes which may be disrupted by decreased methylation of *AHRR* [22, 25]. This may result in increased levels of proinflammatory cytokines and the low-grade inflammatory state often observed in PTSD [97, 98]. Upregulation in kynurenine to restore the imbalance between pro-inflammatory and anti-inflammatory cytokines may also result in reduced levels of serotonin since both kynurenine and serotonin are synthesized from tryptophan [99]. A strong link between decreased *AHRR* methylation and smoking has also been reported in previous studies although some studies have reported a significant relationship between *AHRR* methylation and PTSD independent of the effect of smoking [22, 100–102].

Our findings should be interpreted in light of a number of limitations. First, the EWAS was conducted in a small sample of participants. However, the study was well designed to limit variation between groups. Second, we used DNA extracted from whole blood to measure methylation levels while differential methylation in brain tissue is a more direct approximation of PTSD pathophysiology. However, based on prior findings, we observed that blood-brain methylation was highly correlated at the *BRSK2* CpG sites investigated in this study, but not at the *ADCYAP1* CpG sites. Blood is easily accessible and blood biomarkers of PTSD risk may be a more pragmatic approach for personalized treatment of individuals at high risk of developing PTSD following trauma exposure [103]. Third, we may have overcorrected for confounding variables in the EWAS given that SVA was used along with the inclusion of cell-type composition as a covariate in the final models. Fourth, we did not investigate methylation quantitative trait loci (meQTL) located in the *BRSK2* and *ADCYAP1* genes. SNPs located in these genes may predict or mediate the methylation profiles observed in relation to PTSD status and symptom scores. Finally, DNA methylation in relation to gene expression and/or protein levels was not objectively measured and conclusions related to the functional effects of methylation are speculative.

The study has many strengths. First, all participants were rape-exposed women from similar sociodemographic backgrounds and from the same ethnicity group thus making the sample relatively homogenous. Second, the analyses were robust with a variety of confounding factors controlled for i.e., participants who were pregnant/lactating were excluded, none of the participants were on psychotropic medication and participants were of similar age. Baseline measures of age, HIV status, BMI, smoking, childhood trauma, lifetime trauma, alcohol use, and depression were controlled for by matching participants on these variables in the cross-sectional EWAS and including these factors as covariates/confounders in the longitudinal analyses. Third, we attempted to expand the findings of the EWAS by including longitudinal data which allowed us to investigate changes in methylation in relation to change in PTSD symptom scores over time. Fourth, investigating the agreement between the results obtained from the two different laboratory methods used (Illumina EPIC array and EpiTYPER) also allowed identification of potential bias/variation introduced by the different procedures involved in each method.

In summary, this study provides evidence that differential methylation of genes related to neurogenesis/development, glucose homeostasis, and HPA-axis regulation may be involved in PTSD development following rape. Our findings are supported by previous research implicating *ADCYAP1/ADCYAP1R1* (especially in women) and *BRSK1/BRSK2* in the development of PTSD. However, replication of these findings is required to determine whether the differentially methylated regions identified in this study are consistently linked to the development of PTSD.

## Supplementary information

Supplementary Material
