# Supplementary material for: Genome-wide differentially methylated genes associated with posttraumatic stress disorder and longitudinal change in methylation in rape survivors
Source: Transl Psychiatry. 2021 Nov 19;11:594. doi: 10.1038/s41398-021-01608-z (PMC8604994; doi:10.1038/s41398-021-01608-z)
Supplement: Supplementary file 1 — Supplementary Material [file 41398_2021_1608_MOESM1_ESM.docx]

**SUPPLEMENTARY MATERIAL**

**METHODS**

**Parent study**

A subset of participants (n=97), recruited from a longitudinal cohort study investigating the impact of rape on women’s health and their use of health services in South Africa (n = 852), was used in this study. The study is also known as the Rape Impact Cohort Evaluation (RICE) study ^1^. Female survivors of rape were recruited from rape centres in and around the city of Durban located in the KwaZulu Natal province of South Africa. The rape centres provide comprehensive emergency care, including access to police, counselling, and medical and forensic care.

**Clinical measures**

***The Mini International Neuropsychiatric Interview***

The Mini International Neuropsychiatric Interview (MINI) is a structured psychiatric interview that screens for 16 DSM-IV psychiatric disorders including mood disorders, anxiety disorders, alcohol and drug dependence, psychosis, eating disorders and personality disorders ^2^. The MINI was used at baseline to screen for PTSD based on prior traumas other than the rape. The MINI has shown good reliability and validity in various settings ^3,4^.

***Davidson Trauma Scale***

The Davidson Trauma Scale (DTS) is a self-report questionnaire used to assess seventeen PTSD symptoms ^5^. The DTS was administered at all timepoints and responses were measured on a 5-point Likert scale for symptom frequency (ranging from 0 ‘not at all’ to 4 ‘every day’) and symptom severity (ranging from 0 ‘not at all distressing’ to 4 ‘extremely distressing’). The symptom frequency and severity scores were added together to produce a PTSD total score ranging between 0 and 136. A total score of forty or more is considered indicative of PTSD. Previous findings indicated that the DTS was excellent at discriminating between participants with and without PTSD at a cut-point of 40 ^5,6^. The DTS showed excellent reliability in this study at each timepoint with a Cronbach alpha sore of .92 at baseline, .91 at 3-months and .93 at 6-months post-rape.

***Childhood Trauma Questionnaire Short Form***

A modified version of the Childhood Trauma Questionnaire Short Form (CTQ-SF) was used to measure exposure to childhood trauma before the age of eighteen years ^7,8^. The fourteen items measuring childhood trauma centres around sexual abuse, physical abuse, emotional abuse, parental neglect and domestic violence. Responses were measured on a 4-point Likert scale ranging from 1 ‘never’ to 4 ‘very often’. The CTQ-SF has shown excellent validity in previous studies ^9,10^ and showed acceptable reliability in this study with a Cronbach alpha score of .75 at baseline.

***Life Events Checklist***

A modified version of the Life Events Checklist (LEC) was used to measure lifetime exposure to different trauma types at the baseline visit ^11,12^. The modified version of the LEC measures direct exposure to nine trauma types using a dichotomous ‘yes/no’ response. The trauma types measured were imprisonment, civil unrest/war, serious injury, being close to death, murder of a family member or friend, unnatural death of a family member or friend, murder of a stranger/s, robbed at gunpoint or knifepoint, and kidnapping. The number of yes responses were added together to yield a total score ranging from 0 to 9 and indicating the trauma load or number of traumas a participant was exposed to during their lifetime.

***Alcohol Use Disorders Identification Test (AUDIT)***

The original Alcohol Use Disorders Identification Test (AUDIT) is a 10-item, self-report questionnaire and responses are recorded on a five-point Likert scale using different response options relevant to the different items ^13^. The AUDIT-C is a sub-scale of the original AUDIT and is used to measure alcohol consumption ^14^. A score of 3 or more on the AUDIT-C indicates hazardous drinking in women. The AUDIT was developed by the World Health Organisation (WHO) and has shown good reliability and validity in various settings and cultures ^15–21^. The AUDIT-C showed good reliability in this study at each timepoint with a Cronbach alpha sore of .83 at baseline, .86 at 3-months post-rape and .83 at 6-months post-rape.

***Center for Epidemiologic Studies Depression Scale (CES-D)***

The Center for Epidemiologic Studies Depression Scale (CESD) is a 20-item, self-report questionnaire used to screen for current depression in accordance with DSM-IV criteria ^22^. The CESD was completed at all time points. Responses were recorded on a 4-point Likert scale ranging from 0 ‘rarely or none of the time’ to 3 ‘most or all of the time’. The CESD has shown good reliability and validity in various cross-cultural samples and in clinic and community settings ^23–32^. The CES-D showed good reliability in this study at each timepoint with a Cronbach alpha sore of .89 at baseline, .88 at 3-months and .89 at 6-months post-rape.

**Procedure**

***Epigenome-wide association study***

The Illumina MethylationEPIC BeadChip array was used to investigated genome-wide differences in methylation between participants with and without PTSD (Illumina, California, United States). The array interrogates over 850 000 sites at single-nucleotide resolution covering 99% of RefSeq genes and including CpG islands, shores and shelves. Results obtained from the MethylationEPIC BeadChip are highly reproducible (98%) with a less than 1% false positive rate reported Illumina ^33,34^.

DNA was extracted from peripheral blood samples using the Gentra Puregene DNA extraction kit (Qiagen, Germany) and quantified by fluorimetry, using the PicoGreen dsDNA quantitation reagent (ThermoFisher Scientific, Massachusetts, United States). Sample concentrations were normalised to 50 ng/μl and shipped on dry ice to the Epigenome Centre at the University of South California (USC) in Los Angeles, United States. The DNA samples were bisulfite-converted using the Zymo EZ DNA Methylation Kit (Zymo Research, California, United States) and assayed using the Illumina MethylationEPIC BeadChip (Illumina, California, United States). The samples were randomly assigned to one of six arrays (8 samples per array) to reduce technical bias.

***Quality control***

Internal control probes, negative control probes and a minimum detection rate of three beads per CpG site were used to assess the performance of the MethylationEPIC array. The arrays passed all of the sample processing quality control measures assessing success of the bisulfite conversion, hybridization, staining, extension, specificity and target removal, using the internal control probes^35^. There were 29 936 probes that did not pass the quality control measures assessing whether (1) the methylated/unmethylated intensities are likely true intensities and not a result of background noise ^35^, and (2) whether a signal was detected from at least three beads per probe.

***Functional normalisation and batch effects***

Functional normalisation was applied in an effort to reduce the technical bias associated with batch effects, using the information obtained from the 42 control probes on the MethylationEPIC array ^36^. This method removes the variance associated with technical variation without compromising the variance associated with methylation differences between PTSD cases and controls since none of the control probes are designed to detect a biological signal ^36^. Five principal components (PCs) were found to explain most of the technical variance in the sample and were regressed against the batch variables i.e. array and row position, before and after functional normalisation ^37^ (see Supplementary Figure 1).

***Batch effects before functional normalisation***

The PC for each control probe was regressed against all levels of each batch variable (see Supplementary Figures 2a-2b and Supplementary Figures 3a-3e) to determine the extent to which control variation accounted for batch variation. There were six slide batch variables representing the six arrays used in the epigenome-wide association study (EWAS; slide 201904850009, slide 201959740048, slide 201959750230, slide 201990430029, slide 201985320017, slide 201995320078) and eight row batch variables representing the sentrix row on an array to which a sample was assigned (sentrix row 01 – sentrix row 08). The significant findings resulting from ANOVAS and post-hoc t-tests comparing PC values between batch variables are presented in Supplementary Table 1.

Principal components analysis was then applied to the normalized DNA methylation matrix. Each top PC was regressed against each level of each batch variable following functional normalisation (see Supplementary Figures 4a-4b and Supplementary Figures 5a-5e) to evaluate the extent of technical variation remaining in the normalized data. None of the analysis of variance (ANOVA) tests comparing PC values with slide and row batch variables were statistically significant, but post-hoc t-test comparisons did reveal some significant findings between PC values and slide/row numbers. The results of the significant t-tests are presented in Supplementary Table 2.

***Confounding variables***

We investigated the impact of potential confounding variables (cell type composition, age, body mass index (BMI), smoking, HIV status, medication use, childhood trauma exposure, lifetime trauma exposure, alcohol use and depression) on methylation associated with PTSD status using functions in the R package Combat ^38^. Age, body mass index (BMI), smoking, HIV status, medication use, childhood trauma exposure, lifetime trauma exposure, alcohol use and depression were not significant covariates, but cell type composition was.

***Surrogate variable analysis***

Since all batch effects were not fully removed using functional normalisation, we also performed surrogate variable analysis (SVA) ^39^. In addition to addressing the variance explained by batch effects and other technical sources, SVA can also account for unknown unwanted variance caused by biological differences in the samples which are unrelated to the phenotype of interest e.g. covariates such as cell type composition, age, BMI, smoking, HIV status, medication use, childhood trauma exposure, lifetime trauma exposure, alcohol use and depression ^39,40^. The resulting surrogate variables were included as covariates in the EWAS models. Cell type composition was also included as confounding variables in the EWAS models given that they were identified as significant confounders in the analysis conducted using Combat.

***Blood cell type composition***

Blood cell type composition is another source of bias often introduced when using whole blood to investigate methylation profiles. Whole blood contains several different cell types and each type has its own methylation profile ^41^. SVA has been shown to be successful in removing the unwanted variance introduced by cell-type distribution, but as a precautionary measure we also estimated cell type composition using the Houseman algorithm and the publicly available blood cell type reference dataset GSE35069 (available on the Gene Expression Omnibus) ^42^ and included cell type compositions for B lymphocytes, CD4 T-cells, CD8 T-cells, eosinophils, monocytes, neutrophils and natural killer cells in the final analysis (see Supplementary Figure 6 for estimated cellular composition).

***Differentially methylated regions (DMRs)***

Differentially methylated regions (DMRs) were identified using the *dmrff* R package ^43^ (for ). DMR analysis improves statistical power given that the EWAS CpG sites can be subdivided into clusters and associations assessed as a group rather than individually. There are often strong dependencies between CpG sites, especially those clustering around the same gene or those in close genomic proximity to each other ^43,44^. The default setting applied by the *dmrff* package for qualification as a DMR was used in this study with the exception of the distance between CpG sites. The default value in the *dmrff* package is 500 bp apart, but we applied a more stringent criteria of 100 bp apart since CpG sites are more likely to co-methylate if they are in closer proximity to each other ^45,46^.

***Annotation***

Coordinates resulting from the differentially methylated positions (DMP) and DMR analyses were annotated using the MethylationEPIC_v-1-0_B4 manifest. All genomic coordinates reported in this study is in reference to the Hg19/GRCh37 human genome assembly. The location of a CpG site was determined using the ‘University of California Santa Cruz (UCSC) reference gene group’. TSS200 indicates that the CpG site is located 0 to 200 bp upstream of the transcription start site (TSS), TSS1500 indicates that the CpG site is located 200-1500bp upstream of the TSS, 5’UTR indicates that the CpG site is located within the 5’ untranslated region between the TSS and ATG (the protein coding start site), body indicates that the CpG site is located between the ATG and the stop codon (protein coding end site), irrespective of introns, exons, TSS or promoters in the region, 3’UTR indicates that the CpG site is located between the stop codon and the polyadenylation signal.

***Validation, replication and longitudinal analysis***

Sample concentrations were normalised to 50 ng/ul and shipped on ice to Inqaba Biotec in Pretoria, South Africa for bisulfite conversion and analysis using EpiTYPER Sequenom MassARRAY (Agena Bioscience, California, United States) technology. The method involved: (1) PCR primer design using Sequenom Epidesigner software ([www.epidesigner.com](http://www.epidesigner.com)); (2) bisulfite conversion using the Zymo EZ DNA methylation kit (Zymo Research, California, United States); (3) PCR amplification of the target region using t7-promoter tagged primers; (4) RNA transcription and base-specific RNA cleavage; (5) quantification of DNA methylation based on the size and mass of RNA fragments using the SpectroCHIP Array, matrix-assisted laser desorption ionization time-of-flight (MALDI-TOF) mass spectrometry and EpiTYPER software. The samples were randomly assigned to one of three plates (96 samples per plate). The EpiTYPER procedure is highly reproducible and has been found to detect methylation levels as low as 5% ^47,48^.

The primer designs used in the EpiTYPER analysis for *BRSK2* and *ADCYAP1* are provided below.

*BRSK2*

forward primer, 5’-aggaagagagGAAGGAGTTTGTTAGGGTGTAGGAG-3’ and reverse primer, 5’- cagtaatacgactcactatagggagaaggctAAACAAAACCAATCTCTACCTTAAA-3’

*ADCYAP1*

forward primer, 5’- aggaagagagGGTTTTTTGAGTTGAATAGTATTTGG-3’ and reverse primer, 5’- cagtaatacgactcactatagggagaaggctTCCTACCTAACAACTCTCCTAACAA-3’

**RESULTS**

Supplementary results related to the discovery sample, validation sample and replication sample are presented in Supplementary Tables 3-11 and Supplementary Figures 7-9. Findings related to prior EWASs and candidate gene studies (in relation to the current EWAS) are presented in Supplementary Tables 12 and 13. Supplementary results related to the longitudinal findings in the combined sample are presented in Supplementary Table 14.

**Blood-brain methylation comparison**

Co-variation in methylation levels between blood and brain tissue was explored using the online Blood Brain DNA Methylation Comparison Tool ^51^. Blood methylation of the *BRSK2* CpG sites were highly correlated with methylation in the prefrontal cortex, superior temporal gyrus and the cerebellum (see Supplementary Figures 10a-10d). The blood-brain methylation correlation for *BRSK2* CpG5 is not provided since the correlations provided by the Blood Brain DNA Methylation Comparison Tool is based on the Illumina 450K array and CpG5 was not included on this array. There were no significant correlations between blood and brain methylation for the *ADCYAP1* CpG sites (see Supplementary Figures 11a and 11b).


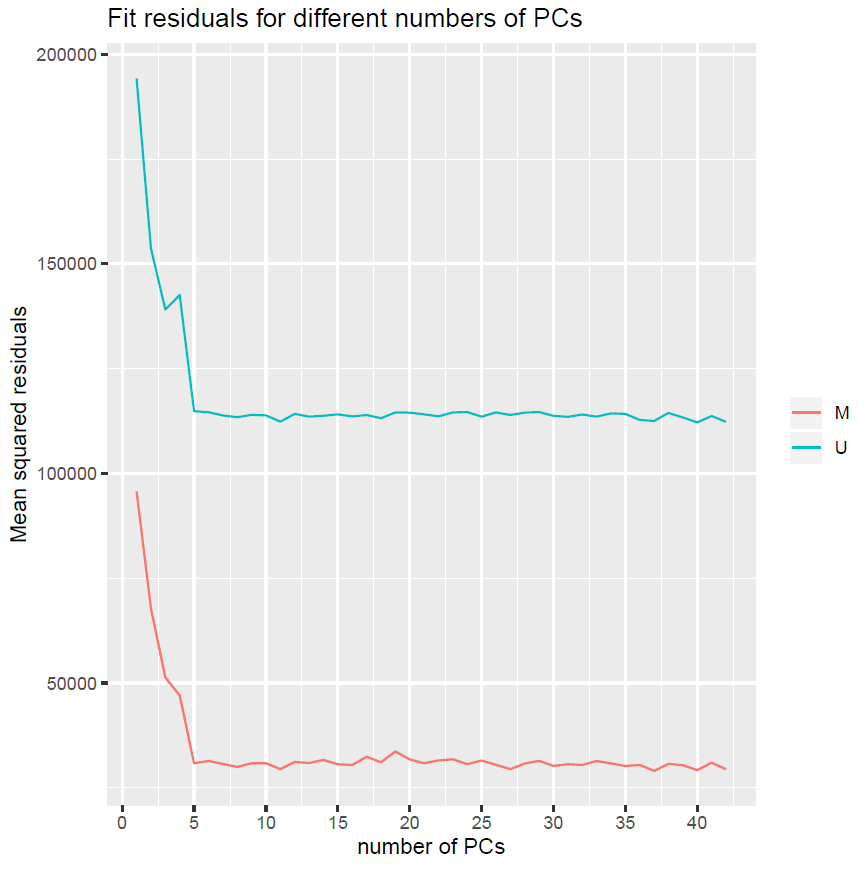
*Supplementary Figure 1:* Estimating the number of principal components (PCs) to regress against batch variables. The first five PCs explained most of the technical variance observed from the methylated (M) and unmethylated (U) intensities. Variance explained was estimated using cross-validation. The *x*-axis represents the number of PCs and the *y*-axis represents the mean squared residuals/variance.


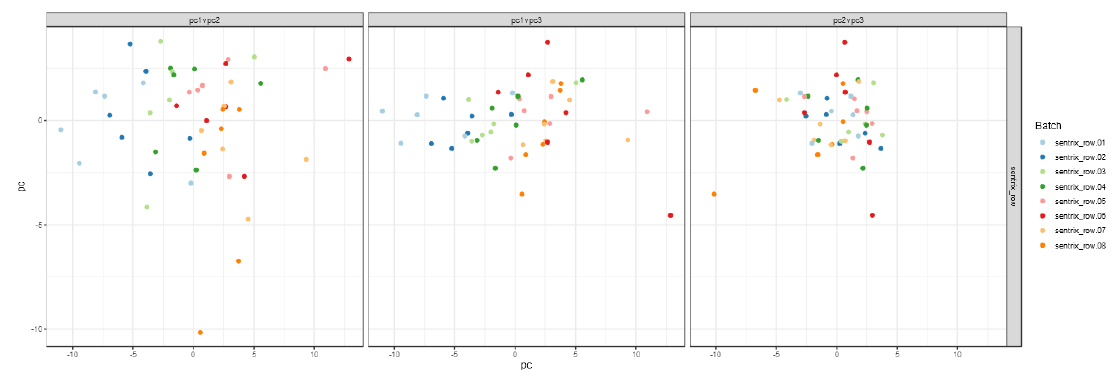

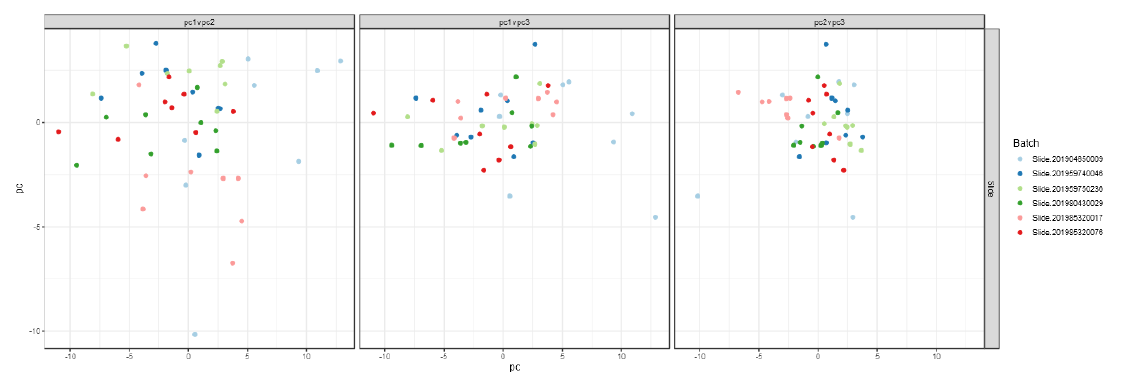


**2b**

**2a**

*Supplementary Figures 2a & 2b:* Principal component (PC) plots for the six slides used in the epigenome-wide association study (EWAS) and the eight rows to which samples were assigned. The figure is divided into three parts representing the first three PCs. Each dot represents one of the 48 samples included in the EWAS and each colour represents one of the six arrays used in the EWAS (in Supplementary Figure 2a) and each of the eight rows (in Supplementary Figure 2b).


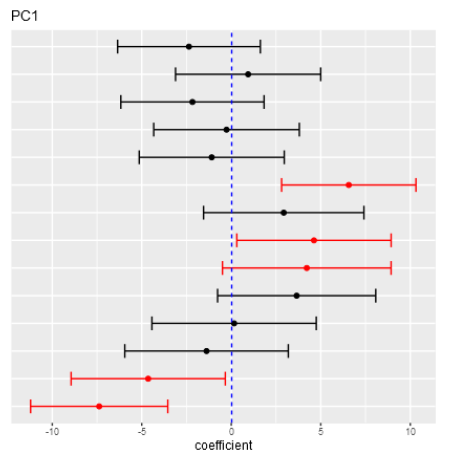

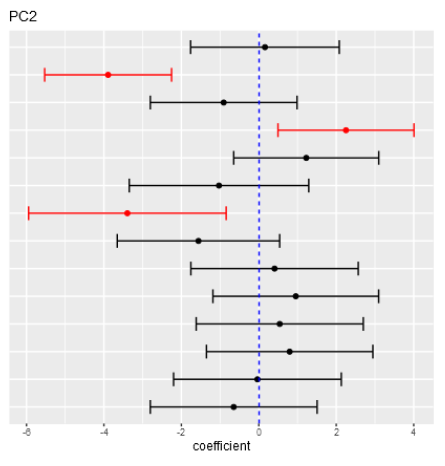


**3b**

**3a**


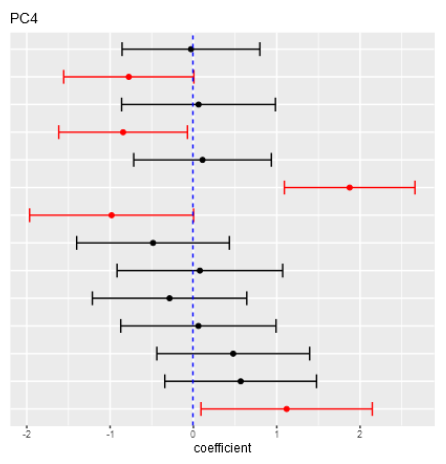

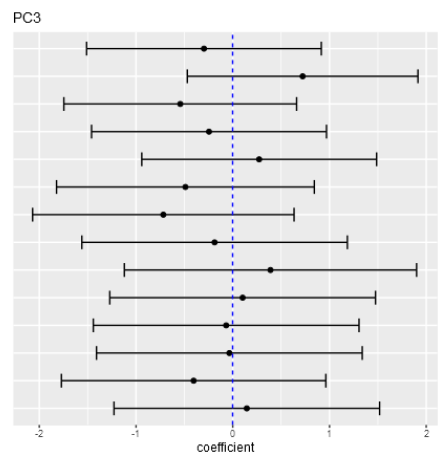


**3d**

**3c**


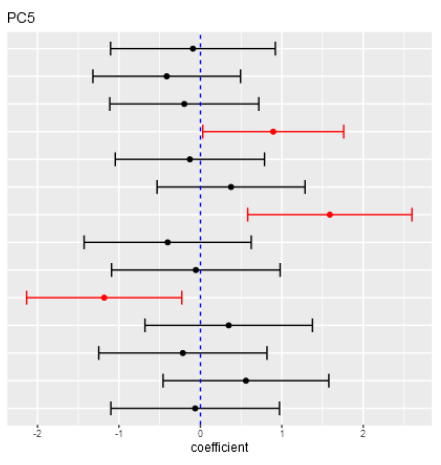


**3e**

*Supplementary Figures 3a-e:* Forrest plots representing regression coefficients and 95% confidence intervals (*x*-axis) before functional normalisation for each slide (bar 1 to 6) and each row (bar 7 to 14) divided into five figures representing principal component 1 (PC1; (Supplementary Figure 3a), PC2 (Supplementary Figure 3b), PC3 (Supplementary Figure 3c), PC4 (Supplementary Figure 3d) and PC5 (Supplementary Figure 3e).


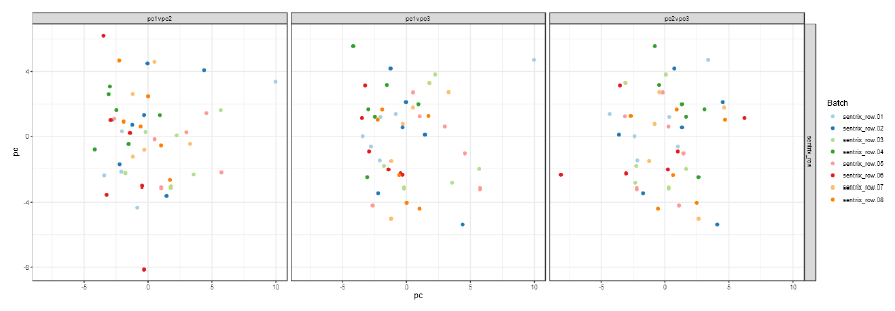

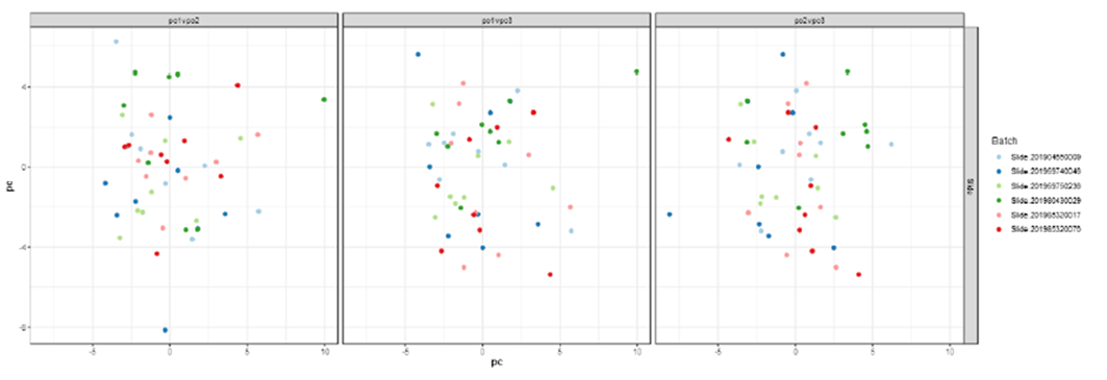
*Supplementary Figures 4a & 4b:* Principal component (PC) plots following functional normalisation for the six slides used in the epigenome-wide association study (EWAS) and the eight rows to which samples were assigned. The figure is divided into three parts representing the first three PCs. Each dot represents one of the 48 samples included in the EWAS and each colour represents one of the six arrays used in the EWAS in Supplementary Figure 4a and each of the eight rows in Supplementary Figure 4b.

**4b**

**4a**


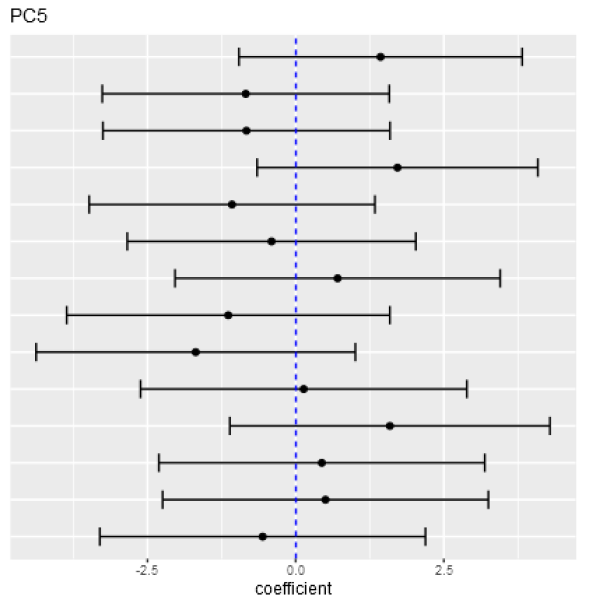

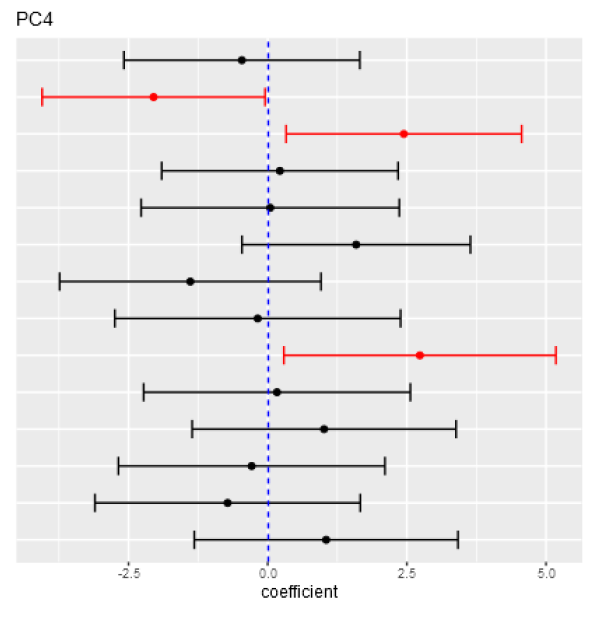

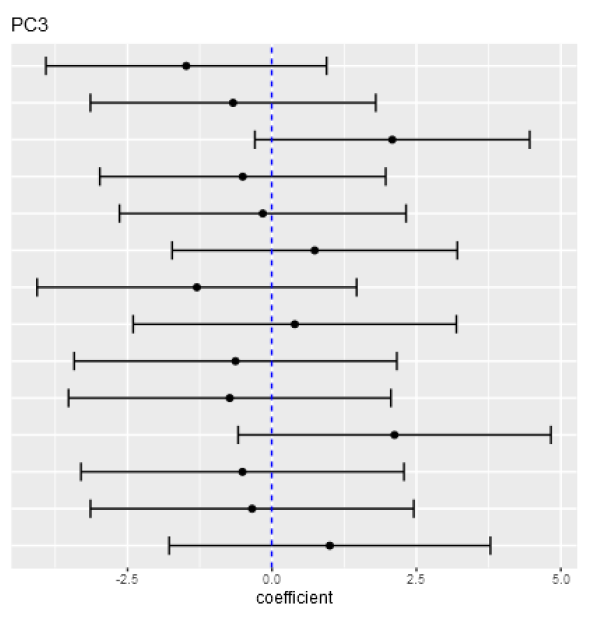

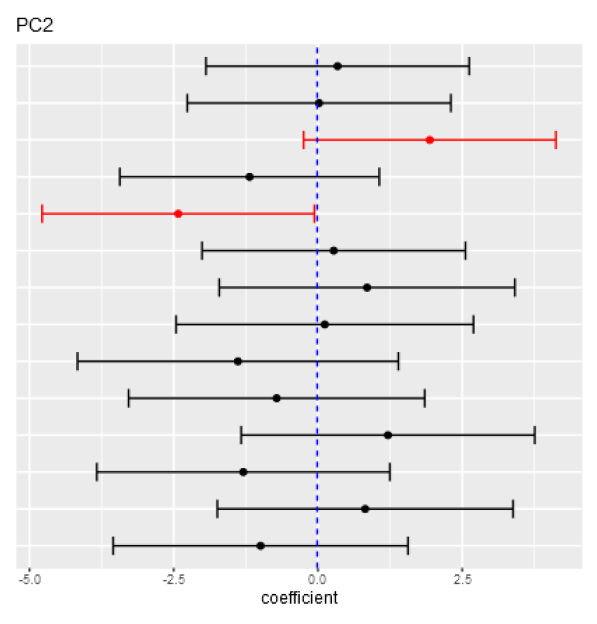

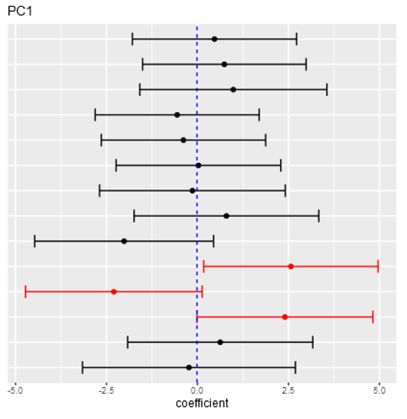


**5e**

**5d**

**5c**

**5b**

**5a**

*Supplementary Figures 5a-e:* Forrest plots representing regression coefficients and 95% confidence intervals (*x*-axis) after functional normalisation for each slide (bar 1 to 6) and each row (bar 7 to 14) divided into five figures representing principal component 1 (PC1; Supplementary Figure 5a), PC2 (Supplementary Figure 5b), PC3 (Supplementary Figure 5c), PC4 (Supplementary Figure 5d) and PC5 (Supplementary Figure 5e).


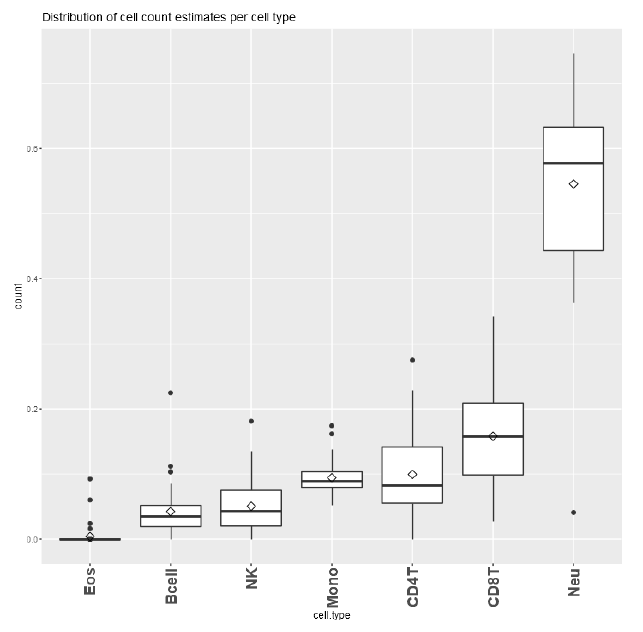


*Supplementary Figure 6:* Boxplots illustrating the estimated cellular composition for each reference cell type across all samples. The *x*-axis represents the cell type (eosinophils, B lymphocytes, natural killer cells, monocytes, CD4 T-cells, CD8 T-cells and neutrophils). The *y*-axis represents the estimated cell proportions.


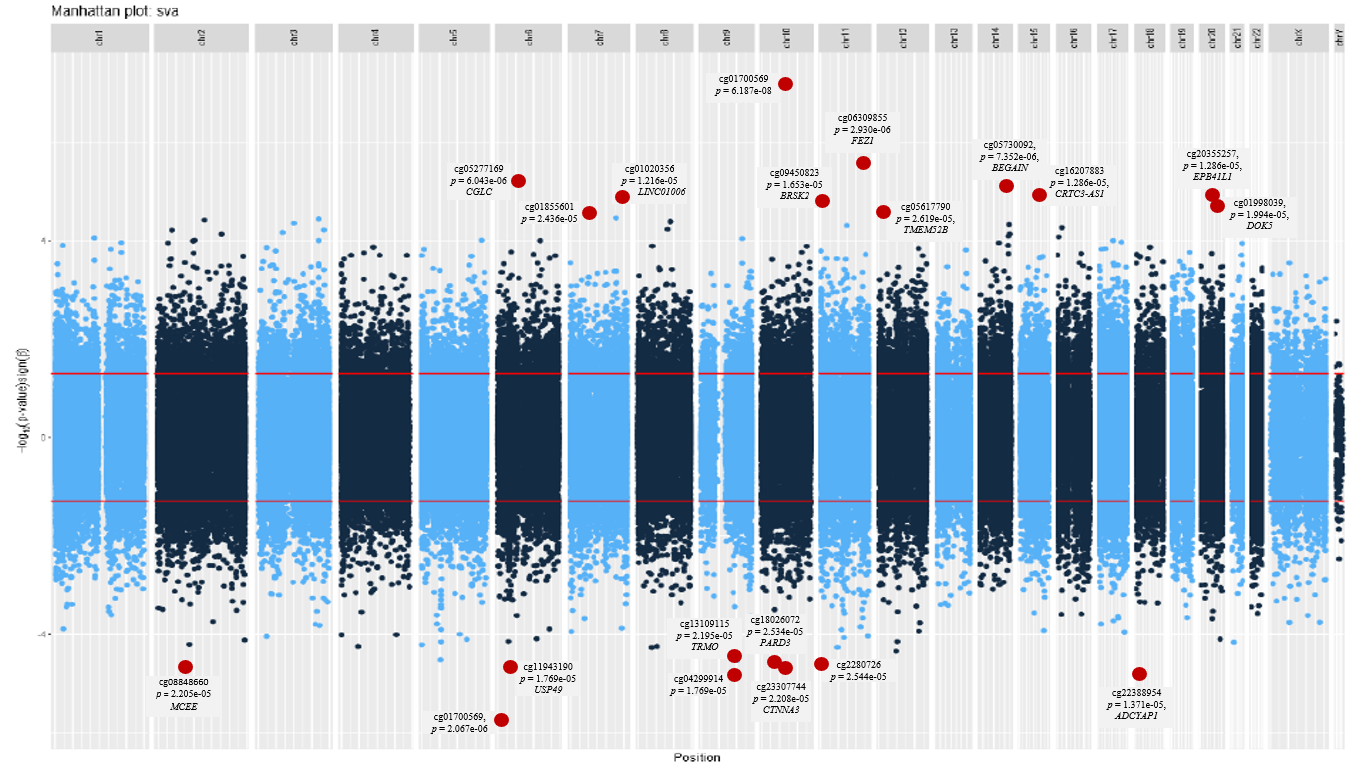
*Supplementary Figure 7*: Manhattan plot indicating the top twenty CpG sites significantly associated with PTSD prior to correction for multiple testing (*p* < .05). CpG sites are arranged according to their chromosomal position on the horizontal axis. CpG sites above the upper red line indicates that the site showed increased methylation in the group without PTSD and CpG sites below the lower red line indicates that the site showed decreased methylation in the group without PTSD.


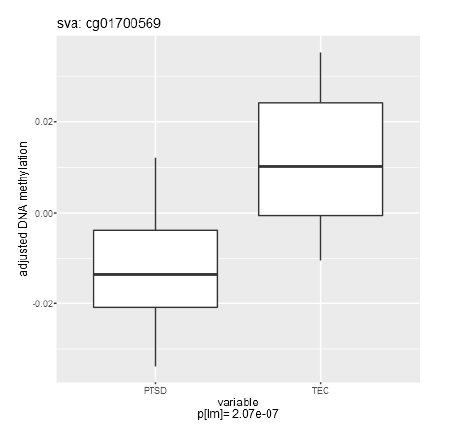


*Supplementary Figure 8a:* Boxplot illustrating the difference in methylation levels between participants with and without PTSD at 3-months post-rape for the intergenic CpG site located near *SLC16A9*.


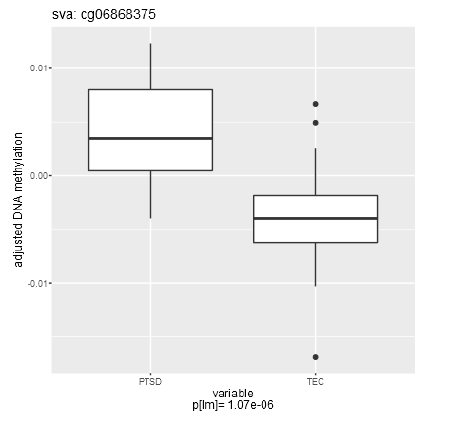


*Supplementary Figure 8b:* Boxplot illustrating the difference in methylation levels between participants with and without PTSD at 3-months post-rape for the intergenic CpG site located near *IRF4*.


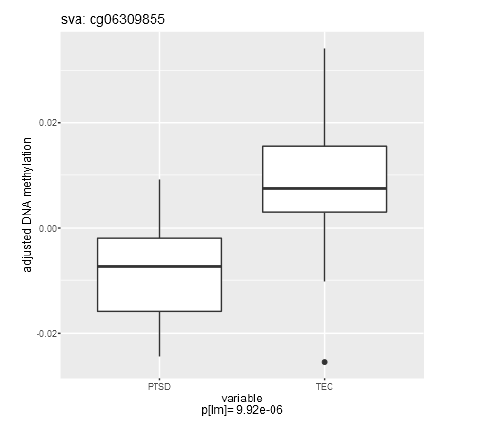


*Supplementary Figure 8c:* Boxplot illustrating the difference in methylation levels between participants with and without PTSD at 3-months post-rape for the CpG site in *FEZ1.*


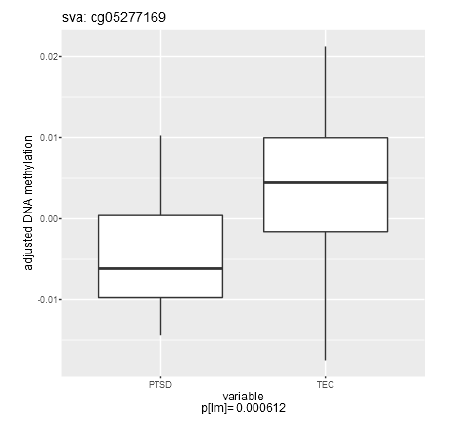


*Supplementary Figure 8d:* Boxplot illustrating the difference in methylation levels between participants with and without PTSD at 3-months post-rape for the CpG site in *GCLC.*


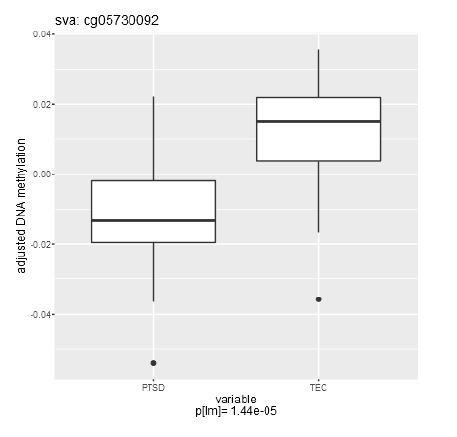


*Supplementary Figure 8e:* Boxplot illustrating the difference in methylation levels between participants with and without PTSD at 3-months post-rape for the CpG site in *BEGAIN.*


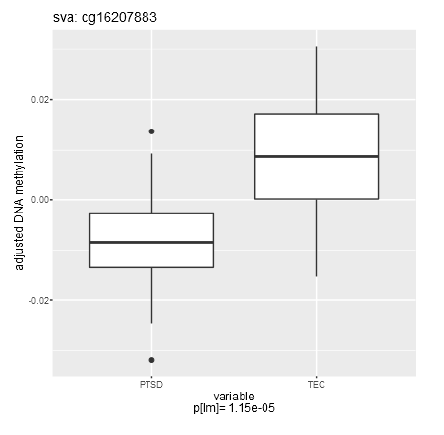


*Supplementary Figure 8f:* Boxplot illustrating the difference in methylation levels between participants with and without PTSD at 3-months post-rape for the CpG site in *CRTC3-AS1.*


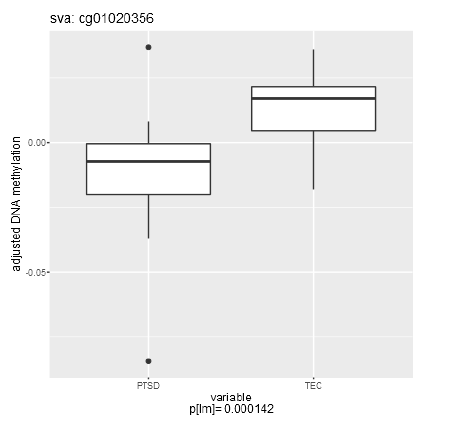


*Supplementary Figure 8g:* Boxplot illustrating the difference in methylation levels between participants with and without PTSD at 3-months post-rape for the CpG site in *LINC01006.*


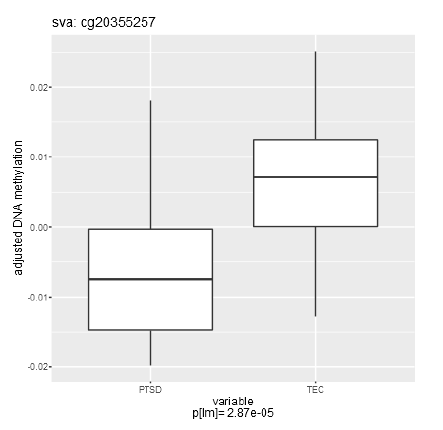


*Supplementary Figure 8h:* Boxplot illustrating the difference in methylation levels between participants with and without PTSD at 3-months post-rape for the CpG site in *EPB41L1.*


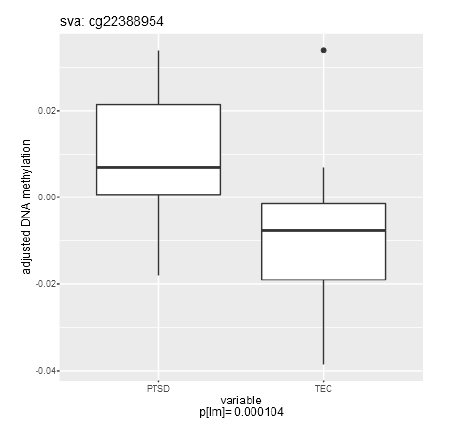


*Supplementary Figure 8i:* Boxplot illustrating the difference in methylation levels between participants with and without PTSD at 3-months post-rape for the CpG site in *ADCYAP1.*


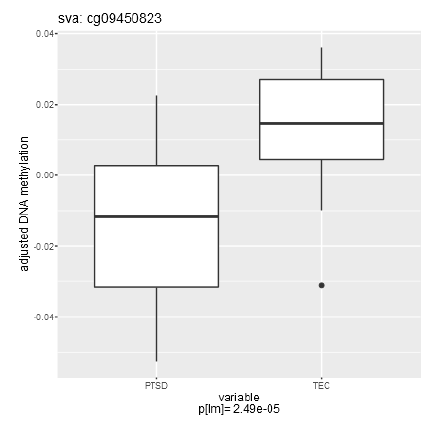


*Supplementary Figure 8j:* Boxplot illustrating the difference in methylation levels between participants with and without PTSD at 3-months post-rape for the CpG site in *BRSK2.*


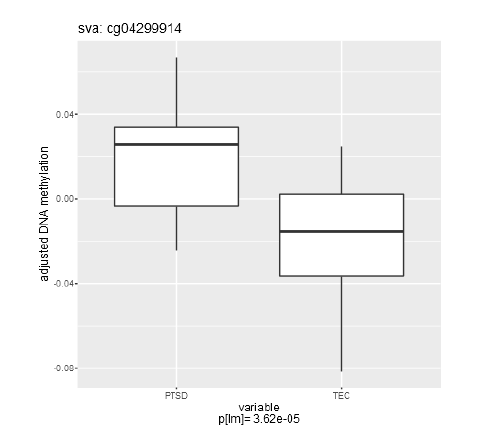


*Supplementary Figure 8k:* Boxplot illustrating the difference in methylation levels between participants with and without PTSD at 3-months post-rape for the intergenic CpG site located near *MIR4290.*


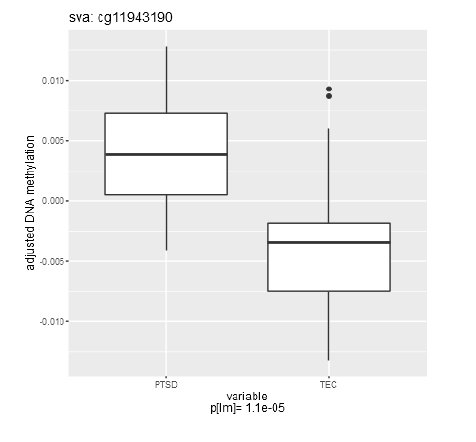


*Supplementary Figure 8l:* Boxplot illustrating the difference in methylation levels between participants with and without PTSD at 3-months post-rape for the CpG site in *USP49.*


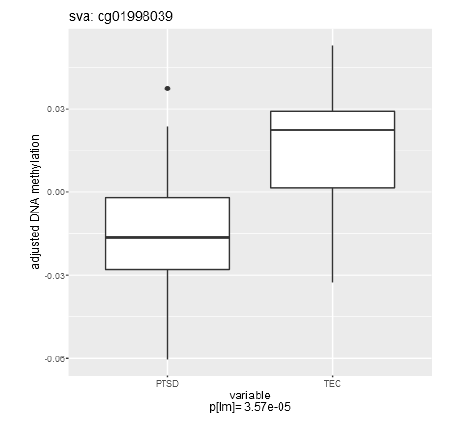


*Supplementary Figure 8m:* Boxplot illustrating the difference in methylation levels between participants with and without PTSD at 3-months post-rape for the CpG site in *DOK5.*


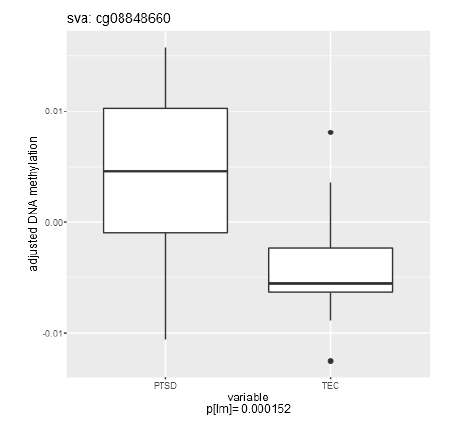


*Supplementary Figure 8n:* Boxplot illustrating the difference in methylation levels between participants with and without PTSD at 3-months post-rape for the CpG site in *MCEE.*


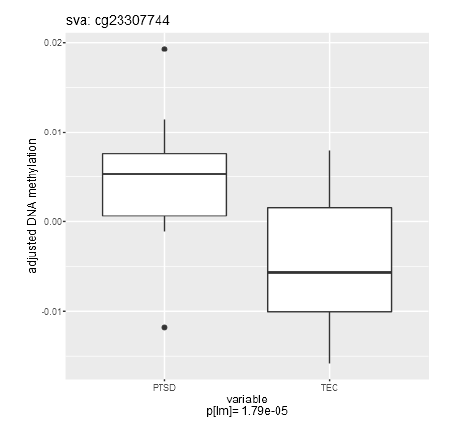


*Supplementary Figure 8o:* Boxplot illustrating the difference in methylation levels between participants with and without PTSD at 3-months post-rape for the CpG site in *CTNNA3.*


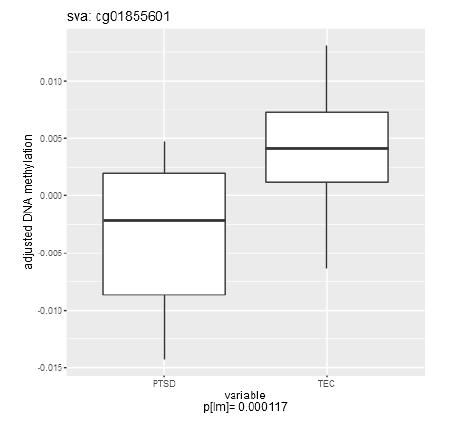


*Supplementary Figure 8p:* Boxplot illustrating the difference in methylation levels between participants with and without PTSD at 3-months post-rape for the intergenic CpG site located near *AC004854.2.*


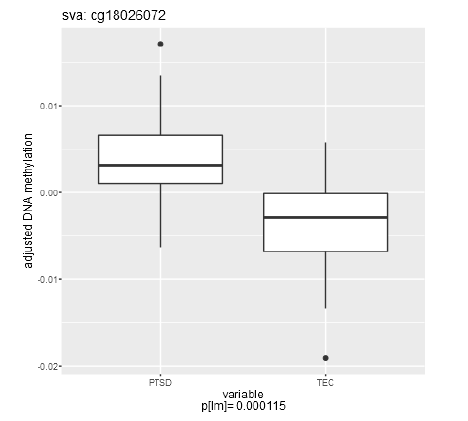


*Supplementary Figure 8q:* Boxplot illustrating the difference in methylation levels between participants with and without PTSD at 3-months post-rape for the CpG site in *PARD3.*


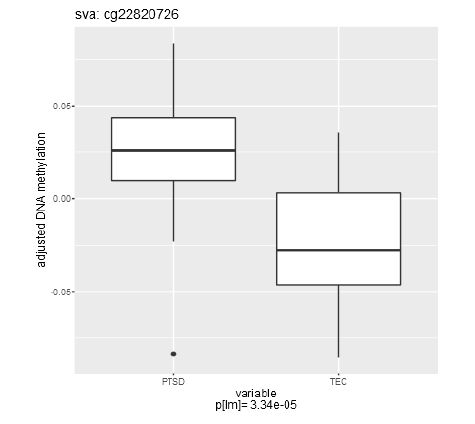


*Supplementary Figure 8r:* Boxplot illustrating the difference in methylation levels between participants with and without PTSD at 3-months post-rape for the intergenic CpG site located near *BRSK2.*


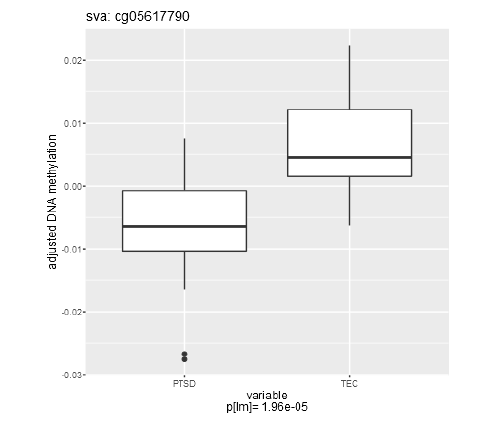


*Supplementary Figure 8s:* Boxplot illustrating the difference in methylation levels between participants with and without PTSD at 3-months post-rape for the CpG site in *TMEM52B.*


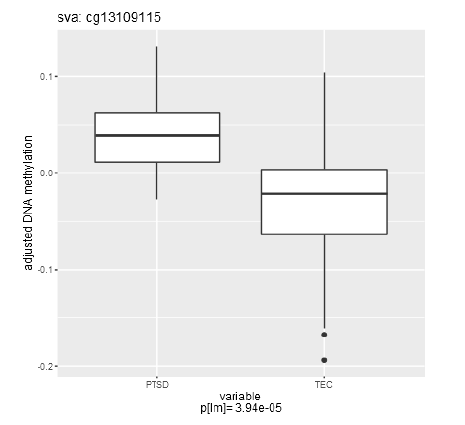


*Supplementary Figure 8t:* Boxplot illustrating the difference in methylation levels between participants with and without PTSD at 3-months post-rape for the CpG site in *TRMO.*


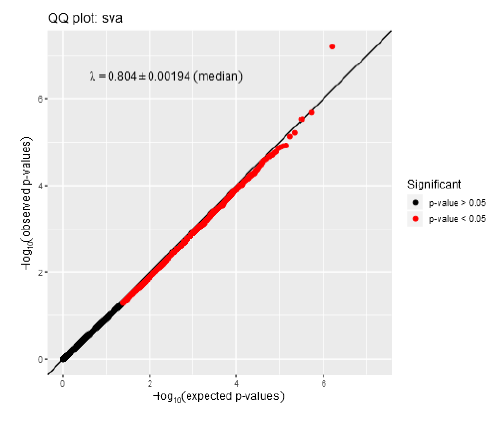


*Figure 9:* QQ plot presenting the epigenome-wide association study findings.


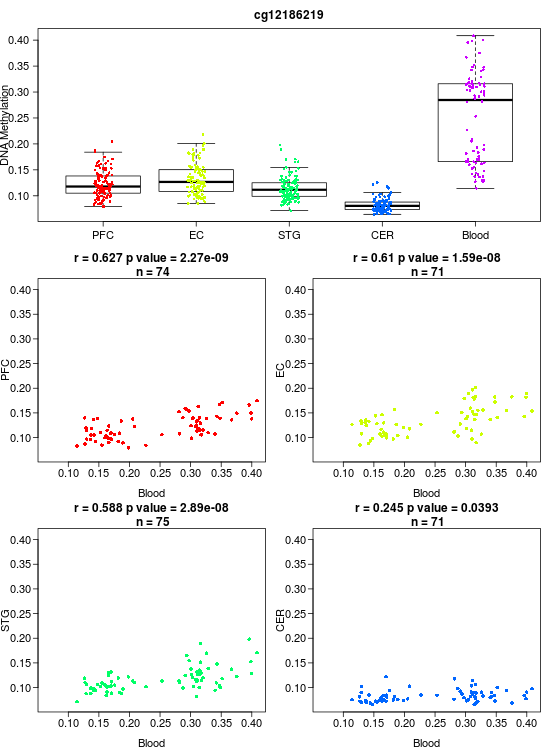


*Figure 10a:* Correlation between methylation levels observed in blood and methylation levels observed in the prefrontal cortex (PFC), entorhinal cortex (EC), superior temporal gyrus (STG) and the cerebellum (CER) for *BRSK2* CpG1 (cg12186219).


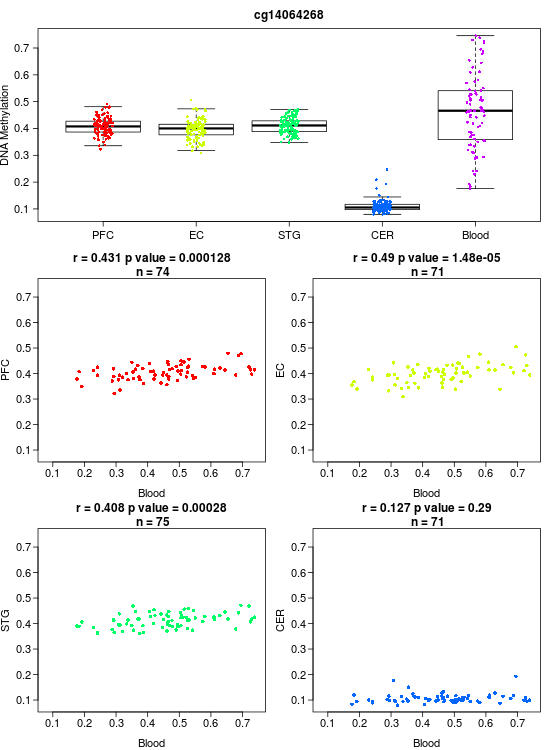


*Figure 10b:* Correlation between methylation levels observed in blood and methylation levels observed in the prefrontal cortex (PFC), entorhinal cortex (EC), superior temporal gyrus (STG) and the cerebellum (CER) for *BRSK2* CpG2 (cg14064268).


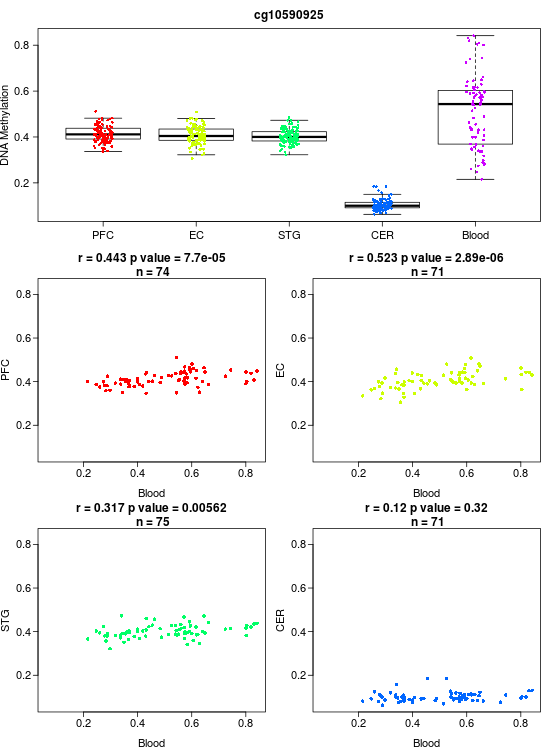


*Figure 10c:* Correlation between methylation levels observed in blood and methylation levels observed in the prefrontal cortex (PFC), entorhinal cortex (EC), superior temporal gyrus (STG) and the cerebellum (CER) for *BRSK2* CpG3 (cg10590925).


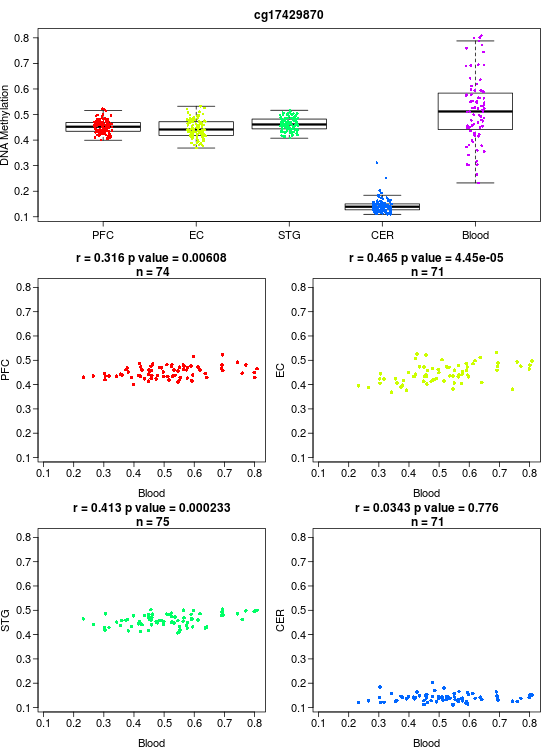


*Figure 10d:* Correlation between methylation levels observed in blood and methylation levels observed in the prefrontal cortex (PFC), entorhinal cortex (EC), superior temporal gyrus (STG) and the cerebellum (CER) for *BRSK2* CpG4 (cg17429870).


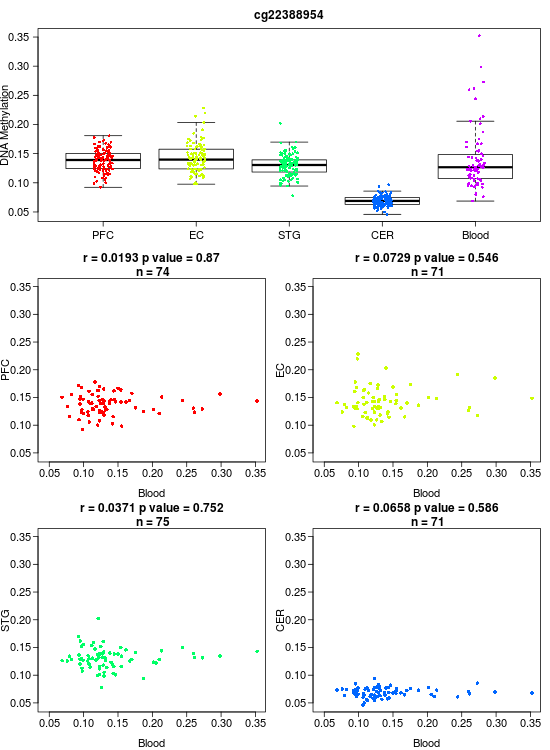


*Figure 11a:* Correlation between methylation levels observed in blood and methylation levels observed in the prefrontal cortex (PFC), entorhinal cortex (EC), superior temporal gyrus (STG) and the cerebellum (CER) for *ADCYAP1* CpG1 (cg22388954).


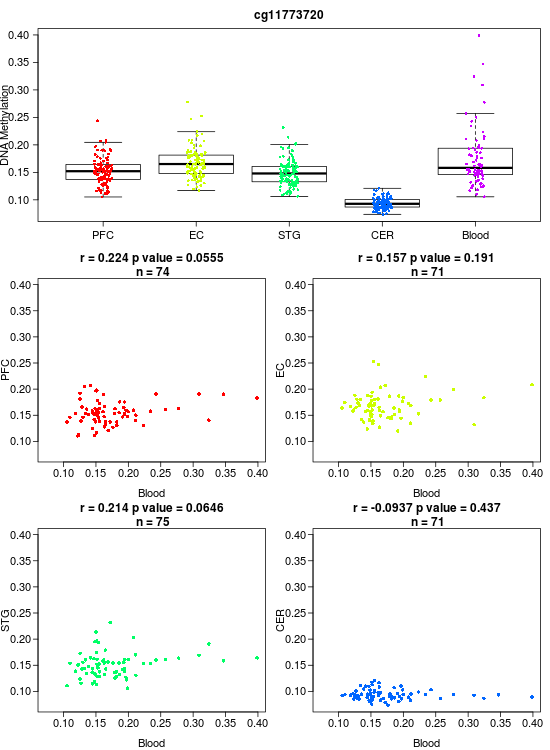


*Figure 11b:* Correlation between methylation levels observed in blood and methylation levels observed in the prefrontal cortex (PFC), entorhinal cortex (EC), superior temporal gyrus (STG) and the cerebellum (CER) for *ADCYAP1* CpG2 (cg11773720).

Supplementary Table 1: *Association between principal components and batch variables before functional normalisation*

| Batch variable | Batch variable subcategory | Principal Component (PC) | *F* | *t* | *p* | 95% Confidence Interval (CI)  Lower Upper | |
| --- | --- | --- | --- | --- | --- | --- | --- |
|  |  |  |  |  |  |  |  |
| Slide |  | PC1 | 3.54 |  | .009 |  |  |
|  | 210904650009 | PC1 |  | 6.56 | .0002 | 2.81 | 10.32 |
| Row |  | PC1 | 7.91 |  | .000005 |  |  |
|  | Sentrix row 01 | PC1 |  | -7.38 | .00006 | -11.21 | -3.55 |
|  | Sentrix row 02 | PC1 |  | -4.64 | .017 | -8.95 | -0.33 |
|  | Sentrix row 06 | PC1 |  | 4.22 | .045 | -0.49 | 8.93 |
|  | Sentrix row 07 | PC1 |  | 4.62 | .018 | 0.31 | 8.93 |
| Slide |  | PC2 | 5.06 |  | .001 |  |  |
|  | 201959750236 | PC2 |  | 2.25 | .005 | 0.50 | 4.01 |
|  | 201985320017 | PC2 |  | -3.89 | .000002 | -5.53 | -2.26 |
| Row | Sentrix row 08 | PC2 |  | -3.39 | .004 | -5.95 | -0.84 |
| Slide |  | PC4 | 6.35 |  | .0002 |  |  |
|  | 210904650009 | PC4 |  | 1.87 | .000002 | 1.09 | 2.66 |
|  | 201985320017 | PC4 |  | -0.78 | .028 | -1.56 | 0.01 |
| Row | Sentrix row 01 | PC4 |  | 1.12 | .016 | 0.01 | 2.15 |
|  | Sentrix row 08 | PC4 |  | -0.98 | .027 | -1.97 | 0.00 |
| Slide | 201959750236 | PC5 |  | 0.90 | .022 | 0.03 | 1.76 |
| Row |  | PC5 | 3.31 |  | .007 |  |  |
|  | Sentrix row 05 | PC5 |  | -1.18 | .007 | -2.14 | -0.23 |
|  | Sentrix row 08 | PC5 |  | 1.59 | .0007 | 0.58 | 2.60 |
|  |  |  |  |  |  |  |  |

Supplementary Table 2: *Association between principal components and batch variables after functional normalisation*

| Batch variable | Batch variable subcategory | Principal Component (PC) | *t* | *p* | 95% Confidence Interval (CI)  Lower Upper | |
| --- | --- | --- | --- | --- | --- | --- |
|  |  |  |  |  |  |  |
| Row | Sentrix row 03 | PC1 | 2.40 | .027 | -0.01 | 4.82 |
|  | Sentric row 04 | PC1 | -2.30 | .035 | -4.73 | 0.13 |
|  | Sentrix row 05 | PC1 | 2.57 | .018 | 0.17 | 4.96 |
| Slide | 201959740046 | PC2 | -2.42 | .023 | -4.78 | -0.06 |
|  | 201980430029 | PC2 | 1.94 | .048 | -0.25 | 4.12 |
|  | 201980430029 | PC4 | 2.45 | .011 | 0.33 | 4.56 |
|  | 201985320017 | PC4 | -2.05 | .023 | -4.05 | .0.05 |
| Row | Sentrix row 06 | PC4 | 2.73 | .014 | 0.29 | 5.18 |
|  |  |  |  |  |  |  |

Supplementary Table 3: *Epigenome-wide differentially methylated positions (DMPs) and regions (DMRs) associated with PTSD at 3-months post-rape*

| Gene Name^1^ | Position^2^ | Probe | Location in Gene^3,4^ | *β* | SE | *t/z* | *p* | Adj. *p* | Other exposures/  phenotypes associated with the CpG site^5^ |
| --- | --- | --- | --- | --- | --- | --- | --- | --- | --- |
|  |  |  |  |  |  |  |  |  |  |
| **Differentially methylated positions (DMPs)** | | |  |  |  |  |  |  |  |
|  |  |  |  |  |  |  |  |  |  |
| NA, *SLC16A9*^6^ | Chr10:61385771 | cg01700569 | Intergenic | 0.031 | 0.004 | 7.119 | 6.187e-08 | 0.049233 | None |
| NA, *IRF4*^6^ | Chr6:429318 | cg06868375 | Intergenic | -0.009 | 0.002 | -5.846 | 2.067e-06 | 0.777284 | None |
| *FEZ1* | Chr11:125365803 | cg06309855 | 5'UTR | 0.022 | 0.004 | 5.727 | 2.930e-06 | 0.777284 | Gestational age |
| *GCLC* | Chr6:53371893 | cg05277169 | Intergenic | 0.013 | 0.002 | 5.468 | 6.043e-06 | 0.999998 | None |
| *BEGAIN* | Chr14:101036470 | cg05730092 | TSS1500 | 0.032 | 0.006 | 5.401 | 7.352e-06 | 0.999998 | Gestational age |
| *CRTC3-AS1* | Chr15:91193536 | cg16207883 | Body | 0.021 | 0.004 | 5.233 | 1.183e-05 | 0.999998 | None |
| *LINC01006* | Chr7:156267149 | cg01020356 | Body | 0.025 | 0.005 | 5.224 | 1.216e-05 | 0.999998 | Ethnicity; osteonecrosis of the femoral head |
| *EPB41L1* | Chr20:34818105 | cg20355257 | 3'UTR | 0.017 | 0.003 | 5.204 | 1.286e-05 | 0.999998 | Down syndrome |
| *ADCYAP1* | Chr18:905177 | cg22388954 | 5'UTR; TSS200 | -0.025 | 0.005 | -5.181 | 1.371e-05 | 0.999998 | B acute lymphoblastic leukemia |
| *BRSK2* | Chr11:1431833 | cg09450823 | Body | 0.036 | 0.007 | 5.115 | 1.653e-05 | 0.999998 | None |
| NA, *MIR4290*^6^ | Chr9:92782655 | cg04299914 | Intergenic | -0.049 | 0.010 | -5.091 | 1.769e-05 | 0.999998 | None |
| *USP49* | Chr6:41790313 | cg11943190 | 5'UTR | -0.010 | 0.002 | -5.050 | 1.970e-05 | 0.999998 | Rheumatoid arthritis; exercise |
| *DOK5* | Chr20:53127455 | cg01998039 | 5'UTR; Body | 0.040 | 0.008 | 5.049 | 1.994e-05 | 0.999998 | Down syndrome; aging |
| *MCEE* | Chr2:71338735 | cg08848660 | Body | -0.011 | 0.002 | -5.012 | 2.205e-05 | 0.999998 | None |
| *CTNNA3* | Chr10:68940214 | cg23307744 | Body | -0.012 | 0.002 | -5.012 | 2.208e-05 | 0.999998 | None |
| NA, *AC004854.2*^6^ | Chr7:44965393 | cg01855601 | Intergenic | 0.010 | 0.002 | 4.975 | 2.436e-05 | 0.999998 | None |
| *PARD3* | Chr10:35016204 | cg18026072 | Body | -0.009 | 0.002 | -4.961 | 2.534e-05 | 0.999998 | None |
| NA, *BRSK2*^6^ | Chr11:1401914 | cg22820726 | Intergenic | -0.063 | 0.013 | -4.963 | 2.544e-05 | 0.999998 | None |
| *TMEM52B* | Chr12:10322100 | cg05617790 | Body; TSS1500 | 0.015 | 0.003 | 4.953 | 2.619e-05 | 0.999998 | None |
| *TRMO* | Chr9:100662939 | cg13109115 | Intergenic | -0.103 | 0.021 | -4.915 | 2.915e-05 | 0.999998 | None |
|  |  |  |  |  |  |  |  |  |  |
| **Differentially methylated regions (DMRs)** | | |  |  |  |  |  |  |  |
|  |  |  |  |  |  |  |  |  |  |
| *LRRC34* | Chr3:169530817-169530920 |  |  | 0.071 | 0.007 | 9.631 | 5.910e-22 | 4.897e-16 |  |
|  |  | cg16024214^7^ | TSS1500 |  |  |  |  |  | None |
|  |  | cg27467234^7^ | TSS1500 |  |  |  |  |  | None |
|  |  | cg04714994^7^ | TSS1500 |  |  |  |  |  | Ethnicity |
|  |  | cg05344026^7^ | TSS1500 |  |  |  |  |  | Ethnicity |
| *LRRC34* | Chr3:169531663-169531864 |  |  | 0.091 | 0.010 | 9.101 | 8.960e-20 | 7.424e-14 |  |
|  |  | cg12324144^7^ | TSS1500 |  |  |  |  |  | None |
|  |  | cg03369965^7^ | TSS1500 |  |  |  |  |  | Follicular thyroid carcinoma; type 2 diabetes; ethnicity |
|  |  | cg13337095^7^ | TSS1500 |  |  |  |  |  | None |
|  |  | cg10994914^7^ |  |  |  |  |  |  | B Acute lymphoblastic leukemia |
| *PLD6* | Chr17:17109640-17109817 |  |  | 0.026 | 0.003 | 9.316 | 1.214e-20 | 1.006e-14 |  |
|  |  | cg03292213^7^ | 5'UTR |  |  |  |  |  | Ankylosing spondylitis |
|  |  | cg11392858^7^ | TSS200 |  |  |  |  |  | Ankylosing spondylitis |
|  |  | cg23661344^7^ | TSS200 |  |  |  |  |  | Ankylosing spondylitis |
|  |  | cg26539818^7^ | TSS200 |  |  |  |  |  | Ankylosing spondylitis |
|  |  | cg00572586^7^ | TSS200 |  |  |  |  |  | Juice consumption |
|  |  | cg14099398^7^ | TSS200 |  |  |  |  |  | None |
|  |  | cg02880176^7^ | TSS200 |  |  |  |  |  | None |
| NA, *LOC101*  *928343*^6^ | Chr17:72595585-72595587 |  |  | 0.093 | 0.011 | 8.166 | 3.191e-16 | 2.644e-10 |  |
|  |  | cg01238395 | Intergenic |  |  |  |  |  | None |
|  |  | cg05192716 | Intergenic |  |  |  |  |  | None |
| *SLC38A11* | Chr2:165811982-165812244 |  |  | -0.022 | 0.003 | -7.718 | 1.178e-14 | 9.762e-09 |  |
|  |  | cg03464655 | 5'UTR |  |  |  |  |  | Adrenocortical carcinoma; Nicolaides Baraitser syndrome (NCBRS); neurodevelopmental presentations and congenital anomalies; Gulf War illness |
|  |  | cg20641955 | TSS200 |  |  |  |  |  | None |
|  |  | cg16376155 | TSS200 |  |  |  |  |  | None |
|  |  | cg05339727 | TSS200 |  |  |  |  |  | Age; sex; ethnicity; *SETD1-B*-related syndrome |
|  |  | cg05090759 | TSS200 |  |  |  |  |  | Clear cell renal carcinoma; sex; *SETD1-B*-related syndrome |
|  |  | cg12301695 | TSS200 |  |  |  |  |  | Oral squamous cell carcinoma; colorectal cancer; *SETD1-B*-related syndrome; Gulf War illness |
|  |  | cg12753297 | TSS1500 |  |  |  |  |  | None |
| *CC2D2A* | Chr4:15471214-15471399 |  |  | 0.015 | 0.002 | 7.856 | 3.964e-15 | 3.285e-09 |  |
|  |  | cg21329975 | TSS1500 |  |  |  |  |  | Smoking status;  air population exposure;  gestational age |
|  |  | cg16509355 | TSS1500 |  |  |  |  |  | Fetal vs adult liver;  smoking status;  gestational age; Down syndrome |
|  |  | cg21123203 | Intergenic |  |  |  |  |  | Smoking status;  fruit consumption |
|  |  | cg02964094 | TSS200 |  |  |  |  |  | Gingivobuccal oral squamous cell carcinoma; Gulf War illness |
|  |  | cg18470593 | TSS200 |  |  |  |  |  | Fetal vs adult liver;  smoking status; obesity |
|  |  | cg20184469 | TSS200 |  |  |  |  |  | None |
| *ESM1* | Chr5:54281478-54281733 |  |  | -0.042 | 0.004 | -9.746 | 9.935e-13 | 8.233e-07 |  |
|  |  | cg21180956 | TSS200 |  |  |  |  |  | None |
|  |  | cg13106512 | TSS200 |  |  |  |  |  | Myalgic encephalomyelitis / chronic fatigue syndrome |
|  |  | cg24403549 | TSS200 |  |  |  |  |  | Myalgic encephalomyelitis / chronic fatigue syndrome; ethnicity |
|  |  | cg10631947 | TSS1500 |  |  |  |  |  | Height |
|  |  | cg20059697 | TSS1500 |  |  |  |  |  | Down syndrome |
|  |  | cg16462183 | TSS1500 |  |  |  |  |  | Gestational age; Down syndrome; hepatocellular carcinoma; colorectal laterally spreading tumour; perinatally-acquired HIV |
| *SORBS2* | Chr4:186732837-186733060 |  |  | 0.047 | 0.006 | 7.452 | 9.180e-14 | 7.607e-08 |  |
|  |  | cg12309668 | 5'UTR; TSS1500 |  |  |  |  |  | Down syndrome; early childhood development |
|  |  | cg04392082 | 5'UTR; TSS1500 |  |  |  |  |  | Down syndrome; gestational age |
|  |  | cg02790305 | 5'UTR; TSS1500 |  |  |  |  |  | Down syndrome; aging; gestational age |
|  |  | cg26785346 | Intergenic |  |  |  |  |  | Down syndrome; early childhood development  gestational age |
|  |  | cg01933073 | 5'UTR; TSS1500 |  |  |  |  |  | Down syndrome; gestational age |
|  |  | cg18342119 | Intergenic |  |  |  |  |  | Down syndrome; aging |
|  |  | cg13921444 | 5'UTR; TSS1500 |  |  |  |  |  | Down syndrome; aging; respiratory allergies; early childhood development; gestational age |
|  |  | cg04348265 | 5'UTR; TSS1500 |  |  |  |  |  | Down syndrome; aging; early childhood development |
| *ANKRD33* | Chr12:52281674-52282079 |  |  | 0.030 | 0.004 | 7.499 | 6.442e-14 | 5.338e-08 |  |
|  |  | cg12053065 | TSS200 |  |  |  |  |  | None |
|  |  | cg19974223 | TSS200 |  |  |  |  |  | None |
|  |  | cg09755932 | TSS200 |  |  |  |  |  | None |
|  |  | cg10384244 | TSS200 |  |  |  |  |  | None |
|  |  | cg19948393 | 5'UTR |  |  |  |  |  | Down syndrome |
|  |  | cg26279928 | 5'UTR |  |  |  |  |  | None |
|  |  | cg04349021 | 5'UTR |  |  |  |  |  | None |
|  |  | cg17424856 | 5'UTR |  |  |  |  |  | None |
|  |  | cg03110082 | 5'UTR |  |  |  |  |  | None |
| *COL18A1* | Chr21:46875372-46875500 |  |  | 0.029 | 0.004 | 7.373 | 1.671e-13 | 1.385e-07 |  |
|  |  | cg24201814 | TSS200; Body |  |  |  |  |  | None |
|  |  | cg06552850 | TSS200; Body |  |  |  |  |  | None |
|  |  | cg07279557 | TSS200; Body |  |  |  |  |  | Down syndrome; fetal vs adult liver;  early childhood development; gestational age;  maternal smoking; sex;  neurodevelopmental presentations and congenital anomalies |
|  |  | cg04401043 | TSS200; Body |  |  |  |  |  | None |
|  |  | cg11029358 | 5'UTR; Body |  |  |  |  |  | Gestational age; neurodevelopmental presentations and congenital anomalies |
|  |  | cg07641662 | Body |  |  |  |  |  | None |
| *BRSK2* | Chr11:1463541-1463670 |  |  | 0.112 | 0.016 | 7.131 | 9.935e-13 | 8e-07 |  |
|  |  | cg12186219 | Body |  |  |  |  |  | Childhood stress; ethnicity |
|  |  | cg14064268 | Body |  |  |  |  |  | Aging; childhood stress; ethnicity |
|  |  | cg10590925 | Body |  |  |  |  |  | Aging, childhood stress, ethnicity |
|  |  | cg17429870 | Body |  |  |  |  |  | Aging, childhood stress, ethnicity |
|  |  | cg18651858 | Body |  |  |  |  |  | Ethnicity |
| *AC004895.4* | Chr7:6120483-6120572 |  |  | -0.053 | 0.008 | -6.864 | 6.686e-12 | 5.5e-06 |  |
|  |  | cg27459591^7^ | NSF |  |  |  |  |  | Down syndrome |
|  |  | cg10437320^7^ | NSF |  |  |  |  |  | Down syndrome |
|  |  | cg07374086^7^ | NSF |  |  |  |  |  | Down syndrome |
|  |  | cg14614937^7^ | NSF |  |  |  |  |  | None |
| *SPON1* | Chr11:13983705-13983989 |  |  | -0.021 | 0.003 | -6.857 | 7.040e-12 | 5.8e-06 |  |
|  |  | cg08309747 | TSS1500 |  |  |  |  |  | None |
|  |  | cg26255604 | TSS1500 |  |  |  |  |  | None |
|  |  | cg12085698 | TSS200 |  |  |  |  |  | Pancreatic ductal adenocarcinoma;  gestational age; inflamed Crohn’s disease |
|  |  | cg25486824 | TSS200 |  |  |  |  |  | Pancreatic ductal adenocarcinoma; gestational age; sex; inflamed Crohn’s disease |
|  |  | cg11028624 | TSS200 |  |  |  |  |  | Pancreatic ductal adenocarcinoma; gestational age; inflamed Crohn’s disease |
|  |  | cg22805485 | TSS200 |  |  |  |  |  | Pancreatic ductal adenocarcinoma; gestational age; maternal smoking; inflamed Crohn’s disease |
|  |  | cg24258347 | TSS200 |  |  |  |  |  | Pancreatic ductal adenocarcinoma; gestational age; aging; preterm birth; maternal hypertensive disorder; inflamed Crohn’s disease |
|  |  | cg10153349 | TSS200 |  |  |  |  |  | Gestational age; sex; prenatal paracetamol exposure; aging; inflamed Crohn’s disease |
|  |  | cg09191626 | 5'UTR |  |  |  |  |  | Gestational age; maternal smoking; follicular thyroid carcinoma |
|  |  | cg02394317 | TSS200 |  |  |  |  |  | None |
| *ESM1* | Chr5:54281198-54281362 |  |  | -0.041 | 0.006 | -6.827 | 8.662e-12 | 7.2e-06 |  |
|  |  | cg14972155 | Intergenic |  |  |  |  |  | Italian ethnicity; sex; maternal plasma folate; chronic obstructive pulmonary disease |
|  |  | cg06837426 | Intergenic |  |  |  |  |  | Maternal plasma folate |
|  |  | cg20451680 | Intergenic |  |  |  |  |  | Chronic obstructive pulmonary disease; type 2 diabetes |
|  |  | cg20673840 | 5'UTR |  |  |  |  |  | Chronic obstructive pulmonary disease; type 2 diabetes |
| *ADCYAP1* | Chr18:905177-905180 |  |  | -0.022 | 0.003 | -6.761 | 1.370e-11 | 1.13e-05 |  |
|  |  | cg22388954 | 5'UTR; TSS200 |  |  |  |  |  | B Acute lymphoblastic leukemia |
|  |  | cg11773720 | 5'UTR; TSS200 |  |  |  |  |  | None |
| *INPP5A* | Chr10:134549665-134549800 |  |  | 0.016 | 0.002 | 6.700 | 2.081e-11 | 1.72e-05 | B Acute lymphoblastic leukemia |
|  |  | cg18305652 | Body |  |  |  |  |  | B Acute lymphoblastic leukemia |
|  |  | cg16311946 | Body |  |  |  |  |  | B Acute lymphoblastic leukemia |
|  |  | cg11624078 | Body |  |  |  |  |  | None |
| *C21orf62* | Chr21:34185960-34186122 |  |  | 0.021 | 0.003 | 6.345 | 2.219e-10 | 1.839e-4 |  |
|  |  | cg03803086 | 5'UTR |  |  |  |  |  | Fetal vs adult liver; early childhood development; gestational age; sex; air pollution; body mass index; Down syndrome; follicular thyroid carcinoma; thyroid lesion |
|  |  | cg27427104 | 5'UTR |  |  |  |  |  | Werner syndrome; Gulf War illness |
|  |  | cg00780604 | 5'UTR |  |  |  |  |  | Gulf War illness |
|  |  | cg23163200 | TSS200 |  |  |  |  |  | Fetal vs adult liver; early childhood development; gestational age; multiple sclerosis; Werner syndrome |
| *LRRC34* | Chr3:169530621-169530692 |  |  | 0.050 | 0.008 | 6.184 | 6.268e-10 | 0.001 |  |
|  |  | cg02860240^7^ | TSS1500 |  |  |  |  |  | Hepatocellular carcinoma; ethnicity |
|  |  | cg24655016^7^ | TSS200 |  |  |  |  |  | Ethnicity |
|  |  | cg23707559^7^ | TSS200 |  |  |  |  |  | None |
| *RP11-1085N6.3* | Chr14:57197389-57197433 |  |  | 0.023 | 0.004 | 6.146 | 7.946e-10 | 0.001 |  |
|  |  | cg10247374 | NSF |  |  |  |  |  | None |
|  |  | cg11668797 | NSF |  |  |  |  |  | None |
|  |  | cg08934402 | NSF |  |  |  |  |  | Ethnicity |
| *TSPEAR-AS2* | Chr21:45937342-45937406 |  |  | 0.036 | 0.006 | 6.115 | 9.646e-10 | 0.001 |  |
|  |  | cg05005821 | Body |  |  |  |  |  | None |
|  |  | cg04289880 | Body |  |  |  |  |  | Sex; maternal alcohol consumption |
| NA, *SFXN3*^6^ | Chr10:102806871-102806933 |  |  | 0.042 | 0.007 | 6.029 | 1.650e-09 | 0.001 |  |
|  |  | cg04270401 | Intergenic |  |  |  |  |  | Fetal vs adult liver; early childhood development; gestational age; alcohol consumption; aging; follicular thyroid carcinoma; obesity |
|  |  | cg24067118 | Intergenic |  |  |  |  |  | Fetal vs adult liver; early childhood development; gestational age; alcohol consumption; absolute fat mass; aging; body mass index; fat free mass index; fat mass index |
| *MIR125B1* | Chr11:121970694-121970768 |  |  | 0.026 | 0.004 | 5.996 | 2.019e-09 | 0.002 |  |
|  |  | cg02101355 | Body; TSS200 |  |  |  |  |  | Clear cell renal carcinoma; psoriasis |
|  |  | cg03891346 | NSF |  |  |  |  |  | Clear cell renal carcinoma |
|  |  | cg24213115 | Body; TSS200 |  |  |  |  |  | Down syndrome |
|  |  | cg07281370 | TSS1500; Body |  |  |  |  |  | Clear cell renal carcinoma; psoriasis |
| NA, *LOC 343052*^6^ | Chr1:153762359-153762434 |  |  | 0.019 | 0.003 | 5.975 | 2.301e-09 | 0.002 |  |
|  |  | cg18042081 | Intergenic |  |  |  |  |  | None |
|  |  | cg13502252 | Intergenic |  |  |  |  |  | Gestational age; fetal alcohol spectrum disorder |
|  |  | cg11710659 | Intergenic |  |  |  |  |  | Clear cell renal carcinoma;  rheumatoid arthritis; maternal smoking; gestational age |
| NA, *LOC 343052*^6^ | Chr1:153762201-153762244 |  |  | 0.025 | 0.004 | 5.881 | 4.068e-09 | 0.003 |  |
|  |  | cg26182859 | Intergenic |  |  |  |  |  | Fetal vs adult liver; early childhood development; gestational age; smoking status; sex; acute myelocytic leukemia; aging; Crohn’s disease |
|  |  | cg09762021 | Intergenic |  |  |  |  |  | Smoking status; gestational age |
|  |  | cg12067764 | Intergenic |  |  |  |  |  | Gestational age; aging |
| *C5orf66-AS1* | Chr5:134375885-134375904 |  |  | 0.011 | 0.002 | 5.829 | 5.589e-09 | 0.005 |  |
|  |  | cg11469026 | TSS200; 5'UTR |  |  |  |  |  | None |
|  |  | cg12887021 | TSS200; 5'UTR |  |  |  |  |  | None |
| *FSCN2* | Chr17:79504142-79504180 |  |  | 0.006 | 0.001 | 5.820 | 5.874e-09 | 0.005 |  |
|  |  | cg07707031 | 3'UTR |  |  |  |  |  | HIV infection |
|  |  | cg00333483 | NSF |  |  |  |  |  | None |
| *FOXJ3* | Chr1:42801728-42801761 |  |  | -0.020 | 0.004 | -5.734 | 9.805e-09 | 0.008 |  |
|  |  | cg12375158 | TSS1500; TSS200 |  |  |  |  |  | *SETD1B*-related syndrome |
|  |  | cg04049626 | NSF |  |  |  |  |  | *SETD1B*-related syndrome |
|  |  | cg18174678 | TSS1500 |  |  |  |  |  | Fetal vs adult liver; smoking status; maternal smoking; gestational age; *SETD1B*-related syndrome; Gulf War illness |
| *SLC39A13* | Chr11:47430791-47430812 |  |  | -0.014 | 0.003 | -5.693 | 1.250e-08 | 0.010 |  |
|  |  | cg05778943 | 5'UTR |  |  |  |  |  | None |
|  |  | cg19668951 | 5'UTR |  |  |  |  |  | Fetal vs adult liver; gestational age; sex; multiple sclerosis |
| *TPPP* | Chr5:672845-672910 |  |  | 0.054 | 0.009 | 5.685 | 1.305e-08 | 0.011 |  |
|  |  | cg22879098 | Body |  |  |  |  |  | Fetal vs adult liver; maternal smoking |
|  |  | cg24082121 | Body |  |  |  |  |  | Fetal vs adult liver; maternal smoking; aging |
|  |  | cg10986412 | Body |  |  |  |  |  | Maternal smoking; fruit consumption; aging |
| *LINC01529* | Chr19:36288702-36288895 |  |  | 0.030 | 0.005 | 5.577 | 2.450e-08 | 0.020 |  |
|  |  | cg02456406 | NSF |  |  |  |  |  | Rheumatoid arthritis |
|  |  | cg14624451 | Body |  |  |  |  |  | None |
|  |  | cg11791444 | Body |  |  |  |  |  | None |
|  |  | cg09182728 | TSS200 |  |  |  |  |  | None |
| *C5orf63* | Chr5:126408756-126408806 |  |  | -0.052 | 0.009 | -5.535 | 3.115e-08 | 0.026 |  |
|  |  | cg14340928^7^ | 5'UTR |  |  |  |  |  | Fetal vs adult liver |
|  |  | cg17848407^7^ | 5'UTR |  |  |  |  |  | None |
| *HCCA2* | Chr11:1769382-1769462 |  |  | 0.036 | 0.007 | 5.465 | 4.632e-08 | 0.038 |  |
|  |  | cg08269485 | Body; TSS200 |  |  |  |  |  | Early childhood development; prostate cancer |
|  |  | cg03846408 | Body; TSS200 |  |  |  |  |  | Early childhood development; prostate cancer |
|  |  | cg08966208 | Body; TSS200 |  |  |  |  |  | Early childhood development; preterm birth; prostate cancer |
|  |  | cg02916272 | Body; TSS200 |  |  |  |  |  | Early childhood development; gestational age; preterm birth; prostate cancer |
|  |  | cg20272155 | Body; TSS200 |  |  |  |  |  | Early childhood development; gestational age |
| *OXT* | Chr20:3052058-3052262 |  |  | -0.030 | 0.005 | -5.452 | 4.973e-08 | 0.041 |  |
|  |  | cg19776589 | TSS1500 |  |  |  |  |  | None |
|  |  | cg07747220 | TSS200 |  |  |  |  |  | Early childhood development; gestational age; aging; childhood stress; multiple sclerosis; preterm birth; antiphospholipid syndrome; neurodevelopmental presentations and congenital anomalies; *SETD1B*-related syndrome |
|  |  | cg16887334 | TSS200 |  |  |  |  |  | Aging; sex; childhood stress; short-term diesel exhaust inhalation |
|  |  | cg13285174 | TSS200 |  |  |  |  |  | Early childhood development; gestational age; aging; cleft palate cs cleft lip; HIV infection; childhood stress; multiple sclerosis; preterm birth; Alzheimer’s disease; *SETD1B-*related syndrome |
|  |  | cg26267561 | TSS200 |  |  |  |  |  | Gestational age; cleft palate vs cleft lip; aging; childhood stress multiple sclerosis; preterm birth; epithelial ovarian cancer; *SETD1B*-related syndrome; Gulf War illness |
|  |  | cg01644611 | TSS200 |  |  |  |  |  | Early childhood development; gestational age; cleft palate vs cleft lip; aging; childhood stress; multiple sclerosis; preterm birth; neurodevelopmental presentations and congenital anomalies |
|  |  | cg13725599 | TSS200 |  |  |  |  |  | Early childhood development; gestational age  cleft palate vs cleft lip; aging; childhood stress |
| *ZNF595* | Chr4:53010-53109 |  |  | 0.018 | 0.003 | 5.424 | 5.830e-08 | 0.048 |  |
|  |  | cg01419539 | TSS1500 |  |  |  |  |  | Early childhood development; smoking status; aging; alcohol consumption |
|  |  | cg07697276 | NSF |  |  |  |  |  | Early childhood development; alcohol consumption |
|  |  | cg26385085 | TSS200 |  |  |  |  |  | Early childhood development |
|  |  |  |  |  |  |  |  |  |  |

^1^ Identified using the GENECODE database; ^2^ identified using the Human Genome 19 (HG19) build from the Genome Reference Consortium; ^3^ identified using the University of California Santa Cruz (UCSC) Genomic Institute/Genome Browser; ^4^ multiple listings indicate splice variants; ^5^ identified using the Medical Research Council Integrative Epidemiology Unit (MRC-IEU) catalog of epigenome-wide association studies^49^ and the China National Center for Bioinformation National Genomics Data Center epigenome-wide association studies (EWAS) atlas^50^; ^6^ CpG sites located in a region not attributed to a gene, the gene closest to the CpG site is provided; ^7^ The site is associated with a promoter region - identified using the Methylation Consortium project.

Abbreviations: Standard error (SE); adjusted (Adj); not applicable (NA); solute carrier family 16 member 9 *(SLC16A9)*; not specified (NSF); interferon regulatory factor 4 *(IRF4)*; fasciculation and elogation protein zeta 1 *(FEZ1)*; 5’ untranslated region (5’UTR); glutamate-cysteine ligase catalytic subunit (*GCLC);* brain-enriched guanylate kinase-associated protein *(BEGAIN)*; transcription start site 1500 (TSS1500); South shore (S_Shore); CRTC3 antisense RNA 1 (*CRTC3-AS1)*; long intergenic non-protein coding RNA 1006 *(LINC01006);* erythrocyte membrane protein band 4.1 Like 1 *(EPB41L1)*; 3’ untranslated region (3’UTR); adenylate cyclase activating polypeptide 1 *(ADCYAP1)*; transcription start site 200 (TSS200); brain-specific serine/threonine-protein kinase 2 *(BRSK2)*; microRNA 4290 *(MIR4290)*; ubiquitin specific peptidase 49 *(USP49)*; docking protein 5 *(DOK5)*; methylmalonyl-CoA epimerase *(MCEE)*; catenin alpha 3 *(CTNNA3)*; novel transcript antisense to purine rich element binding protein B *(AC004854.2)*; PAR-3 family cell polarity regulator (*PARD3)*; North shelf (N_Shelf); transmembrane protein 52B *(TMEM52B)*; TRNA methyltransferase O (*TRMO);* leucine-rich repeat-containing protein 34 *(LRRC34)*; phospholipase D family member 6 *(PLD6)*; North shore (N_Shore); solute carrier family 38 member 11 *(SLC38A11)*; coiled-coil and C2 domain-containing protein 2A *(CC2D2A)*; endothelial cell specific molecule 1 *(ESM1)*; sorbin and SH3 domain-containing protein 2 *(SORBS2)*; ankyrin repeat domain-containing protein 33 *(ANKRD33)*; collagen type XVIII alpha 1 chain *(COL18A1)*; spondin 1 *(SPON1)*; inositol polyphosphate-5-phosphatase A *(INPP5A)*; chromosome 21 open reading frame 62 *(C21orf62)*; thrombospondin type laminin G domain and EAR repeats antisense RNA 2 *(TSPEAR-AS2)*, microRNA 125b-1 *(MIR125B1)*; chromosome 5 open reading frame 66 antisense RNA *(C5orf66AS1);* fascin actin-bundling protein 2 *(FSCN2)*; forkhead box J3 *(FOXJ3)*; solute carrier family 39 member 13 *(SLC39A13)*; tubulin polymerization promoting protein *(TPPP)*; long intergenic non protein coding RNA 1529 *(LINC01529)*; chromosome 5 open reading frame 63 *(C5orf63)*; hepatocellular carcinoma-associated gene 2 *(HCCA2)*, oxytocin/neurophysin I prepropeptide *(OXT)*; and zinc finger protein 595 *(ZNF595)*

Supplementary Table 4: *Genomic coordinates of the BRSK2 CpG sites investigated longitudinally*

| Coordinates | Sequence |
| --- | --- |
|  |  |
| chr11:1463561 | TGATTGGCTGCCTATGACATCACCAGGCTGGGCTGCTATTGG |
| chr11:1463603 | CCCTTATGTGTGATTGG**CG**TTTGGAGAGGCAGTGGGCTCTG  CpG3 |
| chr11:1463644 | GGCAGGGGGTCTCCAGGG**CG**GGGAGG**CG**CTCAAGGCAGA  CpG4 CpG5 |
| chr11:1463683 | GACTGGCCCTGTTCAGCCTCACCACCCTCCTCCCCAGCCACA |
| chr11:1463725 | GGGATCTGAAACCTGAAAACCTCCTGCTGGACGAGAAGAAC |

Genomic coordinates are based on the Human Genome Build 37 (GRCh37/hg19).

Supplementary Table 5: *Univariate relationship between baseline confounding/covarying factors, PTSD, BRSK2 and ADCYAP1 methylation in the discovery/validation sample*

|  | Age  baseline | | HIV status (negative vs positive) | | BMI  baseline | | Smoker  (no vs yes) | | Childhood trauma  baseline | | Lifetime trauma  baseline | | Alcohol use  baseline | | Depression baseline | | Medication use (no vs yes) | |
| --- | --- | --- | --- | --- | --- | --- | --- | --- | --- | --- | --- | --- | --- | --- | --- | --- | --- | --- |
|  | *r/z* | *p* | *z/x^2^* | *p* | *r/z* | *p* | *z/x^2^* | *p* | *r/z* | *p* | *r/z* | *p* | *r/z* | *p* | *r/z* | *p* | *z/x^2^* | *p* |
|  |  |  |  |  |  |  |  |  |  |  |  |  |  |  |  |  |  |  |
| PTSD status (3-months) | -1.05 | .296 | 0.02 | .900 | -1.11 | .268 | 0.18 | .672 | -1.66 | .098 | -1.86 | .063 | -0.85 | .394 | -1.36 | .173 | 0.17 | .680 |
|  |  |  |  |  |  |  |  |  |  |  |  |  |  |  |  |  |  |  |
| *BRSK2* CpG3 (3-months) | 0.02 | .881 | -0.44 | .657 | -0.04 | .810 | -0.07 | .944 | -0.16 | .282 | -0.16 | .301 | 0.02 | .889 | 0.17 | .259 | -0.56 | .573 |
| *BRSK2* CpG4 (3-months) | -0.07 | .650 | -0.17 | .868 | -0.10 | .516 | -0.23 | .818 | -0.14 | .354 | -0.19 | .210 | 0.12 | .443 | 0.17 | .263 | -0.82 | .935 |
| *BRSK2* CpG5 (3-months) | 0.01 | .946 | -0.34 | .731 | -0.10 | .516 | -0.12 | .902 | -0.10 | .527 | -0.12 | .429 | 0.10 | .505 | 0.19 | .208 | -0.52 | .606 |
|  |  |  |  |  |  |  |  |  |  |  |  |  |  |  |  |  |  |  |
| *ADCYAP1* CpG5.6 (3-months) | -0.15 | .341 | -1.47 | .141 | -0.13 | .400 | -1.32 | .188 | 0.17 | .273 | 0.07 | .648 | 0.26 | .083 | 0.10 | .512 | -0.22 | .823 |
|  |  |  |  |  |  |  |  |  |  |  |  |  |  |  |  |  |  |  |

Abbreviations:

Posttraumatic stress disorder (PTSD), brain-specific serine/threonine-protein kinase 2 (*BRSK2*), adenylate cyclase activating polypeptide 1 (*ADCYAP1)*

Supplementary Table 6: *Univariate relationship between baseline confounding/covarying factors, PTSD, BRSK2 and ADCYAP1 methylation in the replication sample*

|  | Age  baseline | | HIV status (negative vs positive) | | BMI  baseline | | Smoker  (no vs yes) | | Childhood trauma  baseline | | Lifetime trauma  baseline | | Alcohol use  baseline | | Depression baseline | | Medication use (no vs yes) | |
| --- | --- | --- | --- | --- | --- | --- | --- | --- | --- | --- | --- | --- | --- | --- | --- | --- | --- | --- |
|  | *r/z* | *p* | *z/x^2^* | *p* | *r/z* | *p* | *z/x^2^* | *p* | *r/z* | *p* | *r/z* | *p* | *r/z* | *p* | *r/z* | *p* | *z/x^2^* | *p* |
|  |  |  |  |  |  |  |  |  |  |  |  |  |  |  |  |  |  |  |
| PTSD status (3-months) | -0.50 | .616 | 0.57 | .451 | -0.33 | .745 | 0.17 | .899 | -1.04 | .299 | -2.47 | .014* | -1.04 | .299 | -1.45 | .149 | 0.62 | .433 |
|  |  |  |  |  |  |  |  |  |  |  |  |  |  |  |  |  |  |  |
| *BRSK2* CpG3 (3-months) | -0.04 | .769 | -0.61 | .545 | -0.05 | .725 | -0.14 | .989 | -0.22 | .133 | -0.14 | .329 | 0.15 | .302 | 0.08 | .587 | -0.96 | .339 |
| *BRSK2* CpG4 (3-months) | -0.06 | .702 | -1.11 | .267 | 0.13 | .360 | -0.82 | .415 | 0.14 | .324 | -0.14 | .349 | 0.10 | .497 | 0.17 | .240 | -1.42 | .156 |
| *BRSK2* CpG5 (3-months) | -0.08 | .571 | -1.23 | .220 | 0.05 | .726 | -0.67 | .501 | -0.10 | .495 | -0.15 | .305 | 0.03 | .836 | 0.08 | .579 | -1.67 | .094 |
|  |  |  |  |  |  |  |  |  |  |  |  |  |  |  |  |  |  |  |
| *ADCYAP1* CpG5.6 (3-months) | 0.04 | .785 | -1.85 | .064 | 0.09 | .557 | -0.54 | .586 | 0.09 | .551 | 0.08 | .576 | -0.24 | .095 | 0.04 | .796 | -1.43 | .154 |
|  |  |  |  |  |  |  |  |  |  |  |  |  |  |  |  |  |  |  |

Abbreviations:

Posttraumatic stress disorder (PTSD), brain-specific serine/threonine-protein kinase 2 (*BRSK2*), adenylate cyclase activating polypeptide 1 (*ADCYAP1)*

Supplementary Table 7: *Validation of the BRSK2 and ADCYAP1 findings resulting from the epigenome-wide association study in the discovery sample*

| Model |  | *β* | SE | Wald’s χ^2^ | *p* | OR | Lower | Upper |
| --- | --- | --- | --- | --- | --- | --- | --- | --- |
|  |  |  |  |  |  |  |  |  |
|  | ***BRSK2* CpG3 methylation** |  |  |  |  |  |  |  |
|  |  |  |  |  |  |  |  |  |
| 1A | CpG3 (3-months) | -0.04 | 0.02 | 3.84 | .050 | 0.96 | 0.92 | 1.00 |
|  |  |  |  |  |  |  |  |  |
| 1B | CpG3 (3-months) | -0.04 | 0.02 | 3.20 | .074 | 0.96 | 0.92 | 1.00 |
|  | Lifetime trauma (baseline) | 0.11 | 0.09 | 1.54 | .215 | 1.11 | 0.94 | 1.31 |
|  |  |  |  |  |  |  |  |  |
|  | ***BRSK2* CpG4 methylation** |  |  |  |  |  |  |  |
|  |  |  |  |  |  |  |  |  |
| 2A | CpG4 (3-months) | -0.04 | 0.02 | 3.78 | .052 | 0.96 | 0.92 | 1.00 |
|  |  |  |  |  |  |  |  |  |
| 2B | CpG4 (3-months) | -0.04 | 0.02 | 3.25 | .072 | 0.96 | 0.92 | 1.00 |
|  | Lifetime trauma (baseline) | 0.11 | 0.08 | 1.66 | .198 | 1.11 | 0.95 | 1.31 |
|  |  |  |  |  |  |  |  |  |
|  | ***BRSK2* CpG5 methylation** |  |  |  |  |  |  |  |
|  |  |  |  |  |  |  |  |  |
| 3A | CpG5 (3-months) | -0.04 | 0.02 | 3.90 | .048* | 0.96 | .928 | 1.00 |
|  |  |  |  |  |  |  |  |  |
| 3B | CpG5 (3-months) | -0.04 | 0.02 | 3.32 | .069 | 0.97 | 0.93 | 1.00 |
|  | Lifetime trauma (baseline) | 0.11 | 0.09 | 1.58 | 0.21 | 1.11 | 0.94 | 1.32 |
|  |  |  |  |  |  |  |  |  |
|  | ***ADCYAP1* CpG1&2 methylation** |  |  |  |  |  |  |  |
|  |  |  |  |  |  |  |  |  |
| 4A | *ADCYAP1* CpG5.6 (3-months) | -0.09 | 0.10 | 0.76 | .382 | 0.92 | 0.76 | 1.11 |
|  |  |  |  |  |  |  |  |  |
| 4B | *ADCYAP1* CpG5.6 (3-months) | -0.13 | 0.11 | 1.25 | .263 | 0.88 | 0.71 | 1.10 |
|  | Lifetime trauma (baseline) | 0.14 | 0.08 | 2.66 | .103 | 1.15 | 0.97 | 1.35 |
|  |  |  |  |  |  |  |  |  |

*p < 0.05

Abbreviations: standard error (SE); odds ratio (OR), brain-specific serine/threonine-protein kinase 2 *(BRSK2)*; adenylate cyclase activating polypeptide 1 *(ADCYAP1)*.

Supplementary Table 8: *Replication of the BRSK2 and ADCYAP1 findings resulting from the epigenome-wide association study in the discovery sample*

| Model |  | *β* | SE | Wald’s χ^2^ | *p* | OR | Lower | Upper |
| --- | --- | --- | --- | --- | --- | --- | --- | --- |
|  |  |  |  |  |  |  |  |  |
|  | ***BRSK2* CpG3 methylation** |  |  |  |  |  |  |  |
|  |  |  |  |  |  |  |  |  |
| 1A | CpG3 (3-months) | -0.00 | 0.02 | 0.02 | .889 | 1.00 | 0.96 | 1.04 |
|  |  |  |  |  |  |  |  |  |
| 1B | CpG3 (3-months) | -0.01 | 0.02 | 0.11 | .736 | 0.99 | 0.95 | 1.04 |
|  | Lifetime trauma (baseline) | -0.12 | 0.14 | 0.84 | .360 | 0.88 | 0.68 | 1.15 |
|  |  |  |  |  |  |  |  |  |
|  | ***BRSK2* CpG4 methylation** |  |  |  |  |  |  |  |
|  |  |  |  |  |  |  |  |  |
| 2A | CpG4 (3-months) | -0.01 | 0.02 | 0.19 | .667 | 0.99 | 0.95 | 1.04 |
|  |  |  |  |  |  |  |  |  |
| 2B | CpG4 (3-months) | -0.01 | 0.02 | 0.32 | .570 | 0.99 | 0.94 | 1.03 |
|  | Lifetime trauma (baseline) | -0.13 | 0.14 | 0.87 | .350 | 0.88 | 0.68 | 1.15 |
|  |  |  |  |  |  |  |  |  |
|  | ***BRSK2* CpG5 methylation** |  |  |  |  |  |  |  |
|  |  |  |  |  |  |  |  |  |
| 3A | CpG5 (3-months) | 0.00 | 0.02 | 0.03 | .866 | 1.00 | 0.97 | 1.04 |
|  |  |  |  |  |  |  |  |  |
| 3B | CpG5 (3-months) | 0.00 | 0.02 | 0.01 | .944 | 1.00 | 0.96 | 1.04 |
|  | Lifetime trauma (baseline) | -0.13 | 0.14 | 0.88 | .347 | 0.88 | 0.68 | 1.15 |
|  |  |  |  |  |  |  |  |  |
|  | ***ADCYAP1* CpG1&2 methylation** |  |  |  |  |  |  |  |
|  |  |  |  |  |  |  |  |  |
| 4A | *ADCYAP1* CpG5.6 (3-months) | -0.06 | 0.13 | 0.22 | .639 | 0.94 | 0.73 | 1.21 |
|  |  |  |  |  |  |  |  |  |
| 4B | *ADCYAP1* CpG5.6 (3-months) | -0.5 | 0.13 | 0.15 | .700 | 0.95 | 0.74 | 1.22 |
|  | Lifetime trauma (baseline) | -0.12 | 0.13 | 0.83 | .362 | 0.89 | 0.68 | 1.15 |
|  |  |  |  |  |  |  |  |  |

Abbreviations: standard error (SE); odds ratio (OR), brain-specific serine/threonine-protein kinase 2 *(BRSK2)*; adenylate cyclase activating polypeptide 1 *(ADCYAP1)*.

Supplementary Table 9: Genomic coordinates of the *ADCYAP1* CpG sites investigated longitudinally

| Coordinates | Sequence |
| --- | --- |
|  |  |
| chr18:905159 | GTCTGGCTAGTTATTGGG**CGCCG**GGTAGATGCATATATAT  CpG1 CpG2 |
| chr18:905199 | ATATTTTTTTCTAACTATAGCAAGCAAGAAGTGGCAGGG |

Genomic coordinates are based on the Human Genome Build 37 (GRCh37/hg19).

Supplementary Table 10*: Comparison of BRSK2 methylation levels obtained from the MethylationEPIC array and the EpiTYPER analysis*

|  | *BRSK2* CpG3 EpiTYPER | *BRSK2* CpG4 EpiTYPER | *BRSK2* CpG5 EpiTYPER | *BRSK2* CpG3 EWAS | *BRSK2* CpG4 EWAS | *BRSK2* CpG5 EWAS |
| --- | --- | --- | --- | --- | --- | --- |
| *BRSK2* CpG3  EpiTYPER | 1.000 |  |  |  |  |  |
| *BRSK2* CpG4  EpiTYPER | .899** | 1.000 |  |  |  |  |
| *BRSK2* CpG5  EpiTYPER | .908** | .901** | 1.000 |  |  |  |
| *BRSK2* CpG3  EPIC array | .881** | .912** | .859** | 1.000 |  |  |
| *BRSK2* CpG4  EPIC array | .873** | .900** | .843** | .950** | 1.000 |  |
| *BRSK2* CpG5  EPIC array | .861** | .889** | .831** | .956** | .967** | 1.000 |
|  |  |  |  |  |  |  |

*p < .05, **p < .01

Abbreviations: brain-specific serine/threonine-protein kinase 2 *(BRSK2)*; epigenome-wide association study (EWAS)

Supplementary Table 11: *Comparison of ADCYAP1 methylation levels obtained from the MethylationEPIC array and the EpiTYPER analysis*

|  | *ADCYAP1* CpG1&2  EpiTYPER | *ADCYAP1* CpG1&2  EWAS |
| --- | --- | --- |
| *ADCYAP1* CpG1&2  EpiTYPER | 1.000 |  |
| *ADCYAP1* CpG1&2  EPIC array | .254 | 1.000 |
|  |  |  |

*p < .05, **p < .01

Abbreviations: adenylate cyclase activating polypeptide 1 (ADCYAP1), epigenome-wide association study (EWAS)

Supplementary Table 12: *Summary of genes and CpG sites investigated in candidate gene studies alongside findings in the current epigenome-wide association study*

| **Gene name^1^** | **Genomic position^2^** | **Location in gene^3,4^** | **Current EWAS** | | | **Prior studies** | | | |
| --- | --- | --- | --- | --- | --- | --- | --- | --- | --- |
|  |  |  | **Finding** | ***p*-value** | **Study Identifier** | **Finding** | ***p*-value** | **Study Identifier** | **Reference** |
|  |  |  |  |  |  |  |  |  |  |
| *ADCYAP1* |  |  |  |  |  |  |  |  |  |
|  | chr18:905101 | TSS200; TSS1500; 5’UTR | ↑ Methylation in group with PTSD | *p*=.034 | cg17059658 |  |  |  |  |
|  | chr18:905127 | TSS200; TSS1500; 5’UTR | ↑ Methylation in group with PTSD | *p*=.006 | cg15194943 |  |  |  |  |
|  | chr18:905177 | TSS200; TSS1500; 5’UTR | ↑ Methylation in group with PTSD | *p*=.00008 | cg22388954 |  |  |  |  |
|  | chr18:905180 | TSS200; TSS1500; 5’UTR | ↑ Methylation in group with PTSD | *p*=.0008 | cg11773720 |  |  |  |  |
|  | chr18:905245 | TSS200;  5’UTR | ↑ Methylation in group with PTSD | *p*=.044 | cg09172003 |  |  |  |  |
|  | chr18:905450 | NSF |  |  |  | Not significant | *p*>.05 | cg07376535 | Ressler et al., 2011^52^ |
|  |  |  |  |  |  |  |  |  |  |
| *ADCYAP1R1* |  |  |  |  |  |  |  |  |  |
|  | chr7:31091718 | TSS1500 |  |  |  | ↑ Methylation ↑ PTSD symptom severity | *p*<.0005* | cg27076139 | Ressler et al., 2011^52^ |
|  | chr7:31092854 | 5’UTR | ↓ Methylation in group with PTSD | *p*=.012 | cg11218385 |  |  |  |  |
|  |  |  |  |  |  |  |  |  |  |
| *AIM2* |  |  |  |  |  |  |  |  |  |
|  | chr1:159046973 | TSS1500 |  |  |  | ↓ Methylation ↑ PTSD symptom severity | *p*=.009* | cg10636246 | Miller et al., 2018^53^ |
|  |  |  |  |  |  |  |  |  |  |
| *BDNF* |  |  |  |  |  |  |  |  |  |
|  | chr11:27681475 | TSS1500; 3’UTR; 5’UTR | ↑ Methylation in group with PTSD | *p*=.017 | cg07238832 |  |  |  |  |
|  | chr11:27722889 | TSS1500; 3’UTR; 5’UTR | ↓ Methylation in group with PTSD | *p*=.029 | cg04672351 |  |  |  |  |
|  | chr11:27732958 | 3’UTR; 5’UTR | ↓ Methylation in group with PTSD | *p*=.029 | cg11806762 |  |  |  |  |
|  | chr11:27744022^6^ | Exon IV |  |  |  | Not significant | *p*>.05 | CpG4 | Moser et al., 2015^54^ |
|  | chr11:27744049 | TSS1500; 3’UTR | ↓ Methylation in group with PTSD | *p*=.021 | cg15462887 |  |  |  |  |
|  | chr11:27744279^6^ | Exon I |  |  |  | ↑ Methylation in group with PTSD | *p*=.009* | CpG4 | Kim et al., 2017^55^ |
|  | chr11:27744286^6^ | Exon I |  |  |  | ↑ Methylation in group with PTSD | *p*=.021* | CpG3 | Kim et al., 2017^55^ |
|  | chr11:27744290^6^ | Exon I |  |  |  | ↑ Methylation in group with PTSD | *p*=.039* | CpG2 | Kim et al., 2017^55^ |
|  | chr11:27744292^6^ | Exon I |  |  |  | ↑ Methylation in group with PTSD | *p*=.053 | CpG1 | Kim et al., 2017^55^ |
|  | chr11:27744963^6^ | Exon IV |  |  |  | Not significant | *p*>.05 | CpG1 | Moser et al., 2015^54^ |
|  | chr11:27744971^6^ | Exon IV |  |  |  | Not significant | *p*>.05 | CpG2 | Moser et al., 2015^54^ |
|  | chr11:27744975^6^ | Exon IV |  |  |  | Not significant | *p*>.05 | CpG3 | Moser et al., 2015^54^ |
|  |  |  |  |  |  |  |  |  |  |
| *COMT* |  |  |  |  |  |  |  |  |  |
|  | chr22:19950040 | NSF |  |  |  | ↑ Methylation in group with PTSD | *p*<.01* | cg23601416 | Norrholm et al., 2013^56^ |
|  | chr22:19956281 | 3’UTR | ↓ Methylation in group with PTSD | *p*=.040 | cg19930203 |  |  |  |  |
|  | chr22:19938096 | TSS1500; 5’UTR | ↑ Methylation in group with PTSD | *p*=.002 | cg09926649 |  |  |  |  |
|  | chr22:19928616 | TSS1500; 3’UTR; 5’UTR | ↑ Methylation in group with PTSD | *p*=.015 | cg27399558 |  |  |  |  |
|  | chr22:19928667 | TSS1500; 3’UTR; 5’UTR | ↑ Methylation in group with PTSD | *p*=.044 | cg11712482 |  |  |  |  |
|  | chr22:19928740 | TSS1500; 3’UTR; 5’UTR | ↑ Methylation in group with PTSD | *p*=.039 | cg24547396 |  |  |  |  |
|  | chr22:19928445 | TSS1500; 3’UTR; 5’UTR | ↑ Methylation in group with PTSD | *p*=.0004 | cg15926585 |  |  |  |  |
|  | chr22:19949585 | TSS1500; 5’UTR | ↑ Methylation in group with PTSD | *p*=.028 | cg21905167 |  |  |  |  |
|  |  |  |  |  |  |  |  |  |  |
| *FKBP5* |  |  |  |  |  |  |  |  |  |
|  | chr6:35558386^6^ | Intron 7; TSS |  |  |  | Not significant | *p*>.05 | CpG  35558386 | Bishop et al., 2018^57^ |
|  |  |  |  |  |  | Not significant | *p*>.05 | Bin1-CpG1 | Yehuda et al., 2016^58^ |
|  | chr6:35558438^6^ | Intron 7; TSS |  |  |  | Not significant | *p*>.05 | CpG  35558438 | Bishop et al., 2018^57^ |
|  |  |  |  |  |  | Not significant | *p*>.05 | Bin1-CpG2 | Yehuda et al., 2016^58^ |
|  | chr6:35558488 | Intron 7; TSS |  |  |  | Not significant | *p*>.05 | CpG35558488 | Bishop et al., 2018^57^ |
|  |  |  |  |  |  | Not significant | *p*>.05 | Bin2-CpG3 | Yehuda et al, 2016^58^ |
|  |  |  |  |  |  | ↑ Methylation in group with PTSD | *p*=.037* | CpG1 | Kang et al., 2019^59^ |
|  | chr6:35558513^6^ | Intron 7; TSS |  |  |  | ↑ Methylation in MBSR non-responders post-treatment | *p*=.029* | CpG35558513 | Bishop et al., 2018^57^ |
|  |  |  |  |  |  | Not significant | *p*>.05 | Bin2-CpG4-GRE | Yehuda et al., 2016^58^ |
|  |  |  |  |  |  | ↑ Methylation in group with PTSD | *p*=.037* | CpG2 | Kang et al., 2019^59^ |
|  | chr6:35558566^6^ | Intron 7; TSS |  |  |  | Not significant | *p*>.05 | CpG35558566 | Bishop et al., 2018^57^ |
|  |  |  |  |  |  | Not significant | *p*>.05 | Bin2-CpG5 | Yehuda et al., 2016^58^ |
|  | chr6:35558710^6^ | Intron 7; TSS |  |  |  | Not significant | *p*>.05 | CpG35558710 | Bishop et al., 2018^57^ |
|  |  |  |  |  |  | ↑ Methylation in group with PTSD | *p*=.046* | Bin3-CpG6 | Yehuda et al., 2016^58^ |
|  | chr6:35558721^6^ | Intron 7; TSS |  |  |  | Not significant | *p*>.05 | CpG35558721 | Bishop et al., 2018^57^ |
|  | chr6:35656916-35656633 | exon1 |  |  |  | ↑ Methylation (across 38 CpG sites) ↑ PTSD symptom severity | *p*=.044* | CpG1-CpG38 | Yehuda et al., 2016^58^ |
|  | chr6:35681420 | 5’UTR | ↓ Methylation in group with PTSD | *p*=.046 | cg24295963 |  |  |  |  |
|  |  |  |  |  |  |  |  |  |  |
| *HTR3A* |  |  |  |  |  |  |  |  |  |
|  | chr11:113827917^6^ | GRE |  |  |  | Not significant | *p*>.05 | CpG1_I | Schechter et al., 2017^60^ |
|  | chr11:113844529^6^ | TSS |  |  |  | Not significant | *p*>.05 | CpG4_II | Schechter et al., 2017^60^ |
|  | chr11:113844828 | TSS |  |  |  | Not significant | *p*>.05 | CpG1_II | Schechter et al., 2017^60^ |
|  | chr11:113844864^6^ | TSS |  |  |  | Not significant | *p*>.05 | CpG2_II | Schechter et al., 2017^60^ |
|  | chr11:113844942^6^ | TSS |  |  |  | Not significant | *p*>.05 | CpG3_II | Schechter et al., 2017^60^ |
|  | chr11:113845939 | 5'UTR |  |  |  | Not significant | *p*>.05 | CpG1_III | Schechter et al., 2017^60^ |
|  | chr11:113846004 | 5'UTR |  |  |  | ↓ Methylation in group with PTSD | *p*=.009* | CpG2_III | Schechter et al., 2017^60^ |
|  | chr11:113846004 | TSS200; 5’UTR | ↓ Methylation in group with PTSD | *p*=.028 | cg20621129 |  |  |  |  |
|  | chr11:113846017 | 5'UTR |  |  |  | Not significant | *p*>.05 | CpG3_III | Schechter et al., 2017^60^ |
|  | chr11:113846044^6^ | Coding Sequence |  |  |  | ↑ Methylation in group with PTSD | *p*=.002* | CpG4_III | Schechter et al., 2017^60^ |
|  | chr11:113846070^6^ | Coding Sequence |  |  |  | ↑ Methylation in group with PTSD | *p*=.004* | CpG5_III | Schechter et al., 2017^60^ |
|  | chr11:113846077^6^ | Coding Sequence |  |  |  | Not significant | *p*>.05 | CpG6_III | Schechter et al., 2017^60^ |
|  | chr11:113860607 | 3’UTR | ↑ Methylation in group with PTSD | *p*=.036 | cg20178075 |  |  |  |  |
|  |  |  |  |  |  |  |  |  |  |
| *IL12B* |  |  |  |  |  |  |  |  |  |
|  | chr5:159314783-159330473 | TSS |  |  |  | ↓ Methylation in group with PTSD | NSF | NA | Bam et al., 2016^61^ |
|  |  |  |  |  |  |  |  |  |  |
| *MAN2C1* |  |  |  |  |  |  |  |  |  |
|  | chr15:75660215 | TSS1500; 3’UTR | ↓ Methylation in group with PTSD | *p*=.024 | cg05432169 |  |  |  |  |
|  | chr15:75661449 | NSF |  |  |  | ↑ Methylation x ↑ trauma load in group with PTSD | *p*=.04* | cg04008455 | Uddin et al., 2011^62^ |
|  | chr15:75661532 | TSS1500; 5’UTR | ↑ Methylation in group with PTSD | *p*=.029 | cg04095413 |  |  |  |  |
|  |  |  |  |  |  |  |  |  |  |
| *MAOA* |  |  |  |  |  |  |  |  |  |
|  | chrX:43515349 | TSS200 | ↑ Methylation in group with PTSD | *p*=.048 | cg19441691 |  |  |  |  |
|  | chrX:43515613^6^ | Exon1/Intron1 |  |  |  | Not significant | *p*>.05 | CpG1 | Ziegler et al., 2018^63^ |
|  | chrX:43515624^6^ | Exon1/Intron1 |  |  |  | Not significant | *p*>.05 | CpG2 | Ziegler et al., 2018^63^ |
|  | chrX:43515658^6^ | Exon1/Intron1 |  |  |  | ↑ Methylation in group with PTSD | *p*=.029* | CpG3 | Ziegler et al., 2018^63^ |
|  | chrX:43515665^6^ | Exon1/Intron1 |  |  |  | Not significant | *p*>.05 | CpG4 | Ziegler et al., 2018^63^ |
|  | chrX:43515667^6^ | Exon1/Intron1 |  |  |  | Not significant | *p*>.05 | CpG5 | Ziegler et al., 2018^63^ |
|  | chrX:43515680^6^ | Exon1/Intron1 |  |  |  | Not significant | *p*>.05 | CpG6 | Ziegler et al., 2018^63^ |
|  | chrX:43515683^6^ | Exon1/Intron1 |  |  |  | Not significant | *p*>.05 | CpG7 | Ziegler et al., 2018^63^ |
|  | chrX:43515689^6^ | Exon1/Intron1 |  |  |  | Not significant | *p*>.05 | CpG8 | Ziegler et al., 2018^63^ |
|  | chrX:43515695^6^ | Exon1/Intron1 |  |  |  | Not significant | *p*>.05 | CpG9 | Ziegler et al., 2018^63^ |
|  | chrX:43515724^6^ | Exon1/Intron1 |  |  |  | Not significant | *p*>.05 | CpG10 | Ziegler et al., 2018^63^ |
|  | chrX:43515729^6^ | Exon1/Intron1 |  |  |  | Not significant | *p*>.05 | CpG11 | Ziegler et al., 2018^63^ |
|  | chrX:43515811^6^ | Exon1/Intron1 |  |  |  | ↑ Methylation in group with PTSD | *p*=.011* | CpG12 | Ziegler et al., 2018^63^ |
|  | chrX:43515840^6^ | Exon1/Intron1 |  |  |  | Not significant | *p*>.05 | CpG13 | Ziegler et al., 2018^63^ |
|  |  |  |  |  |  |  |  |  |  |
| *NR3C1* |  |  |  |  |  |  |  |  |  |
|  | chr5:142729377 | 3’UTR; 5’UTR | ↓ Methylation in group with PTSD | *p*=.029 | cg22233604 |  |  |  |  |
|  | chr5:142729913 | 3’UTR; 5’UTR | ↑ Methylation in group with PTSD | *p*=.031 | cg03857453 |  |  |  |  |
|  | chr5:142735238 | 3’UTR; 5’UTR | ↑ Methylation in group with PTSD | *p*=.008 | cg14621978 |  |  |  |  |
|  | chr5:142783095^6^ | 5'UTR, exon 1C |  |  |  | Not significant | *p*>.05 | 1C-CpG54 | Labonte et al., 2014^64^ |
|  | chr5:142783101^6^ | 5'UTR, exon 1C |  |  |  | Not significant | *p*>.05 | 1C-CpG53 | Labonte et al., 2014^64^ |
|  | chr5:142783104^6^ | 5'UTR, exon 1C |  |  |  | Not significant | *p*>.05 | 1C-CpG52 | Labonte et al., 2014^64^ |
|  | chr5:142783112^6^ | 5'UTR, exon 1C |  |  |  | ↑ Methylation in group with PTSD | *p*<.05* | 1C-CpG51 | Labonte et al., 2014^64^ |
|  | chr5:142783120^6^ | 5'UTR, exon 1C |  |  |  | Not significant | *p*>.05 | 1C-CpG50 | Labonte et al., 2014^64^ |
|  | chr5:142783128^6^ | 5'UTR, exon 1C |  |  |  | Not significant | *p*>.05 | 1C-CpG49 | Labonte et al., 2014^64^ |
|  | chr5:142783139^6^ | 5'UTR, exon 1C |  |  |  | Not significant | *p*>.05 | 1C-CpG48 | Labonte et al., 2014^64^ |
|  | chr5:142783145^6^ | 5'UTR, exon 1C |  |  |  | Not significant | *p*>.05 | 1C-CpG47 | Labonte et al., 2014^64^ |
|  | chr5:142783161^6^ | 5'UTR, exon 1C |  |  |  | Not significant | *p*>.05 | 1C-CpG46 | Labonte et al., 2014^64^ |
|  | chr5:142783164^6^ | 5'UTR, exon 1C |  |  |  | Not significant | *p*>.05 | 1C-CpG45 | Labonte et al., 2014^64^ |
|  | chr5:142783167^6^ | 5'UTR, exon 1C |  |  |  | Not significant | *p*>.05 | 1C-CpG44 | Labonte et al., 2014^64^ |
|  | chr5:142783181^6^ | 5'UTR, exon 1C |  |  |  | Not significant | *p*>.05 | 1C-CpG43 | Labonte et al., 2014^64^ |
|  | chr5:142783183^6^ | 5'UTR, exon 1C |  |  |  | Not significant | *p*>.05 | 1C-CpG42 | Labonte et al., 2014^64^ |
|  | chr5:142783189^6^ | 5'UTR, exon 1C |  |  |  | ↓ Methylation in group with PTSD | *p*<.05* | 1C-CpG41 | Labonte et al., 2014^64^ |
|  | chr5:142783191^6^ | 5'UTR, exon 1C |  |  |  | ↓ Methylation in group with PTSD | *p*<.05* | 1C-CpG40 | Labonte et al., 2014^64^ |
|  | chr5:142783204^6^ | 5'UTR, exon 1C |  |  |  | Not significant | *p*>.05 | 1C-CpG39 | Labonte et al., 2014^64^ |
|  | chr5:142783213^6^ | 5'UTR, exon 1C |  |  |  | Not significant | *p*>.05 | 1C-CpG38 | Labonte et al., 2014^64^ |
|  | chr5:142783217^6^ | 5'UTR, exon 1C |  |  |  | Not significant | *p*>.05 | 1C-CpG37 | Labonte et al., 2014^64^ |
|  | chr5:142783221^6^ | 5'UTR, exon 1C |  |  |  | Not significant | *p*>.05 | 1C-CpG36 | Labonte et al., 2014^64^ |
|  | chr5:142783227^6^ | 5'UTR, exon 1C |  |  |  | Not significant | *p*>.05 | 1C-CpG35 | Labonte et al., 2014^64^ |
|  | chr5:142783231^6^ | 5'UTR, exon 1C |  |  |  | Not significant | *p*>.05 | 1C-CpG34 | Labonte et al., 2014^64^ |
|  | chr5:142783238^6^ | 5'UTR, exon 1C |  |  |  | Not significant | *p*>.05 | 1C-CpG33 | Labonte et al., 2014^64^ |
|  | chr5:142783248^6^ | 5'UTR, exon 1C |  |  |  | Not significant | *p*>.05 | 1C-CpG32 | Labonte et al., 2014^64^ |
|  | chr5:142783251^6^ | 5'UTR, exon 1C |  |  |  | Not significant | *p*>.05 | 1C-CpG31 | Labonte et al., 2014^64^ |
|  | chr5:142783256^6^ | 5'UTR, exon 1C |  |  |  | Not significant | *p*>.05 | 1C-CpG30 | Labonte et al., 2014^64^ |
|  | chr5:142783259^6^ | 5'UTR, exon 1C |  |  |  | Not significant | *p*>.05 | 1C-CpG29 | Labonte et al., 2014^64^ |
|  | chr5:142783261^6^ | 5'UTR, exon 1C |  |  |  | Not significant | *p*>.05 | 1C-CpG28 | Labonte et al., 2014^64^ |
|  | chr5:142783271^6^ | 5'UTR, exon 1C |  |  |  | Not significant | *p*>.05 | 1C-CpG27 | Labonte et al., 2014^64^ |
|  | chr5:142783279^6^ | 5'UTR, exon 1C |  |  |  | Not significant | *p*>.05 | 1C-CpG26 | Labonte et al., 2014^64^ |
|  | chr5:142783281^6^ | 5'UTR, exon 1C |  |  |  | Not significant | *p*>.05 | 1C-CpG25 | Labonte et al., 2014^64^ |
|  | chr5:142783298^6^ | 5'UTR, exon 1C |  |  |  | Not significant | *p*>.05 | 1C-CpG24 | Labonte et al., 2014^64^ |
|  | chr5:142783302^6^ | 5'UTR, exon 1C |  |  |  | Not significant | *p*>.05 | 1C-CpG23 | Labonte et al., 2014^64^ |
|  | chr5:142783309^6^ | 5'UTR, exon 1C |  |  |  | Not significant | *p*>.05 | 1C-CpG22 | Labonte et al., 2014^64^ |
|  | chr5:142783313^6^ | 5'UTR, exon 1C |  |  |  | Not significant | *p*>.05 | 1C-CpG21 | Labonte et al., 2014^64^ |
|  | chr5:142783321^6^ | 5'UTR, exon 1C |  |  |  | Not significant | *p*>.05 | 1C-CpG20 | Labonte et al., 2014^64^ |
|  | chr5:142783323^6^ | 5'UTR, exon 1C |  |  |  | Not significant | *p*>.05 | 1C-CpG19 | Labonte et al., 2014^64^ |
|  | chr5:142783325^6^ | 5'UTR, exon 1C |  |  |  | Not significant | *p*>.05 | 1C-CpG18 | Labonte et al., 2014^64^ |
|  | chr5:142783328^6^ | 5'UTR, exon 1C |  |  |  | Not significant | *p*>.05 | 1C-CpG17 | Labonte et al., 2014^64^ |
|  | chr5:142783332^6^ | 5'UTR, exon 1C |  |  |  | Not significant | *p*>.05 | 1C-CpG16 | Labonte et al., 2014^64^ |
|  | chr5:142783334^6^ | 5'UTR, exon 1C |  |  |  | Not significant | *p*>.05 | 1C-CpG15 | Labonte et al., 2014^64^ |
|  | chr5:142783341^6^ | 5'UTR, exon 1C |  |  |  | Not significant | *p*>.05 | 1C-CpG14 | Labonte et al., 2014^64^ |
|  | chr5:142783360^6^ | 5'UTR, exon 1C |  |  |  | Not significant | *p*>.05 | 1C-CpG13 | Labonte et al., 2014^64^ |
|  | chr5:142783379 | 5'UTR, exon 1C |  |  |  | Not significant | *p*>.05 | 1C-CpG12 | Labonte et al., 2014^64^ |
|  | chr5:142783383 | 5'UTR, exon 1C |  |  |  | Not significant | *p*>.05 | 1C-CpG11 | Labonte et al., 2014^64^ |
|  | chr5:142783385 | 5'UTR, exon 1C |  |  |  | Not significant | *p*>.05 | 1C-CpG10 | Labonte et al., 2014^64^ |
|  | chr5:142783400^6^ | 5'UTR, exon 1C |  |  |  | Not significant | *p*>.05 | 1C-CpG9 | Labonte et al., 2014^64^ |
|  | chr5:142783407^6^ | 5'UTR, exon 1C |  |  |  | Not significant | *p*>.05 | 1C-CpG8 | Labonte et al., 2014^64^ |
|  | chr5:142783409^6^ | 5'UTR, exon 1C |  |  |  | Not significant | *p*>.05 | 1C-CpG7 | Labonte et al., 2014^64^ |
|  | chr5:142783411^6^ | 5'UTR, exon 1C |  |  |  | Not significant | *p*>.05 | 1C-CpG6 | Labonte et al., 2014^64^ |
|  | chr5:142783418^6^ | 5'UTR, exon 1C |  |  |  | Not significant | *p*>.05 | 1C-CpG5 | Labonte et al., 2014^64^ |
|  | chr5:142783426^6^ | 5'UTR, exon 1C |  |  |  | Not significant | *p*>.05 | 1C-CpG4 | Labonte et al., 2014^64^ |
|  | chr5:142783432^6^ | 5'UTR, exon 1C |  |  |  | Not significant | *p*>.05 | 1C-CpG3 | Labonte et al., 2014^64^ |
|  | chr5:142783435^6^ | 5'UTR, exon 1C |  |  |  | Not significant | *p*>.05 | 1C-CpG2 | Labonte et al., 2014^64^ |
|  | chr5:142783438^6^ | 5'UTR, exon 1C |  |  |  | Not significant | *p*>.05 | 1C-CpG1 | Labonte et al., 2014^64^ |
|  |  |  |  |  |  | Not significant | *p*>.05 | CpG13 | Schechter et al., 2015^65^ |
|  |  |  |  |  |  | Not significant | *p*>.05 | CpG52 | Schur et al., 2017^66^ |
|  | chr5:142783531^6^ | 5'UTR, exon 1F |  |  |  | Not significant | *p*>.05 | CpG12 | Schechter et al., 2015^65^ |
|  |  |  |  |  |  | Not significant | *p*>.05 | CpG51 | Schur et al., 2017^66^ |
|  | chr5:142783537^6^ | 5'UTR, exon 1F |  |  |  | Not significant | *p*>.05 | CpG11 | Schechter et al., 2015^65^ |
|  |  |  |  |  |  | Not significant | *p*>.05 | CpG50 | Schur et al., 2017^66^ |
|  | chr5:142783540^6^ | 5'UTR, exon 1F |  |  |  | Not significant | *p*>.05 | CpG10 | Schechter et al., 2015^65^ |
|  |  |  |  |  |  | Not significant | *p*>.05 | CpG49 | Schur et al., 2017^66^ |
|  | chr5:142783547^6^ | 5'UTR, exon 1F |  |  |  | Not significant | *p*>.05 | CpG9 | Schechter et al., 2015^65^ |
|  |  |  |  |  |  | Not significant | *p*>.05 | CpG48 | Schur et al., 2017^66^ |
|  | chr5:142783566^6^ | 5'UTR, exon 1F |  |  |  | Not significant | *p*>.05 | CpG8 | Schechter et al., 2015^65^ |
|  |  |  |  |  |  | Not significant | *p*>.05 | CpG8 | Vukojevic et al., 2014^67^ |
|  |  |  |  |  |  | Not significant | *p*>.05 | CpG47 | Schur et al., 2017^66^ |
|  | chr5:142783569 | 5'UTR, exon 1F |  |  |  | Not significant | *p*>.05 | CpG7 | Schechter et al., 2015^65^ |
|  |  |  |  |  |  | Not significant | *p*>.05 | CpG7 | Vukojevic et al., 2014^67^ |
|  |  |  |  |  |  | Not significant | *p*>.05 | CpG46 | Schur et al., 2017^66^ |
|  | chr5:142783584^6^ | 5'UTR, exon 1F |  |  |  | Not significant | *p*>.05 | CpG6 | Schechter et al., 2015^65^ |
|  |  |  |  |  |  | Not significant | *p*>.05 | CpG6 | Vukojevic et al., 2014^67^ |
|  |  |  |  |  |  | Not significant | *p*>.05 | CpG45 | Schur et al., 2017^66^ |
|  | chr5:142783607 | 5'UTR, exon 1F |  |  |  | ↓ Methylation in group with PTSD | *p*=.022* | CpG39 | Yehuda et al., 2013^68^ |
|  |  |  |  |  |  | Not significant | *p*>.05 | CpG5 | Schechter et al., 2015^65^ |
|  |  |  |  |  |  | Not significant | *p*>.05 | CpG26 | Mcnerney et al., 2018^69^ |
|  |  |  |  |  |  | Not significant | *p*>.05 | CpG5 | Vukojevic et al., 2014^67^ |
|  |  |  |  |  |  | Not significant | *p*>.05 | CpG44 | Schur et al., 2017^66^ |
|  | chr5:142783621 | 5'UTR, exon 1F, NGFI-A binding site |  |  |  | Not significant | *p*>.05 | CpG38 | Yehuda et al., 2015^70^ |
|  |  |  |  |  |  | Not significant | *p*>.05 | CpG4 | Schechter et al., 2015^65^ |
|  |  |  |  |  |  | Not significant | *p*>.05 | CpG25 | Mcnerney et al., 2018^69^ |
|  |  |  |  |  |  | Not significant | *p*>.05 | CpG4 | Vukojevic et al., 2014^67^ |
|  |  |  |  |  |  | Not significant | *p*>.05 | CpG43 | Schur et al., 2017^66^ |
|  | chr5:142783627^6^ | 5'UTR, exon 1F, NGFI-A binding site |  |  |  | Not significant | *p*>.05 | CpG37 | Yehuda et al., 2015^70^ |
|  |  |  |  |  |  | Not significant | *p*>.05 | CpG3 | Schechter et al., 2015^65^ |
|  |  |  |  |  |  | Not significant | *p*>.05 | CpG24 | Mcnerney et al., 2018^69^ |
|  |  |  |  |  |  | ↓ Methylation ↑ PTSD symptom severity | *p*<.05* | CpG3 | Vukojevic et al., 2014^67^ |
|  |  |  |  |  |  | Not significant | *p*>.05 | CpG42 | Schur et al., 2017^66^ |
|  | chr5:142783637^6^ | 5'UTR, exon 1F |  |  |  | Not significant | *p*>.05 | CpG36 | Yehuda et al., 2015^70^ |
|  |  |  |  |  |  | Not significant | *p*>.05 | CpG2 | Schechter et al., 2015^65^ |
|  |  |  |  |  |  | Not significant | *p*>.05 | CpG23 | Mcnerney et al., 2018^69^ |
|  |  |  |  |  |  | Not significant | *p*>.05 | CpG2 | Vukojevic et al., 2014^67^ |
|  |  |  |  |  |  | Not significant | *p*>.05 | CpG41 | Schur et al., 2017^66^ |
|  | chr5:142783639 | 5'UTR, exon 1F |  |  |  | Not significant | *p*>.05 | CpG35 | Yehuda et al., 2015^70^ |
|  |  |  |  |  |  | Not significant | *p*>.05 | CpG1 | Schechter et al., 2015^65^ |
|  |  |  |  |  |  | Not significant | *p*>.05 | CpG22 | Mcnerney et al., 2018^69^ |
|  |  |  |  |  |  | Not significant | *p*>.05 | CpG1 | Vukojevic et al., 2014^67^ |
|  |  |  |  |  |  | Not significant | *p*>.05 | CpG40 | Schur et al., 2017^66^ |
|  |  |  |  |  |  |  |  |  |  |
|  | chr5:142783655^6^ | 5'UTR, exon 1F |  |  |  | Not significant | *p*>.05 | CpG34 | Yehuda et al., 2015^70^ |
|  |  |  |  |  |  | Not significant | *p*>.05 | CpG21 | Mcnerney et al., 2018^69^ |
|  |  |  |  |  |  | Not significant | *p*>.05 | CpG39 | Schur et al., 2017^66^ |
|  | chr5:142783663^6^ | 5'UTR, exon 1F |  |  |  | Not significant | *p*>.05 | CpG33 | Yehuda et al., 2015^70^ |
|  |  |  |  |  |  | Not significant | *p*>.05 | CpG20 | Mcnerney et al., 2018^69^ |
|  |  |  |  |  |  | Not significant | *p*>.05 | CpG38 | Schur et al., 2017^66^ |
|  | chr5:142783678^6^ | 5'UTR, exon 1F |  |  |  | Not significant | *p*>.05 | CpG32 | Yehuda et al., 2015^70^ |
|  |  |  |  |  |  | Not significant | *p*>.05 | CpG19 | Mcnerney et al., 2018^69^ |
|  |  |  |  |  |  | Not significant | *p*>.05 | CpG37 | Schur et al., 2017^66^ |
|  | chr5:142783685^6^ | 5'UTR, exon 1F |  |  |  | Not significant | *p*>.05 | CpG31 | Yehuda et al., 2015^70^ |
|  |  |  |  |  |  | Not significant | *p*>.05 | CpG18 | Mcnerney et al., 2018^69^ |
|  |  |  |  |  |  | Not significant | *p*>.05 | CpG36 | Schur et al., 2017^66^ |
|  | chr5:142783688^6^ | 5'UTR, exon 1F |  |  |  | Not significant | *p*>.05 | CpG30 | Yehuda et al., 2015^70^ |
|  |  |  |  |  |  | Not significant | *p*>.05 | CpG17 | Mcnerney et al., 2018^69^ |
|  |  |  |  |  |  | Not significant | *p*>.05 | CpG35 | Schur et al., 2017^66^ |
|  | chr5:142783702^6^ | 5'UTR, exon 1F |  |  |  | Not significant | *p*>.05 | CpG29 | Yehuda et al., 2015^70^ |
|  |  |  |  |  |  | Not significant | *p*>.05 | CpG16 | Mcnerney et al., 2018^69^ |
|  |  |  |  |  |  | Not significant | *p*>.05 | CpG34 | Schur et al., 2017^66^ |
|  | chr5:142783712^6^ | 5'UTR, exon 1F |  |  |  | Not significant | *p*>.05 | CpG28 | Yehuda et al., 2015^70^ |
|  |  |  |  |  |  | Not significant | *p*>.05 | CpG15 | Mcnerney et al., 2018^69^ |
|  |  |  |  |  |  | Not significant | *p*>.05 | CpG33 | Schur et al., 2017^66^ |
|  | chr5:142783716^6^ | 5'UTR, exon 1F |  |  |  | Not significant | *p*>.05 | CpG27 | Yehuda et al., 2015^70^ |
|  |  |  |  |  |  | Not significant | *p*>.05 | CpG14 | Mcnerney et al., 2018^69^ |
|  |  |  |  |  |  | Not significant | *p*>.05 | CpG32 | Schur et al., 2017^66^ |
|  | chr5:142783730^6^ | 5'UTR, exon 1F |  |  |  | Not significant | *p*>.05 | CpG26 | Yehuda et al., 2015^70^ |
|  |  |  |  |  |  | Not significant | *p*>.05 | CpG13 | Mcnerney et al., 2018^69^ |
|  |  |  |  |  |  | Not significant | *p*>.05 | CpG31 | Schur et al., 2017^66^ |
|  | chr5:142783735^6^ | 5'UTR, exon 1F |  |  |  | Not significant | *p*>.05 | CpG25 | Yehuda et al., 2015^70^ |
|  |  |  |  |  |  | Not significant | *p*>.05 | CpG12 | Mcnerney et al., 2018^69^ |
|  |  |  |  |  |  | Not significant | *p*>.05 | CpG30 | Schur et al., 2017^66^ |
|  | chr5:142783742^6^ | 5'UTR, exon 1F |  |  |  | Not significant | *p*>.05 | CpG24 | Yehuda et al., 2015^70^ |
|  |  |  |  |  |  | Not significant | *p*>.05 | CpG11 | Mcnerney et al., 2018^69^ |
|  |  |  |  |  |  | Not significant | *p*>.05 | CpG29 | Schur et al., 2017^66^ |
|  | chr5:142783744^6^ | 5'UTR, exon 1F |  |  |  | ↓ Methylation in group with PTSD | *p*=.021* | CpG23 | Yehuda et al., 2015^70^ |
|  |  |  |  |  |  | Not significant | *p*>.05 | CpG10 | Mcnerney et al., 2018^69^ |
|  |  |  |  |  |  | Not significant | *p*>.05 | CpG28 | Schur et al., 2017^66^ |
|  | chr5:142783755^6^ | 5’UTR, exon 1F |  |  |  | Not significant | *p*>.05 | CpG22 | Yehuda et al., 2015^70^ |
|  |  |  |  |  |  | Not significant | *p*>.05 | CpG9 | Mcnerney et al., 2018^69^ |
|  |  |  |  |  |  | Not significant | *p*>.05 | CpG27 | Schur et al., 2017^66^ |
|  | chr5:142783766^6^ | 5’UTR, exon 1F |  |  |  | Not significant | *p*>.05 | CpG21 | Yehuda et al., 2015^70^ |
|  |  |  |  |  |  | Not significant | *p*>.05 | CpG8 | Mcnerney et al., 2018^69^ |
|  |  |  |  |  |  | Not significant | *p*>.05 | CpG26 | Schur et al., 2017^66^ |
|  | chr5:142783768^6^ | 5’UTR, exon 1F |  |  |  | Not significant | *p*>.05 | CpG20 | Yehuda et al., 2015^70^ |
|  |  |  |  |  |  | Not significant | *p*>.05 | CpG7 | Mcnerney et al., 2018^69^ |
|  |  |  |  |  |  | Not significant | *p*>.05 | CpG25 | Schur et al., 2017^66^ |
|  | chr5:142783771^6^ | 5’UTR, exon 1F |  |  |  | Not significant | *p*>.05 | CpG19 | Yehuda et al., 2015^70^ |
|  |  |  |  |  |  | Not significant | *p*>.05 | CpG6 | Mcnerney et al., 2018^69^ |
|  |  |  |  |  |  | Not significant | *p*>.05 | CpG24 | Schur et al., 2017^66^ |
|  | chr5:142783774^6^ | 5’UTR, exon 1F |  |  |  | Not significant | *p*>.05 | CpG18 | Yehuda et al., 2015^70^ |
|  |  |  |  |  |  | Not significant | *p*>.05 | CpG5 | Mcnerney et al., 2018^69^ |
|  |  |  |  |  |  | Not significant | *p*>.05 | CpG23 | Schur et al., 2017^66^ |
|  | chr5:142783777^6^ | 5’UTR, exon 1F |  |  |  | Not significant | *p*>.05 | CpG17 | Yehuda et al., 2015^70^ |
|  |  |  |  |  |  | Not significant | *p*>.05 | CpG4 | Mcnerney et al., 2018^69^ |
|  |  |  |  |  |  | Not significant | *p*>.05 | CpG22 | Schur et al., 2017^66^ |
|  | chr5:142783780^6^ | 5’UTR, exon 1F |  |  |  | Not significant | *p*>.05 | CpG16 | Yehuda et al., 2015^70^ |
|  |  |  |  |  |  | Not significant | *p*>.05 | CpG3 | Mcnerney et al., 2018^69^ |
|  |  |  |  |  |  | Not significant | *p*>.05 | CpG21 | Schur et al., 2017^66^ |
|  | chr5:142783785^6^ | 5’UTR, exon 1F |  |  |  | Not significant | *p*>.05 | CpG15 | Yehuda et al., 2015^70^ |
|  |  |  |  |  |  | Not significant | *p*>.05 | CpG2 | Mcnerney et al., 2018^69^ |
|  |  |  |  |  |  | Not significant | *p*>.05 | CpG20 | Schur et al., 2017^66^ |
|  | chr5:142783792^6^ | 5’UTR, exon 1F |  |  |  | Not significant | *p*>.05 | CpG14 | Yehuda et al., 2015^70^ |
|  |  |  |  |  |  | Not significant | *p*>.05 | CpG1 | Mcnerney et al., 2018^69^ |
|  |  |  |  |  |  | Not significant | *p*>.05 | CpG19 | Schur et al., 2017^66^ |
|  | chr5:142783809^6^ | 5’UTR, exon 1F |  |  |  | Not significant | *p*>.05 | CpG13 | Yehuda et al., 2015^70^ |
|  |  |  |  |  |  | Not significant | *p*>.05 | CpG18 | Schur et al., 2017^66^ |
|  | chr5:142783821^6^ | 5’UTR, exon 1F |  |  |  | Not significant | *p*>.05 | CpG12 | Yehuda et al., 2015^70^ |
|  |  |  |  |  |  | Not significant | *p*>.05 | CpG17 | Schur et al., 2017^66^ |
|  | chr5:142783831^6^ | 5’UTR, exon 1F |  |  |  | Not significant | *p*>.05 | CpG11 | Yehuda et al., 2015^70^ |
|  |  |  |  |  |  | Not significant | *p*>.05 | CpG16 | Schur et al., 2017^66^ |
|  | chr5:142783837^6^ | 5’UTR, exon 1F |  |  |  | Not significant | *p*>.05 | CpG10 | Yehuda et al., 2015^70^ |
|  |  |  |  |  |  | Not significant | *p*>.05 | CpG15 | Schur et al., 2017^66^ |
|  | chr5:142783843^6^ | 5’UTR, exon 1F |  |  |  | Not significant | *p*>.05 | CpG9 | Yehuda et al., 2015^70^ |
|  |  |  |  |  |  | Not significant | *p*>.05 | CpG14 | Schur et al., 2017^66^ |
|  | chr5:142783848^6^ | 5’UTR, exon 1F |  |  |  | Not significant | *p*>.05 | CpG8 | Yehuda et al., 2015^70^ |
|  |  |  |  |  |  | Not significant | *p*>.05 | CpG13 | Schur et al., 2017^66^ |
|  | chr5:142783853^6^ | 5’UTR, exon 1F |  |  |  | Not significant | *p*>.05 | CpG7 | Yehuda et al., 2015^70^ |
|  |  |  |  |  |  | Not significant | *p*>.05 | CpG12 | Schur et al., 2017^66^ |
|  | chr5:142783857^6^ | 5’UTR, exon 1F |  |  |  | Not significant | *p*>.05 | CpG6 | Yehuda et al., 2015^70^ |
|  |  |  |  |  |  | Not significant | *p*>.05 | CpG11 | Schur et al., 2017^66^ |
|  | chr5:142783859^6^ | 5’UTR, exon 1F |  |  |  | Not significant | *p*>.05 | CpG5 | Yehuda et al., 2015^70^ |
|  |  |  |  |  |  | Not significant | *p*>.05 | CpG10 | Schur et al., 2017^66^ |
|  | chr5:142783863^6^ | 5’UTR, exon 1F |  |  |  | Not significant | *p*>.05 | CpG4 | Yehuda et al., 2015^70^ |
|  |  |  |  |  |  | Not significant | *p*>.05 | CpG9 | Schur et al., 2017^66^ |
|  | chr5:142783869^6^ | 5’UTR, exon 1F |  |  |  | Not significant | *p*>.05 | CpG3 | Yehuda et al., 2015^70^ |
|  |  |  |  |  |  | Not significant | *p*>.05 | CpG8 | Schur et al., 2017^66^ |
|  | chr5:142783873^6^ | 5’UTR, exon 1F |  |  |  | Not significant | *p*>.05 | CpG2 | Yehuda et al., 2015^70^ |
|  |  |  |  |  |  | Not significant | *p*>.05 | CpG7 | Schur et al., 2017^66^ |
|  | chr5:142783883^6^ | 5’UTR, exon 1F |  |  |  | Not significant | *p*>.05 | CpG1 | Yehuda et al., 2015^70^ |
|  |  |  |  |  |  | Not significant | *p*>.05 | CpG6 | Schur et al., 2017^66^ |
|  | chr5:142783912^6^ | 5’UTR, exon 1F |  |  |  | Not significant | *p*>.05 | CpG5 | Schur et al., 2017^66^ |
|  | chr5:142783920^6^ | 5’UTR, exon 1F |  |  |  | Not significant | *p*>.05 | CpG4 | Schur et al., 2017^66^ |
|  | chr5:142783927^6^ | 5’UTR, exon 1F |  |  |  | Not significant | *p*>.05 | CpG3 | Schur et al., 2017^66^ |
|  | chr5:142783930^6^ | 5’UTR, exon 1F |  |  |  | Not significant | *p*>.05 | CpG2 | Schur et al., 2017^66^ |
|  | chr5:142783936^6^ | 5’UTR, exon 1F |  |  |  | Not significant | *p*>.05 | CpG1 | Schur et al., 2017^66^ |
|  | chr5:142784071^6^ | 5’UTR, exon 1B |  |  |  | Not significant | *p*>.05 | 1B-CpG29 | Labonte et al., 2014^64^ |
|  | chr5:142784074^6^ | 5’UTR, exon 1B |  |  |  | Not significant | *p*>.05 | 1B-CpG28 | Labonte et al., 2014^64^ |
|  | chr5:142784078^6^ | 5’UTR, exon 1B |  |  |  | Not significant | *p*>.05 | 1B-CpG27 | Labonte et al., 2014^64^ |
|  | chr5:142784082^6^ | 5’UTR, exon 1B |  |  |  | Not significant | *p*>.05 | 1B-CpG26 | Labonte et al., 2014^64^ |
|  | chr5:142784102^6^ | 5’UTR, exon 1B |  |  |  | Not significant | *p*>.05 | 1B-CpG25 | Labonte et al., 2014^64^ |
|  | chr5:142784110^6^ | 5’UTR, exon 1B |  |  |  | Not significant | *p*>.05 | 1B-CpG24 | Labonte et al., 2014^64^ |
|  |  |  |  |  |  |  |  |  |  |
|  | chr5:142784115^6^ | 5’UTR, exon 1B |  |  |  | Not significant | *p*>.05 | 1B-CpG23 | Labonte et al., 2014^64^ |
|  | chr5:142784118^6^ | 5’UTR, exon 1B |  |  |  | Not significant | *p*>.05 | 1B-CpG22 | Labonte et al., 2014^64^ |
|  | chr5:142784121^6^ | 5’UTR, exon 1B |  |  |  | Not significant | *p*>.05 | 1B-CpG21 | Labonte et al., 2014^64^ |
|  | chr5:142784124^6^ | 5’UTR, exon 1B |  |  |  | Not significant | *p*>.05 | 1B-CpG20 | Labonte et al., 2014^64^ |
|  | chr5:142784136^6^ | 5’UTR, exon 1B |  |  |  | Not significant | *p*>.05 | 1B-CpG19 | Labonte et al., 2014^64^ |
|  | chr5:142784139^6^ | 5’UTR, exon 1B |  |  |  | Not significant | *p*>.05 | 1B-CpG18 | Labonte et al., 2014^64^ |
|  | chr5:142784168 | 5’UTR, exon 1B |  |  |  | Not significant | *p*>.05 | 1B-CpG17 | Labonte et al., 2014^64^ |
|  | chr5:142784187 | 5’UTR, exon 1B |  |  |  | Not significant | *p*>.05 | 1B-CpG16 | Labonte et al., 2014^64^ |
|  | chr5:142784201^6^ | 5’UTR, exon 1B |  |  |  | Not significant | *p*>.05 | 1B-CpG15 | Labonte et al., 2014^64^ |
|  | chr5:142784222 | 5’UTR, exon 1B |  |  |  | ↓ Methylation in group with PTSD | *p*=.05* | 1B-CpG14 | Labonte et al., 2014^64^ |
|  | chr5:142784242^6^ | 5'UTR, exon 1B |  |  |  | Not significant | *p*>.05 | 1B-CpG13 | Labonte et al., 2014^64^ |
|  | chr5:142784278 | 5'UTR, exon 1B |  |  |  | Not significant | *p*>.05 | 1B-CpG12 | Labonte et al., 2014^64^ |
|  | chr5:142784304^6^ | 5'UTR, exon 1B |  |  |  | ↓ Methylation in group with PTSD | *p*<.05* | 1B-CpG11 | Labonte et al., 2014^64^ |
|  | chr5:142784323 | 5'UTR, exon 1B |  |  |  | Not significant | *p*>.05 | 1B-CpG10 | Labonte et al., 2014^64^ |
|  | chr5:142784369^6^ | 5'UTR, exon 1B |  |  |  | Not significant | *p*>.05 | 1B-CpG9 | Labonte et al., 2014^64^ |
|  | chr5:142784380^6^ | 5'UTR, exon 1B |  |  |  | Not significant | *p*>.05 | 1B-CpG8 | Labonte et al., 2014^64^ |
|  | chr5:142784382 | TSS1500; 5’UTR | ↑ Methylation in group with PTSD | *p*=.036 | cg18849621 | Not significant | *p*>.05 | 1B-CpG7 | Labonte et al., 2014^64^ |
|  | chr5:142784394^6^ | 5'UTR, exon 1B |  |  |  | Not significant | *p*>.05 | 1B-CpG6 | Labonte et al., 2014^64^ |
|  | chr5:142784412^6^ | 5'UTR, exon 1B |  |  |  | Not significant | *p*>.05 | 1B-CpG5 | Labonte et al., 2014^64^ |
|  | chr5:142784435^6^ | 5'UTR, exon 1B |  |  |  | ↓ Methylation in group with PTSD | *p*<.005* | 1B-CpG4 | Labonte et al., 2014^64^ |
|  | chr5:142784445^6^ | 5'UTR, exon 1B |  |  |  | ↓ Methylation in group with PTSD | *p*<.005* | 1B-CpG3 | Labonte et al., 2014^64^ |
|  | chr5:142784462^6^ | 5'UTR, exon 1B |  |  |  | ↓ Methylation in group with PTSD | *p*<.005* | 1B-CpG2 | Labonte et al., 2014^64^ |
|  | chr5:142784522^6^ | 5'UTR, exon 1B |  |  |  | Not significant | *p*>.05 | 1B-CpG1 | Labonte et al., 2014^64^ |
|  | chr5:142815463 | TSS1500 | ↓ Methylation in group with PTSD | *p*=.003 | cg07589972 |  |  |  |  |
|  |  |  |  |  |  |  |  |  |  |
| *OXTR* |  |  |  |  |  |  |  |  |  |
|  | chr3:8809464^6^ | Exon 3 |  |  |  | Not significant | *p*>.05 | CpG1 | Nawijn et al., 2019^71^ |
|  | chr3:8809442^6^ | Exon 3 |  |  |  | Not significant | *p*>.05 | CpG2 | Nawijn et al., 2019^71^ |
|  | chr3:8809437^6^ | Exon 3 |  |  |  | ↑ Methylation in females with PTSD | *p*=.022* | CpG3 | Nawijn et al., 2019^71^ |
|  | chr3:8809433^6^ | Exon 3 |  |  |  | Not significant | *p*>.05 | CpG4 | Nawijn et al., 2019^71^ |
|  | chr3:8809428^6^ | Exon 3 |  |  |  | Not significant | *p*>.05 | CpG5 | Nawijn et al., 2019^71^ |
|  | chr3:8809425^6^ | Exon 3 |  |  |  | Not significant | *p*>.05 | CpG6 | Nawijn et al., 2019^71^ |
|  | chr3:8809422^6^ | Exon 3 |  |  |  | Not significant | *p*>.05 | CpG7 | Nawijn et al., 2019^71^ |
|  | chr3:8809417^6^ | Exon 3 |  |  |  | Not significant | *p*>.05 | CpG8 | Nawijn et al., 2019^71^ |
|  | chr3:8809413^6^ | Exon 3 |  |  |  | ↑ Methylation in females with PTSD | *p*=.015* | CpG9 | Nawijn et al., 2019^71^ |
|  | chr3:8809399^6^ | Exon 3 |  |  |  | Not significant | *p*>.05 | CpG10 | Nawijn et al., 2019^71^ |
|  | chr3:8809394^6^ | Exon 3 |  |  |  | Not significant | *p*>.05 | CpG11 | Nawijn et al., 2019^71^ |
|  | chr3:8809387^6^ | Exon 3 |  |  |  | Not significant | *p*>.05 | CpG12 | Nawijn et al., 2019^71^ |
|  |  |  |  |  |  |  |  |  |  |
| *SKA2* | chr17:57187728 | 3'UTR |  |  |  | ↑ Methylation ↑ PTSD symptom severity | *p*=.021* | cg13989295 | Sadeh et al., 2016^72^ |
|  |  |  |  |  |  | ↓ Methylation ↑ PTSD symptom severity | *p*=.00006* | cg13989295 | Boks et al., 2016^73^ |
|  |  |  |  |  |  |  |  |  |  |
|  | chr17:57232722 | TSS1500; 5’UTR | ↑ Methylation in group with PTSD | *p*=.012 | cg20104110 |  |  |  |  |
|  |  |  |  |  |  |  |  |  |  |
| *SLC6A3* |  |  |  |  |  |  |  |  |  |
|  | chr5:1443460 | TSS200; TSS1500; 5’UTR | ↑ Methylation in group with P TSD | *p*=.017 | cg04088992 |  |  |  |  |
|  | chr5:1444395 | TSS1500; 5’UTR | ↑ Methylation in group with PTSD | *p*=.023 | cg13723431 |  |  |  |  |
|  | chr5:1444333 | TSS1500; 5’UTR | ↑ Methylation in group with PTSD | *p*=.033 | cg08713711 |  |  |  |  |
|  | chr5:1445542 | 1stExon; 5’UTR | ↑ Methylation in group with PTSD | *p*=.035 | cg04598517 |  |  |  |  |
|  | chr5:1446443 | TSS1500 |  |  |  | ↑ Methylation x *SLC6A3* VNTR 9R in group with PTSD | *p*=.008* | cg13202751 | Change et al., 2012^74^ |
|  |  |  |  |  |  |  |  |  |  |
| *SLC6A4* |  |  |  |  |  |  |  |  |  |
|  | chr17:28562220 | 5'UTR; 1stIntron |  |  |  | ↓ Methylation in group with PTSD x ↑ lifetime trauma exposure | *p*=.036* | cg22584138 | Koenen et al., 2011^75^ |
|  |  |  |  |  |  |  |  |  |  |

^1^ Identified using the GENECODE database; ^2^ identified using the Human Genome 19 (HG19) build from the Genome Reference Consortium;

^3^ identified using the University of California Santa Cruz (UCSC) Genomic Institute/Genome Browser; ^4^ Multiple listings indicate splice variants;

^5^CpG site not included on the MethylationEPIC beadchip.

**p* < .05 after correction for multiple testing

Abbreviations:

Pituitary adenylate cyclase-activating polypeptide 1 *(ADCYAP1)*; epigenome-wide association study (EWAS); transcription start site 200 (TSS200); transcription start site 1500 (TSS1500); 5’ untranslated region (5’UTR); not specified (NSF); posttraumatic stress disorder (PTSD); ADCYAP1 receptor 1 *(ADCYAP1R1)*; interferon-inducible protein / absent in melanoma 2 *(AIM2)*; brain-derived neurotropic factor *(BDNF)*; 3’ untranslated region (3’UTR); South shore (S_Shore); catechol-O-methyltransferase *(COMT)*; North shore (N_Shore); FK506 binding protein *(FKBP5)*; transcription start site (TSS); mindfulness based stress reduction (MBSR); glucocorticoid response element (GRE); 5-hydroxytryptamine receptor 3A *(HTR3A)*; interleukin 12B *(IL12B)*; mannosidase, alpha class 2c member 1 *(MAN2C1)*; monoamine oxidase A *(MAOA)*; nuclear receptor subfamily 3, group C *(NR3C1)*; transcription factor nerve growth factor-inducible protein A (NGFI-A); oxytocin receptor *(OXTR)*; spindle and kinetochore-associated protein 2 *(SKA2)*; 3’ untranslated region (3’UTR); solute carrier family 6, member 3 *(SLC6A3)*; variable number tandem repeat nine repeats (VNTR 9R); solute carrier family 6, member 4 *(SLC6A4)*.

Supplementary Table 13: *Summary of genes and CpG sites associations with PTSD observed in prior epigenome-wide studies alongside associations observed in the current epigenome-wide association study*

| **Gene name^1^** | **Genomic position^2^** | **Illumina ID** | **Location in gene^3,4^** | **Current EWAS** | | **Prior studies** | | |
| --- | --- | --- | --- | --- | --- | --- | --- | --- |
|  |  |  |  | **Finding** | ***p*-value** | **Finding** | ***p*-value** | **Reference** |
|  |  |  |  |  |  |  |  |  |
| *ACO11899.9* |  |  |  |  |  |  |  |  |
|  | Chr7:157294357 | cg26801037 | Intergenic |  |  | ↓ Methylation in group with PTSD | *p*=.000000308 | Smith et al., 2019^76^ |
|  |  |  |  |  |  |  |  |  |
| *ACP5* |  |  |  |  |  |  |  |  |
|  | Chr19:11688246 | cg07967308 | NSF |  |  | ↑ Methylation in group with PTSD | *p*=.0000080 | Smith et al., 2011^77^ |
|  | Chr19:11685647 | cg14188508 | 3’UTR | ↓ Methylation in group with PTSD | *p*=.046 |  |  |  |
|  | Chr19:11689855 | cg03302259 | TSS200/1500 | ↓ Methylation in group with PTSD | *p*=.049 |  |  |  |
|  |  |  |  |  |  |  |  |  |
| *AHRR* |  |  |  |  |  |  |  |  |
|  | Chr5:306699 | cg21813876 | TSS200; 3'UTR; 5'UTR | ↓ Methylation in group with PTSD | *p*=.001 |  |  |  |
|  | Chr5:314553 | cg18584368 | 5'UTR | ↑ Methylation in group with PTSD | *p*=.024 |  |  |  |
|  | Chr5:321320 | cg11554391 | TSS1500; 5'UTR | ↓ Methylation in group with PTSD | *p*=.044 |  |  |  |
|  | Chr5:369088 | cg12202185 | 5'UTR | ↓ Methylation in group with PTSD | *p*=.039 |  |  |  |
|  | Chr5:373378 | cg05575921 | 5'UTR | ↓ Methylation in group with PTSD | *p*=.033 | ↓ Methylation in group with PTSD | *p*=.0000000000472 | Smith et al., 2019^76^ |
|  |  |  |  |  |  |  |  |  |
|  |  |  |  |  |  |  |  |  |
|  | Chr5:374425 | cg22356527 | 5'UTR | ↑ Methylation in group with PTSD | *p*=.015 |  |  |  |
|  | Chr5:377358 | cg26703534 | 5'UTR | ↓ Methylation in group with PTSD | *p*=.031 | ↓ Methylation in group with PTSD | *p*=.0000000225 | Smith et al., 2019^76^ |
|  | Chr5:378854 | cg01097768 | 5'UTR | ↓ Methylation in group with PTSD | *p*=.048 |  |  |  |
|  | Chr5:388196 | cg01958142 | 5'UTR | ↓ Methylation in group with PTSD | *p*=.030 |  |  |  |
|  | Chr5:395444 | cg25648203 | Body |  |  | ↓ Methylation in group with PTSD | *p*=.0000000107 | Smith et al., 2019^76^ |
|  | Chr5:399360 | cg21161138 | Body |  |  | ↓ Methylation in group with PTSD | *p*=.000000000439 | Smith et al., 2019^76^ |
|  | Chr5:405488 | cg12207033 | 5'UTR | ↑ Methylation in group with PTSD | *p*=.044 |  |  |  |
|  | Chr5:405567 | cg01141993 | 5'UTR | ↑ Methylation in group with PTSD | *p*=.007 |  |  |  |
|  |  |  |  |  |  |  |  |  |
| *ANXA2* |  |  |  |  |  |  |  |  |
|  | Chr15:60690219 | cg08081036 | NSF |  |  | ↓ Methylation in group with PTSD | *p*=.0000093 | Smith et al., 2011^77^ |
|  |  |  |  |  |  |  |  |  |
| *ATP9A* |  |  |  |  |  |  |  |  |
|  | Chr20:50224119 | cg08403379 | Body | ↓ Methylation in group with PTSD | *p*=.035 |  |  |  |
|  | Chr20:50312490 | cg07339236 | Body |  |  | ↓ Methylation in group with PTSD | *p*=.000000239 | Smith et al., 2019^76^ |
|  |  |  |  |  |  |  |  |  |
| *BRSK1* |  |  |  |  |  |  |  |  |
|  | Chr19:55798650 | cg24351452 | Body | ↓ Methylation in group with PTSD | *p*=.004 |  |  |  |
|  | Chr19:55798477 | cg22577385 | Body | ↓ Methylation in group with PTSD | *p*=.030 |  |  |  |
|  | Chr19:55813339 | cg02357741 | Body |  |  | ↓ Methylation in group with PTSD | *p*=.00000224 | Mehta et al., 2017^78^ |
|  |  |  |  |  |  |  |  |  |
| *CDH15* |  |  |  |  |  |  |  |  |
|  | Chr16:89251975 | cg05901543 | Body |  |  | ↓ Methylation in group with PTSD | *p*=.0000025 | Snijders et al., 2020^79^ |
|  |  |  |  |  |  |  |  |  |
| *CLEC9A* |  |  |  |  |  |  |  |  |
|  | Chr12:10183167 | cg13830870 | TSS200; 3’UTR | ↓ Methylation in group with PTSD | *p*=.038 |  |  |  |
|  | Chr12:10183220 | cg26262442 | TSS200 | ↓ Methylation in group with PTSD | *p*=.010 |  |  |  |
|  | Chr12:10183234 | cg02930518 | TSS200 | ↓ Methylation in group with PTSD | *p*=.046 |  |  |  |
|  | Chr12:10183364 | cg20098659 | NSF |  |  | ↑ Methylation in group with PTSD | *p*=.0000043 | Smith et al., 2011^77^ |
|  | Chr12:10184399 | cg03900817 | TSS1500; 5’UTR | ↓ Methylation in group with PTSD | *p*=.035 |  |  |  |
|  |  |  |  |  |  |  |  |  |
| *COL1A2* |  |  |  |  |  |  |  |  |
|  | Chr7:93740160 | cg22676075 | NSF |  |  | ↓ Methylation ↑ PTSD symptom severity | *p*=.0000000129 | Rutten et al., 2018^80^ |
|  | Chr7:94023308 | cg24406898 | TSS1500 |  |  | ↓ Methylation ↑ PTSD symptom severity | *p*=.00000179 | Rutten et al., 2018^80^ |
|  | Chr7:94059900 | cg20943251 | 3'UTR; 5'UTR | ↑ Methylation in group with PTSD | *p*=.013 |  |  |  |
|  |  |  |  |  |  |  |  |  |
| *CTRC* |  |  |  |  |  |  |  |  |
|  | Chr1:15764093 | cg18917957 | TSS1500 |  |  | ↓ Methylation in group with PTSD | *p*=.00000050 | Snijders et al., 2020^79^ |
|  | Chr1:15773099 | cg13551783 | 3’UTR; 5’UTR | ↑ Methylation in group with PTSD | *p*=.023 |  |  |  |
|  |  |  |  |  |  |  |  |  |
| *DOCK2* |  |  |  |  |  |  |  |  |
|  | Chr5:169064897 | cg23184477 | 5'UTR | ↑ Methylation in group with PTSD | *p*=.032 |  |  |  |
|  | Chr5:169068404 | cg16277944 | 5'UTR |  |  | ↓ Methylation in group with PTSD | *p*=.00000495 | Mehta et al., 2017^78^ |
|  | Chr5:169083252 | cg14784010 | 5'UTR | ↑ Methylation in group with PTSD | *p*=.015 |  |  |  |
|  | Chr5:169146570 | cg26982433 | 5'UTR | ↓ Methylation in group with PTSD | *p*=.002 |  |  |  |
|  | Chr5:169416192 | cg10849016 | 5'UTR | ↑ Methylation in group with PTSD | *p*=.021 |  |  |  |
|  |  |  |  |  |  |  |  |  |
| *DUSP22* |  |  |  |  |  |  |  |  |
|  |  |  |  |  |  |  |  |  |
|  | Chr6:291882 | cg21548813 | TSS1500 |  |  | ↓ Methylation ↑ PTSD symptom severity | *p*=.0000000140 | Rutten et al., 2018^80^ |
|  |  |  |  | ↓ Methylation in group with PTSD | *p*=.032 |  |  |  |
|  | Chr6:291903 | cg03395511 | TSS200 |  |  | ↓ Methylation ↑ PTSD symptom severity | *p*=.000000234 | Rutten et al., 2018^80^ |
|  | Chr6:292329 | cg18110333 | 5'UTR |  |  | ↓ Methylation ↑ PTSD symptom severity | *p*=.00000000689 | Rutten et al., 2018^80^ |
|  | Chr6:292522 | cg11235426 | 5'UTR |  |  | ↓ Methylation ↑ PTSD symptom severity | *p*=.000000665 | Rutten et al., 2018^80^ |
|  | Chr6:292596 | cg01516881 | Body |  |  | ↓ Methylation ↑ PTSD symptom severity | *p*=.000000452 | Rutten et al., 2018^80^ |
|  |  |  |  |  |  |  |  |  |
| *FLJ46321* |  |  |  |  |  |  |  |  |
|  | Chr9:84609982 | cg14405344 | Body |  |  | ↓ Methylation in group with PTSD | *p*=.000000786 | Smith et al., 2019^76^ |
|  |  |  |  |  |  |  |  |  |
| *G0S2* |  |  |  |  |  |  |  |  |
|  | Chr1:209849006 | cg19534438 | 5'UTR |  |  | ↑ Methylation in group with PTSD | *p*=.000000119 | Logue et al., 2020^81^ |
|  |  |  |  |  |  |  |  |  |
| *HDAC4* |  |  |  |  |  |  |  |  |
|  | Chr2:240062051 | cg25828346 | 3’UTR; 5’UTR | ↓ Methylation in group with PTSD | *p*=.033 |  |  |  |
|  | Chr2:240066248 | cg06107260 | 3’UTR; 5’UTR | ↑ Methylation in group with PTSD | *p*=.046 |  |  |  |
|  | Chr2:240168862 | cg22937172 | 5’UTR |  |  | ↑ Methylation in group with PTSD | *p*=.000000127 | Maddox et al., 2018^82^ |
|  | Chr2:240196171 | cg16348668 | 3’UTR; 5’UTR | ↑ Methylation in group with PTSD | *p*=.017 |  |  |  |
|  | Chr2:240205260 | cg00116699 | 3’UTR; 5’UTR | ↓ Methylation in group with PTSD | *p*=.013 |  |  |  |
|  | Chr2:240205602 | cg20453985 | 3’UTR; 5’UTR | ↓ Methylation in group with PTSD | *p*=.039 |  |  |  |
|  | Chr2:240218482 | cg09664216 | 3’UTR; 5’UTR | ↑ Methylation in group with PTSD | *p*=.022 |  |  |  |
|  | Chr2:240288826 | cg06381933 | 5’UTR | ↑ Methylation in group with PTSD | *p*=.004 |  |  |  |
|  | Chr2:240309587 | cg02812817 | 3’UTR; 5’UTR | ↑ Methylation in group with PTSD | *p*=.021 |  |  |  |
|  | Chr2:240313052 | cg00811555 | 3’UTR; 5’UTR | ↓ Methylation in group with PTSD | *p*=.008 |  |  |  |
|  |  |  |  |  |  |  |  |  |
| *HEXDC* |  |  |  |  |  |  |  |  |
|  | Chr17:80375674 | cg07050946 | TSS1500 | ↓ Methylation in group with PTSD | *p*=.020 |  |  |  |
|  | Chr17:80376872 | cg12655836 | TSS1500; 5’UTR | ↓ Methylation in group with PTSD | *p*=.012 |  |  |  |
|  | Chr17:80394529 | cg20756026 | Body |  |  | ↓ Methylation in group with PTSD | *p*=.000000013 | Snijders et al., 2020^79^ |
|  |  |  |  |  |  |  |  |  |
| *HGS* |  |  |  |  |  |  |  |  |
|  | Chr17:79658554 | cg19577098 | Body |  |  | ↓ Methylation in group with PTSD | *p*=.000000147 | Uddin et al., 2018^83^ |
|  |  |  |  |  |  |  |  |  |
| *HIST1H2APS2* |  |  |  |  |  |  |  |  |
|  | Chr6:25882590 | cg03517284 | NSF |  |  | ↓ Methylation ↑ PTSD symptom severity | *p*=.013 | Rutten et al., 2018^80^ |
|  |  |  |  |  |  |  |  |  |
| *HOOK2* |  |  |  |  |  |  |  |  |
|  | Chr19:12874033 | cg07798386 | 3'UTR | ↑ Methylation in group with PTSD | *p*=.002 |  |  |  |
|  | Chr19:12876846 | cg06417478 | Body |  |  | ↓ Methylation ↑ PTSD symptom severity | *p*=.000000000288 | Rutten et al., 2018^80^ |
|  | Chr19:12876947 | cg04657146 | Body |  |  | ↓ Methylation ↑ PTSD symptom severity | *p*=.0000000345 | Rutten et al., 2018^80^ |
|  | Chr19:12877000 | cg11738485 | Body |  |  | ↓ Methylation ↑ PTSD symptom severity | *p*=.0000000129 | Rutten et al., 2018^80^ |
|  |  |  |  |  |  |  |  |  |
| *LCN8* |  |  |  |  |  |  |  |  |
|  | Chr9:139652023 | cg16004906 | TSS200/1500 | ↓ Methylation in group with PTSD | *p*=.016 |  |  |  |
|  | Chr9:139653461 | cg13839917 | TSS1500 | ↓ Methylation in group with PTSD | *p*=.031 |  |  |  |
|  | Chr9:139653533 | cg09325682 | TSS1500 |  |  | ↓ Methylation in group with PTSD | *p*=.00000328 | Mehta et al., 2017^78^ |
|  |  |  |  |  |  |  |  |  |
| *LINC00599* |  |  |  |  |  |  |  |  |
|  | Chr8:9742024 | cg18217048 | Intergenic |  |  | ↓ Methylation in group with PTSD | *p*=.000000961 | Smith et al., 2019^76^ |
|  |  |  |  |  |  |  |  |  |
| *LRRC3B* |  |  |  |  |  |  |  |  |
|  | Chr3:26663805 | cg10332616 | TSS1500; 3’UTR | ↓ Methylation in group with PTSD | *p*=.021 |  |  |  |
|  | Chr3:26664115 | cg13787438 | TSS200/1500; 3'UTR | ↓ Methylation in group with PTSD | *p*=.029 |  |  |  |
|  | Chr3:26688128 | cg12975201 | 5'UTR | ↑ Methylation in group with PTSD | *p*=.013 |  |  |  |
|  | Chr3:26753752 | cg26499155 | NSF |  |  | ↑ Methylation in group with PTSD | *p*=.000000794 | Mehta et al., 2017^78^ |
|  |  |  |  |  |  |  |  |  |
| *MAD1L1* |  |  |  |  |  |  |  |  |
|  | Chr7:1923695 | cg12169700 | Body |  |  | ↓ Methylation in group with PTSD | *p*=.00000000081 | Snijders et al., 2020^79^ |
|  | Chr7:2020501 | cg17633015 | TSS200 | ↓ Methylation in group with PTSD | *p*=.015 |  |  |  |
|  | Chr7:2250274 | cg01859191 | 3’UTR | ↓ Methylation in group with PTSD | *p*=.036 |  |  |  |
|  | Chr7:2256023 | cg00420390 | TSS1500; 3’UTR | ↓ Methylation in group with PTSD | *p*=.028 |  |  |  |
|  | Chr7:2257611 | cg17718984 | 3’UTR | ↓ Methylation in group with PTSD | *p*=.044 |  |  |  |
|  | Chr7:2274072 | cg11571856 | TSS1500; 3’UTR | ↑ Methylation in group with PTSD | *p*=.015 |  |  |  |
|  |  |  |  |  |  |  |  |  |
| *MIR3179* |  |  |  |  |  |  |  |  |
|  | Chr13:98749760 | cg17284326 | Intergenic |  |  | ↓ Methylation in group with PTSD | *p*=.0000000893 | Smith et al., 2019^76^ |
|  |  |  |  |  |  |  |  |  |
| *MYT1L* |  |  |  |  |  |  |  |  |
|  | Chr2:1817351 | cg10075506 | Body | ↑ Methylation in group with PTSD | *p*=.036 | ↓ Methylation ↑ PTSD symptom severity | *p*=.00000128 | Rutten et al., 2018^80^ |
|  |  |  |  |  |  |  |  |  |
| *NGF* |  |  |  |  |  |  |  |  |
|  | Chr1:115829856 | cg12647496 | 5'UTR | ↓ Methylation in group with PTSD | *p*=.020 |  |  |  |
|  | Chr1:115844232 | cg17750109 | 5'UTR |  |  | ↓ Methylation in group with PTSD | *p*=.00000306 | Mehta et al., 2017^78^ |
|  | Chr1:115877962 | cg01804281 | 5'UTR | ↓ Methylation in group with PTSD | *p*=.011 |  |  |  |
|  |  |  |  |  |  |  |  |  |
| *NINJ2* |  |  |  |  |  |  |  |  |
|  | Chr12:739980 | cg14911689 | Body |  |  | ↓ Methylation ↑ PTSD symptom severity | *p*=.00000000861 | Rutten et al., 2018^80^ |
|  | Chr12:740100 | cg26654770 | Body |  |  | ↓ Methylation ↑ PTSD symptom severity | *p*=.000000610 | Rutten et al., 2018^80^ |
|  | Chr12:751121 | cg11552370 | TSS200/1500; 3'UTR; 5'UTR | ↓ Methylation in group with PTSD | *p*=.004 |  |  |  |
|  | Chr12:751317 | cg21334546 | TSS1500; 3'UTR; 5'UTR | ↓ Methylation in group with PTSD | *p*=.012 |  |  |  |
|  | Chr12:772861 | cg00237010 | TSS200; 5'UTR | ↓ Methylation in group with PTSD | *p*=.020 |  |  |  |
|  |  |  |  |  |  |  |  |  |
| *NRG1* |  |  |  |  |  |  |  |  |
|  | Chr8:31996079 | cg23637605 | 5’UTR |  |  | ↓ Methylation in group with PTSD | *p*=.0000000753 | Uddin et al., 2018^83^ |
|  | Chr8: 32577951 | cg10157574 | TSS1500; 5’UTR | ↑ Methylation in group with PTSD | *p*=.009 |  |  |  |
|  | Chr8:32504400 | cg05861515 | TSS1500; 5’UTR | ↑ Methylation in group with PTSD | *p*=.014 |  |  |  |
|  | Chr12:772966 | cg15884713 | TSS200 | ↓ Methylation in group with PTSD | *p*=.038 |  |  |  |
|  |  |  |  |  |  |  |  |  |
| *PAX8* |  |  |  |  |  |  |  |  |
|  | Chr2:113991662 | cg19081868 | TSS1500; 3'UTR; 5'UTR | ↓ Methylation in group with PTSD | *p*=.026 |  |  |  |
|  | Chr2:113992694 | cg23122642 | TSS1500; 3'UTR; 5'UTR | ↑ Methylation in group with PTSD | *p*=.048 |  |  |  |
|  | Chr2:113992921 | cg11763394 | TSS200/1500; 3'UTR; 5'UTR | ↑ Methylation in group with PTSD | *p*=.030 | ↓ Methylation ↑ PTSD symptom severity | *p*=.00000129 | Rutten et al., 2018^80^ |
|  | Chr2:113992930 | cg21550016 | TSS200/1500; 3'UTR; 5'UTR | ↑ Methylation in group with PTSD | *p*=.047 |  |  |  |
|  | Chr2:113994578 | cg00422909 | TSS200 /1500; 3'UTR; 5'UTR | ↑ Methylation in group with PTSD | *p*=.0009 |  |  |  |
|  | Chr2:113998034 | cg17774569 | TSS1500; 5'UTR | ↓ Methylation in group with PTSD | *p*=.028 |  |  |  |
|  | Chr2:114036444 | cg05194362 | TSS1500; 3'UTR; 5'UTR | ↑ Methylation in group with PTSD | *p*=.036 |  |  |  |
|  |  |  |  |  |  |  |  |  |
| *RNF6* |  |  |  |  |  |  |  |  |
|  | Chr13:26795862 | cg25415650 | 5'UTR |  |  | ↑ Methylation in group with PTSD | *p*=.0000000738 | Smith et al., 2019^76^ |
|  | Chr13:26796956 | cg27472353 | TSS200/1500 | ↓ Methylation in group with PTSD | *p*=.015 |  |  |  |
|  | Chr13:26797157 | cg03033508 | TSS1500 | ↓ Methylation in group with PTSD | *p*=.013 |  |  |  |
|  | Chr13:26797279 | cg23140290 | TSS1500 | ↓ Methylation in group with PTSD | *p*=.032 |  |  |  |
|  |  |  |  |  |  |  |  |  |
| *SDK1* |  |  |  |  |  |  |  |  |
|  | Chr7:3343581 | cg11998431 | TSS1500; 5'UTR | ↓ Methylation ↑ PTSD symptom severity | *p*=.006 |  |  |  |
|  | Chr7:3343830 | cg19871079 | 5'UTR | ↑ Methylation in group with PTSD | *p*=.034 |  |  |  |
|  | Chr7:3470434 | cg02716317 | 5'UTR | ↑ Methylation in group with PTSD | *p*=.043 |  |  |  |
|  | Chr7:3478077 | cg25451765 | 5'UTR | ↑ Methylation in group with PTSD | *p*=.008 |  |  |  |
|  | Chr7:3920006 | cg18620306 | 5'UTR | ↑ Methylation in group with PTSD | *p*=.002 |  |  |  |
|  | Chr7:3920082 | cg05812582 | 5'UTR | ↑ Methylation in group with PTSD | *p*=.014 |  |  |  |
|  | Chr7:3920297 | cg05896377 | 5'UTR | ↑ Methylation in group with PTSD | *p*=.012 |  |  |  |
|  | Chr7:3920534 | cg18022346 | 5'UTR | ↑ Methylation in group with PTSD | *p*=.042 |  |  |  |
|  | Chr7:3989167 | cg01923516 | TSS1500; 5'UTR | ↑ Methylation in group with PTSD | *p*=.045 |  |  |  |
|  | Chr7:4018931 | cg14950237 | 5'UTR | ↑ Methylation in group with PTSD | *p*=.016 |  |  |  |
|  | Chr7:4049819 | cg24851600 | 5'UTR | ↓ Methylation ↑ PTSD symptom severity | *p*=.037 |  |  |  |
|  | Chr7:4052042 | cg09274366 | 5'UTR | ↓ Methylation ↑ PTSD symptom severity | *p*=.011 |  |  |  |
|  | Chr7:4056929 | cg11082847 | 5'UTR | ↓ Methylation ↑ PTSD symptom severity | *p*=.028 |  |  |  |
|  | Chr7:4146971 | cg27215475 | 5'UTR | ↑ Methylation in group with PTSD | *p*=.047 |  |  |  |
|  | Chr7:4152976 | cg14682731 | 5'UTR | ↑ Methylation in group with PTSD | *p*=.009 |  |  |  |
|  | Chr7:4167822 | cg12801000 | TSS1500; 5'UTR | ↑ Methylation in group with PTSD | *p*=.030 |  |  |  |
|  | Chr7:4175403 | cg01667575 | 5'UTR | ↑ Methylation in group with PTSD | *p*=.047 |  |  |  |
|  | Chr7:4183976 | cg04612959 | 5'UTR | ↑ Methylation in group with PTSD | *p*=.002 |  |  |  |
|  | Chr7:4191467 | cg21108029 | 5'UTR | ↑ Methylation in group with PTSD | *p*=.028 |  |  |  |
|  | Chr7:4213731 | cg06610094 | TSS200; 5'UTR | ↓ Methylation ↑ PTSD symptom severity | *p*=.008 |  |  |  |
|  | Chr7:4228775 | cg23357832 | 5'UTR | ↑ Methylation in group with PTSD | *p*=.039 |  |  |  |
|  | Chr7:4228948 | cg26800883 | 5'UTR | ↑ Methylation in group with PTSD | *p*=.034 |  |  |  |
|  | Chr7:4244643 | cg07249765 | Body |  |  | ↓ Methylation ↑ PTSD symptom severity | *p*=.0000000541 | Rutten et al., 2018^80^ |
|  | Chr7:4276193 | cg03010092 | 5'UTR | ↓ Methylation ↑ PTSD symptom severity | *p*=.006 |  |  |  |
|  | Chr7:4304779 | cg16956686 | Body |  |  | ↓ Methylation in group with PTSD | *p*=.00000020 | Snijders et al., 2020^79^ |
|  | Chr7:4306321 | cg22611217 | 3'UTR; 5'UTR | ↑ Methylation in group with PTSD | *p*=.001 |  |  |  |
|  | Chr7:4308120 | cg21853331 | 3'UTR; 5'UTR | ↑ Methylation in group with PTSD | *p*=.009 |  |  |  |
|  |  |  |  |  |  |  |  |  |
| *SPRY4* |  |  |  |  |  |  |  |  |
|  | Chr5:141660565 | cg05656210 | NSF |  |  | ↓ Methylation in group with PTSD | *p*=.000000010 | Snijders et al., 2020^79^ |
|  |  |  |  |  |  |  |  |  |
| *TLR8* |  |  |  |  |  |  |  |  |
|  | ChrX:12924783 | cg07759587 | NSF |  |  | ↑ Methylation in group with PTSD | *p*=.000011 | Smith et al., 2011^77^ |
|  |  |  |  |  |  |  |  |  |
| *TPR* |  |  |  |  |  |  |  |  |
|  | Chr1:186344558 | cg24577137 | TSS200/1500 | ↓ Methylation in group with PTSD | *p*=.0008 | ↓ Methylation in group with PTSD | *p*=.0000019 | Smith et al., 2011^77^ |
|  |  |  |  |  |  |  |  |  |

^1^ Identified using the GENECODE database; ^2^ identified using the Human Genome 19 (HG19) build from the Genome Reference Consortium;

^3^ identified using the University of California Santa Cruz (UCSC) Genomic Institute/Genome Browser; ^4^ Multiple listings indicate splice

variants.

Abbreviations:

Anaphase promoting complex subunit 5 (*APC5*); not specified (NSF); posttraumatic stress disorder (PTSD); untranslated region (3’UTR); North shelf (N_Shelf); transcription start site 200 (TSS200); transcription start site 1500 (TSS1500); South shore (S_Shore); human aryl hydrocarbon receptor repressor *(AHRR)*; 5’ untranslated region (5’UTR); North shore (N_Shore); South shelf (S_Shelf); annexin A2 (*ANXA2*); ATPase phospholipid transporting 9A *(ATP9A)*; brain-specific serine/threonine-protein kinase 1 *(BRSK1);* cadherin 15 *(CDH15)*; C-type lectin domain family 9, member A (*CLEC9A*); collagen type I alpha 2 chain *(COL1A2);* chymotryosin C *(CTRC);* dedicator of cytokinesis 2 *(DOCK2);* dual specificity phosphatase 22 *(DUSP22)*; family with sequence similarity 75, member D1 *(FLJ46321);* putative lymphocyte G0/G1 switch *(G0S2);* histone deacetylase 4 *(HDAC4);* hexosaminidase glycosyl hydrolase family 20 catalytic domain containing *(HEXDC);* hepatocyte growth factor-regulated tyrosine kinase substrate *(HGS)* H2A histone family, member T, pseudogene *(HIST1H2APS2);* hook microtubule tethering protein 2 *(HOOK2)*; lipocalin 8 *(LCN8)*; long intergenic non-protein coding RNA 599 *(LINC00599);* leucine rich repeat containing 3B *(LRRC3B);* mitotic arrest deficient 1 like 1 *(MAD1L1);* microRNA 3170 *(MIR3170)*; myelin transcription factor 1 like *(MYT1L);* nerve growth factor *(NGF)*; ninjurin 2 *(NINJ2)*; neuregulin 1 *(NRG1);* paired box 8 *(PAX8)*; ring finger protein 6 *(RNF6)*; sidekick cell adhesion molecule 1 *(SDK1)*; sprouty RTK signalling antagonist 4 *(SPRY4)*; toll-like receptor 8 (*TLR8*); translocated promoter region (*TPR*).

Supplementary Table 14: *Univariate relationship between baseline confounding/covarying factors, PTSD, BRSK2 and ADCYAP1 methylation*

|  | Age  baseline | | HIV status (negative vs positive) | | BMI  baseline | | Smoker  (no vs yes) | | Childhood trauma  baseline | | Lifetime trauma  baseline | | Alcohol use  baseline | | Depression baseline | | Medication use (no vs yes) | |
| --- | --- | --- | --- | --- | --- | --- | --- | --- | --- | --- | --- | --- | --- | --- | --- | --- | --- | --- |
|  | *r/z* | *p* | *z/x^2^* | *p* | *r/z* | *p* | *z/ x^2^* | *p* | *r/z* | *p* | *r/z* | *p* | *r/z* | *p* | *r/z* | *p* | *z/x^2^* | *p* |
|  |  |  |  |  |  |  |  |  |  |  |  |  |  |  |  |  |  |  |
| PTSD total score (baseline) | -0.20 | .052 | -1.07 | .284 | 0.08 | .455 | -.487 | .626 | 0.25 | .013* | 0.06 | .550 | -0.07 | .514 | 0.50 | .000* | -1.46 | .144 |
| PTSD total score (3-months) | -0.12 | .252 | -.139 | .889 | -0.04 | .720 | .000 | 1.00 | 0.16 | .118 | 0.06 | .552 | -0.05 | .650 | 0.06 | .568* | -1.08 | .282 |
| PTSD status (3-months) | 0.12 | .908 | 0.93 | .336 | 0.99 | .323 | 0.01 | .937 | 0.70 | .482 | 0.14 | .889 | 0.38 | .706 | 0.14 | .890 | 0.78 | .378 |
| PTSD total score (6-months) | 0.11 | .286 | -.631 | .528 | -0.02 | .884 | -.288 | .773 | 0.04 | .723 | 0.05 | .646 | -0.20 | .049* | 0.22 | .029* | -.513 | .608 |
| PTSD status (6-months) | 1.93 | .054 | 0.50 | .478 | 0.51 | .609 | 0.27 | .602 | 0.86 | .389 | 1.80 | .071 | 1.41 | .158 | 2.01 | .044* | 0.42 | .516 |
|  |  |  |  |  |  |  |  |  |  |  |  |  |  |  |  |  |  |  |
| *BRSK2* CpG3 (baseline) | -0.01 | .918 | -1.01 | .313 | -0.05 | .662 | -.316 | .752 | -0.19 | .070 | -0.19 | .061 | 0.10 | .332 | 0.11 | .287 | -.540 | .589 |
| *BRSK2* CpG4 (baseline) | -0.04 | .725 | -1.01 | .313 | -0.09 | .382 | -.543 | .587 | -0.26 | .011* | -0.31 | .003* | 0.09 | .405 | 0.11 | .269 | -.933 | .351 |
| *BRSK2* CpG5 (baseline) | -0.10 | .335 | -1.38 | .169 | -0.08 | .466 | -.587 | .557 | -0.22 | .032* | -0.27 | .008* | 0.08 | .457 | 0.16 | 123 | -1.57 | .117 |
|  |  |  |  |  |  |  |  |  |  |  |  |  |  |  |  |  |  |  |
| *BRSK2* CpG3 (3-months) | -0.01 | .905 | -.332 | .740 | 0.02 | .844 | -.062 | .951 | -0.16 | .114 | -0.14 | .169 | 0.08 | .466 | 0.13 | .208 | -.307 | .759 |
| *BRSK2* CpG4 (3-months) | -0.07 | .508 | -1.15 | .249 | 0.08 | .451 | -.471 | .638 | -0.15 | .149 | -0.21 | .040* | 0.11 | .292 | 0.18 | .086 | -1.05 | .295 |
| *BRSK2* CpG5 (3-months) | -0.06 | .581 | -.853 | .394 | 0.04 | .740 | -.419 | .675 | -.127 | .224 | -0.13 | .214 | 0.06 | .560 | 0.14 | .190 | -.853 | .394 |
|  |  |  |  |  |  |  |  |  |  |  |  |  |  |  |  |  |  |  |
| *BRSK2* CpG3 (6-months) | -0.02 | .866 | -.607 | .544 | -0.00 | .990 | -.291 | .771 | -0.15 | .141 | -0.26 | .012* | 0.09 | .385 | 0.20 | .058 | -.272 | .786 |
| *BRSK2* CpG4 (6-months) | 0.00 | .967 | -.898 | .369 | 0.02 | .860 | -.768 | .443 | -0.10 | .335 | -0.22 | .034* | 0.08 | .434 | 0.24 | .020* | -.232 | .816 |
| *BRSK2* CpG5 (6-months) | -0.02 | .840 | -.935 | .350 | -0.04 | .723 | -.224 | .823 | -0.19 | .063 | -0.24 | .020* | 0.08 | .445 | 0.19 | .073 | -.745 | .456 |
|  |  |  |  |  |  |  |  |  |  |  |  |  |  |  |  |  |  |  |
| *ADCYAP1* CpG1&2 (baseline) | -0.40 | .698 | -1.43 | .154 | 0.02 | .834 | -1.34 | .179 | 0.04 | .670 | -0.07 | .513 | 0.14 | .163 | 0.19 | .063 | -.548 | .583 |
|  |  |  |  |  |  |  |  |  |  |  |  |  |  |  |  |  |  |  |
| *ADCYAP1* CpG1&2 (3-months) | -0.15 | .314 | -2.13 | .034* | 0.11 | .488 | -1.22 | .221 | 0.28 | .066 | 0.09 | .559 | 0.00 | .967 | 0.12 | .269 | -.703 | .482 |
|  |  |  |  |  |  |  |  |  |  |  |  |  |  |  |  |  |  |  |
| *ADCYAP1* CpG1&2 (6-months) | -0.14 | .162 | -.060 | .952 | -0.11 | .306 | -1.22 | .223 | 0.02 | .815 | 0.04 | .698 | 0.15 | .136 | -0.10 | .314 | -.380 | .704 |
|  |  |  |  |  |  |  |  |  |  |  |  |  |  |  |  |  |  |  |

Abbreviations: posttraumatic stress disorder (PTSD), brain-specific serine/threonine-protein kinase 2 (*BRSK2*), adenylate cyclase activating polypeptide 1 (*ADCYAP1)*

**REFERENCES**

1. Abrahams N, Seedat S, Lombard C, et al. Study protocol for a longitudinal study evaluating the impact of rape on women’s health and their use of health services in South Africa. *BMJ Open*. 2017;7(9):1-13. doi:10.1136/bmjopen-2017-017296

2. Sheehan D, Yves L, Sheehan K, et al. The Mini-International Neuropsychiatric Interview (M.I.N.I): The development and validation of a structured diagnostic psychiatric interview for DSM-IV and ICD-10. *J Clin Psychiatry*. 1998;59(20):34-57.

3. Lecrubier Y, Sheehan D, Weiler E, et al. The Mini International Neuropsychiatric Interview (MINI). A short diagnostic structured interview: reliability and validity according to the CIDI. *Eur Psychiatry*. 1997;12(5):224-231.

4. Marques J, Zuardi A. Validity and applicability of the Mini International Neuropsychiatric Interview administered by family medicine residents in primary health care in Brazil. *Gen Hosp Psychiatry*. 2008;30(4):303-310.

5. Davidson JRT, Book SW, Colket JT, et al. Assessment of a new self-rating scale for post-traumatic stress disorder. *Psychol Med*. 1997;27(1):153-160. doi:10.1017/S0033291796004229

6. McDonald S, Beckham J, Morey R, Calhoun P. The validity and diagnostic efficiency of the Davidson Trauma Scale in military veterans who have served since September 11th 2001. *J Anxiety Disord*. 2009;23(2):247-255. doi:10.1038/jid.2014.371

7. Chirwa ED, Sikweyiya Y, Addo-Lartey AA, et al. Prevalence and risk factors of physical or sexual intimate violence perpetration amongst men in four districts in the central region of Ghana: Baseline findings from a cluster randomised controlled trial. *PLoS One*. 2018;13(3):1-18. doi:10.1371/journal.pone.0191663

8. Bernstein D, Fink L. *Childhood Trauma Questionnaire: A Retrospective Self-Report Manual*. San Antonio: The Psychological Corporation; 1998.

9. Villano C, Cleland C, Rosenblum A, et al. Psychometric utility of the childhood trauma questionnaire with female street-based sex workers. *J Trauma Dissociation*. 2004;5(3):33-41. doi:10.1300/J229v05n03

10. Scher CD, Stein MB, Asmundson GJG, Mccreary DR, Forde DR. The Childhood Trauma Questionnaire in a community sample: Psychometric properties and normative data. *J Trauma Stress*. 2001;14(4):843-857. doi:10.1023/A:1013058625719

11. Jewkes R, Nduna M, Levin J, et al. A cluster randomized-controlled trial to determine the effectiveness of Stepping Stones in preventing HIV infections and promoting safer sexual behaviour amongst youth in the rural Eastern Cape, South Africa: Trial design, methods and baseline findings. *Trop Med Int Heal*. 2006;11(1):3-16. doi:10.1111/j.1365-3156.2005.01530.x

12. Weathers F, Blake D, Schnurr P, Kaloupek D, Marx B, Keane T. *The Life Events Checklist for DSM-5 (LEC-5)*. USA: National Center for PTSD; 2013.

13. Saunders J, Aasland O, Babor T, De La Fuente J, Grant M. Development of the Alcohol Use Disorders Identification Test (AUDIT): WHO Collaborative Project on Early Detection of Persons with Harmful Alcohol Consumption-II. *Addiction*. 1993;88(6):791-804. doi:10.1111/j.1360-0443.1993.tb02093.x

14. Bush K, Kivlahan D, McDonnel M, Fihn S, Bradley K. The AUDIT Alcohol Consumption Questions (AUDIT-C). *Arch Intern Med*. 1998;158(8):1789-1795. doi:10.1097/00000374-199811000-00034

15. Bohn MJ, Babor TF, Kranzler HR. The Alcohol Use Disorders Identification Test (AUDIT): validation of a screening instrument for use in medical settings. *J Stud Alcohol*. 1995;56(4):423-432. doi:10.15288/jsa.1995.56.423

16. Cherpitel CJ, Clark WB. Ethnic Differences in Performance of Screening Instruments for Identifying Harmful Drinking and Alcohol Dependence in the Emergency Room. *Alcohol Clin Exp Res*. 1995;19(3):628-634. doi:10.1111/j.1530-0277.1995.tb01559.x

17. Medina-Mora E, Carreño S, De la Fuente JR. Experience with the Alcohol Use Disorders Identification Test (AUDIT) in Mexico. In: *Recent Developments in Alcoholism*. Boston, MA: Springer US; 1998:383-396. doi:10.1007/0-306-47148-5_19

18. Leung SF, Arthur D. The alcohol use disorders identification test (AUDIT): validation of an instrument for enhancing nursing practice in Hong Kong. *Int J Nurs Stud*. 2000;37(1):57-64. doi:10.1016/S0020-7489(99)00052-8

19. Chinyadza E, Moyo IM, Katsumbe TM, et al. Alcohol problems among patients attending five primary health care clinics in Harare city. *Cent Afr J Med*. 1993;39(2):26-32. http://www.ncbi.nlm.nih.gov/pubmed/8261500.

20. Seale JP. Prevalence of Problem Drinking in a Venezuelan Native American Population. *Alcohol Alcohol*. 2002;37(2):198-204. doi:10.1093/alcalc/37.2.198

21. Simbayi L, Kalichman S, Jooste S, Mathiti V, Cain D, Cherry C. Alcohol use and sexual risks for HIV infection among men and women receiving sexually transmitted infection clinic services in Cape Town, South Africa. *J Stud Alcohol*. 2004;65:434-442.

22. Radloff L. The CES-D Scale: A Self-Report Depression Scale for Research in the General Population. *Appl Psychol Meas*. 1977;1(3):385-401. doi:10.1002/ardp.19552881111

23. Boyd JH. Screening for Depression in a Community Sample. *Arch Gen Psychiatry*. 1982;39(10):1195. doi:10.1001/archpsyc.1982.04290100059010

24. Eller LS, Mahat G. Psychological factors in Nepali former commercial sex workers with HIV. *J Nurs Scholarsh*. 2003;35(1):53-60. doi:10.1111/j.1547-5069.2003.00053.x

25. Ghubash R, Daradkeh TK, Al Naseri KS, Al Bloushi NBA, Al Daheri AM. The performance of the center for epidemiologic study depression scale (CES-D) in an Arab female community. *Int J Soc Psychiatry*. 2000;46(4):241-249. doi:10.1177/002076400004600402

26. Guarnaccia PJ, Angel R, Worobey JL. The factor structure of the CES-D in the Hispanic health and nutrition examination survey: The influences of ethnicity, gender and language. *Soc Sci Med*. 1989;29(1):85-94. doi:10.1016/0277-9536(89)90131-7

27. Iwata N, Buka S. Race/ethnicity and depressive symptoms: a cross-cultural/ethnic comparison among university students in East Asia, North and South America. *Soc Sci Med*. 2002;55(12):2243-2252. doi:10.1016/S0277-9536(02)00003-5

28. Munet-Vilaró F, Folkman S, Gregorich S. Depressive Symptomatology in Three Latino Groups. *West J Nurs Res*. 1999;21(2):209-224. doi:10.1177/01939459922043848

29. Myers J, Weissman M. Use of a self-report symptom scale to detect depression in a community sample. *Am J Psychiatry*. 1980;137(9):1081-1084. doi:10.1176/ajp.137.9.1081

30. Roberts RE, Rhoades HM, Vernon SW. Using the CES-D scale to screen for depression and anxiety: Effects of language and ethnic status. *Psychiatry Res*. 1990;31(1):69-83. doi:10.1016/0165-1781(90)90110-Q

31. Roberts R, Vernon S. The Center for Epidemiologic Studies Depression Scale: its use in a community sample. *Am J Psychiatry*. 1983;140(1):41-46. doi:10.1176/ajp.140.1.41

32. Simoni JM, Ng MT. Trauma, coping, and depression among women with HIV/AIDS in New York City. *AIDS Care - Psychol Socio-Medical Asp AIDS/HIV*. 2000;12(5):567-580. doi:10.1080/095401200750003752

33. Illumina. Infinium® HumanMethylation450 BeadChip. 2012:4. https://www.illumina.com/content/dam/illumina-marketing/documents/products/datasheets/datasheet_humanmethylation450.pdf.

34. Illumina. Infinium HD Assay Methylation Protocol Guide. 2015;(November). www.illumina.com.

35. Bibikova M, Barnes B, Tsan C, et al. Genomics High density DNA methylation array with single CpG site resolution Unmethylated locus Methylated locus Unmethylated locus Methylated locus. *Genomics*. 2011;98(4):288-295. doi:10.1016/j.ygeno.2011.07.007

36. Fortin J, Labbe A, Lemire M, et al. Functional normalization of 450k methylation array data improves replication in large cancer studies. *Genome Biol*. 2014;15(503):1-17.

37. Min JL, Hemani G, Davey Smith G, Relton C, Suderman M. Meffil: efficient normalization and analysis of very large DNA methylation datasets. *Bioinformatics*. 2018;34(23):3983-3989. doi:10.1093/bioinformatics/bty476

38. Johnson WE, Li C, Rabinovic A. Adjusting batch effects in microarray expression data using empirical Bayes methods. *Biostatistics*. 2007;8(1):118-127. doi:10.1093/biostatistics/kxj037

39. Leek JT, Storey JD. Capturing Heterogeneity in Gene Expression Studies by Surrogate Variable Analysis. *PLoS Genet*. 2007;3(9):e161. doi:10.1371/journal.pgen.0030161

40. Leek JT, Storey JD. A general framework for multiple testing dependence. *PNAS*. 2008;105(48):18718-18723.

41. Waite LL, Weaver B, Day K, et al. Estimation of Cell-Type Composition Including T and B Cell Subtypes for Whole Blood Methylation Microarray Data. *Front Genet*. 2016;7(February):1-10. doi:10.3389/fgene.2016.00023

42. Houseman EA, Accomando WP, Koestler DC, et al. DNA methylation arrays as surrogate measures of cell mixture distribution. *BMC Bioinformatics*. 2012;13(1). doi:10.1186/1471-2105-13-86

43. Suderman M, Staley JR, French R, Arathimos R, Simpkin A, Tilling K. Dmrff: Identifying Differentially Methylated Regions Efficiently With Power and Control. *bioRxiv*. 2018:508556. doi:10.1101/508556

44. Yin Y, Morgunova E, Jolma A, et al. Impact of cytosine methylation on DNA binding specificities of human transcription factors. *Science (80- )*. 2017;2239(May). doi:10.1126/science.aaj2239

45. Affinito O, Palumbo D, Fierro A, et al. Genomics Nucleotide distance in fl uences co-methylation between nearby CpG sites. *Genomics*. 2020;112:144-150. doi:10.1016/j.ygeno.2019.05.007

46. Saito D, Suyama M. Linkage disequilibrium analysis of allelic heterogeneity in DNA methylation Linkage disequilibrium analysis of allelic heterogeneity in DNA methylation. *Epigenetics*. 2015;10(12):1093-1098. doi:10.1080/15592294.2015.1115176

47. Claus R, Wilop S, Hielscher T, et al. A systematic comparison of quantitative high-resolution DNA methylation analysis and methylation-specific PCR. *Epigenetics*. 2012;7(7):772-780. doi:10.4161/epi.20299

48. Ehrich M, Nelson MR, Stanssens P, et al. Quantitative high-throughput analysis of DNA methylation patterns by base-specific cleavage and mass spectrometry. *Proc Natl Acad Sci U S A*. 2005;102(44):15785-15790. doi:10.1073/pnas.0507816102

49. Staley J, Battram T, Hemani G, Suderman M, Relton C, Guant T. The MRC-IEU catalog of epigenome-wide association studies. EWAS Catalog. http://ewascatalog.org/. Published 2017.

50. Li M, Zou D, Li Z, et al. EWAS Atlas : a curated knowledgebase of epigenome-wide association studies. *Nucleic Acids Res*. 2019;47(October 2018):983-988. doi:10.1093/nar/gky1027

51. Hannon E, Lunnon K, Schalkwyk L, Mill J. Interindividual methylomic variation across blood, cortex, and cerebellum: Implications for epigenetic studies of neurological and neuropsychiatric phenotypes. *Epigenetics*. 2015;10(11):1024-1032. doi:10.1080/15592294.2015.1100786

52. Ressler K, Mercer K, Bradley B, et al. Post-traumatic stress disorder is associated with PACAP and PAC1 receptor. *Nature*. 2011;470(7335):492-497. doi:10.1097/CCM.0b013e31823da96d.Hydrogen

53. Miller MW, Maniates H, Wolf EJ, et al. Brain , Behavior , and Immunity CRP polymorphisms and DNA methylation of the AIM2 gene influence associations between trauma exposure , PTSD , and C-reactive protein. *Brain Behav Immun*. 2018;67:194-202. doi:10.1016/j.bbi.2017.08.022

54. Moser DA, Paoloni-Giacobino A, Stenz L, et al. BDNF methylation and maternal brain activity in a violence-related sample. *PLoS One*. 2015;10(12):e0143427. doi:10.1371/journal.pone.0143427

55. Kim TY, Kim SJ, Chung HG, Choi JH, Kim SH, Kang JI. Epigenetic alterations of the BDNF gene in combat-related post-traumatic stress disorder. *Acta Psychiatr Scand*. 2017;135(2):170-179. doi:10.1111/acps.12675

56. Norrholm SD, Jovanovic T, Smith AK, et al. Differential genetic and epigenetic regulation of catechol-O-methyl-transferase (COMT) is associated with impaired fear inhibition in posttraumatic stress disorder. *Front Behav Neurosci*. 2013;7(MAR):1-10. doi:10.3389/fnbeh.2013.00030

57. Bishop JR, Lee AM, Mills LJ, et al. Methylation of FKBP5 and SLC6A4 in Relation to Treatment Response to Mindfulness Based Stress Reduction for Posttraumatic Stress Disorder. *Front Psychiatry*. 2018;9(September):1-11. doi:10.3389/fpsyt.2018.00418

58. Yehuda R, Daskalakis NP, Bierer LM, et al. Archival Report Holocaust Exposure Induced Intergenerational Effects on FKBP5 Methylation. *Biol Psychiatry*. 2016;80(5):372-380. doi:10.1016/j.biopsych.2015.08.005

59. Kang J, Kim T, Choi J, So H, Kim S. Allele-specific DNA methylation level of FKBP5 is associated with post- traumatic stress disorder. *Psychoneuroendocrinology*. 2019;103(November 2018):1-7. doi:10.1016/j.psyneuen.2018.12.226

60. Schechter DS, Moser DA, Pointet VC, et al. The association of serotonin receptor 3A methylation with maternal violence exposure, neural activity, and child aggression. *Behav Brain Res*. 2017;325:268-277. doi:10.1016/j.bbr.2016.10.009

61. Bam M, Yang X, Zhou J, Ginsberg JP, Leyden Q. Evidence for Epigenetic Regulation of Pro-Inflammatory Cytokines , Interleukin-12 and Interferon Gamma , in Peripheral Blood Mononuclear Cells from PTSD Patients. *J Neuroimmune Pharmacol*. 2016;11:168-181. doi:10.1007/s11481-015-9643-8

62. Uddin M, Galea S, Chang S, Aiello AE, Wildman DE. Gene expression and methylation signatures of MAN2C1 are associated with PTSD. *Dis Markers*. 2011;30:111-121. doi:10.3233/DMA-2011-0750

63. Ziegler C, Wolf C, Schiele MA, et al. Monoamine Oxidase A Gene Methylation and Its Role in Posttraumatic Stress Disorder: First Evidence from the South Eastern Europe (SEE)-PTSD Study. *Int J Neuropsychopharmacol*. 2018;21(5):423-432. doi:10.1093/ijnp/pyx111

64. Labonté B, Azoulay N, Yerko V, Turecki G, Brunet A. Epigenetic modulation of glucocorticoid receptors in posttraumatic stress disorder. 2014;(November 2013). doi:10.1038/tp.2014.3

65. Schechter DS, Moser DA, Paoloni-Giacobino A, et al. Methylation of NR3C1 is related to maternal PTSD, parenting stress and maternal medial prefrontal cortical activity in response to child separation among mothers with histories of violence exposure. *Front Psychol*. 2015;6(May):1-12. doi:10.3389/fpsyg.2015.00690

66. Schür RR, Boks MP, Rutten BPF, et al. Longitudinal changes in glucocorticoid receptor exon 1F methylation and psychopathology after military deployment. *Transl Psychiatry*. 2017;7:e1181. doi:10.1038/tp.2017.150

67. Vukojevic V, Kolassa I, Fastenrath M, et al. Epigenetic Modification of the Glucocorticoid Receptor Gene Is Linked to Traumatic Memory and Post-Traumatic Stress Disorder Risk in Genocide Survivors. *J Neurosci*. 2014;34(31):10274-10284. doi:10.1523/JNEUROSCI.1526-14.2014

68. Yehuda R, Nikolaos P, Koch E, et al. Epigenetic biomarkers as predictors and correlates of symptom improvement following psychotherapy in combat veterans with PTSD. *Front Psychiatry*. 2013;4(September):1-14. doi:10.3389/fpsyt.2013.00118

69. Mcnerney MW, Sheng T, Nechvatal JM, et al. Integration of neural and epigenetic contributions to posttraumatic stress symptoms : The role of hippocampal volume and glucocorticoid receptor gene methylation. *PLoS One*. 2018;13(2):1-14.

70. Yehuda R, Flory JD, Bierer LM, et al. Lower Methylation of Glucocorticoid Receptor Gene Promoter 1F in Peripheral Blood of Veterans with Posttraumatic Stress Disorder. *Biol Psychiatry*. 2015;77(4):356-364. doi:10.1016/j.biopsych.2014.02.006

71. Nawijn L, Krzyzewska IM, van Zuiden M, et al. Oxytocin receptor gene methylation in male and female PTSD patients and trauma-exposed controls. *Eur Neuropsychopharmacol*. 2019;29(1):147-155. doi:10.1016/j.euroneuro.2018.10.006

72. Sadeh N, Spielberg J, Logue M, et al. SKA2 Methylation is associated with decreased prefrontal cortical thickness and greater PTSD severity among trauma-exposed veterans. *Mol Psychiatry*. 2016;21(3):357-363. doi:10.1038/mp.2015.134.SKA2

73. Boks MP, Rutten BPF, Geuze E, et al. SKA2 Methylation is Involved in Cortisol Stress Reactivity and Predicts the Development of Post-Traumatic Stress Disorder ( PTSD ) After Military Deployment. *Neuropsychopharmacoly*. 2016;41:1350-1356. doi:10.1038/npp.2015.286

74. Chang SC, Koenen KC, Galea S, et al. Molecular variation at the SLC6A3 locus predicts lifetime risk of PTSD in the Detroit Neighborhood Health Study. *PLoS One*. 2012;7(6):1-6. doi:10.1371/journal.pone.0039184

75. Koenen K, Uddin M, Chang S, et al. SLC6A4 methylation modifies the effect of the number of traumatic events on risk for posttraumatic stress disorder. *Depress Anxiety*. 2011;28:639-647. doi:10.1002/da.20825

76. Smith A, Ratanatharathorn A, Maihofer A, et al. Epigenome-wide meta-analysis of PTSD across 10 military and civilian cohorts identifies novel methylation loci. *bioRxiv Prepr*. 2019. doi:https://doi.org/10.1101/585109

77. Smith A, Conneely K, Kilaru V, et al. Differential immune system DNA methylation and cytokine regulation in Post-Traumatic Stress Disorder. *Am J Med Genet Paert B Neuropsychiatr Genet*. 2011;156B(6):700-708. doi:10.1109/TMI.2012.2196707.Separate

78. Mehta D, Bruenig D, Carrillo-Roa T, et al. Genomewide DNA methylation analysis in combat veterans reveals a novel locus for PTSD. *Acta Psychiatr Scand*. 2017;136(5):493-505. doi:10.1111/acps.12778

79. Snijders C, Maihofer AX, Ratanatharathorn A, et al. Longitudinal epigenome-wide association studies of three male military cohorts reveal multiple CpG sites associated with post-traumatic stress disorder. *Clin Epigenetics*. 2020;12(1):1-13. doi:10.1186/s13148-019-0798-7

80. Rutten BPF, Vermetten E, Vinkers CH, et al. Longitudinal analyses of the DNA methylome in deployed military servicemen identify susceptibility loci for post-traumatic stress disorder. *Mol Psychiatry*. 2018;23(5):1145-1156. doi:10.1038/mp.2017.120

81. Logue MW, Miller MW, Wolf EJ, et al. An epigenome-wide association study of posttraumatic stress disorder in US veterans implicates several new DNA methylation loci. *Clin Epigenetics*. 2020;12(1):1-14. doi:10.1186/s13148-020-0820-0

82. Maddox SA, Kilaru V, Shin J, et al. Estrogen-dependent association of HDAC4 with fear in female mice and women with PTSD. *Mol Psychiatry*. 2018;23(3):658-665. doi:10.1038/mp.2016.250

83. Uddin M, Ratanatharathorn A, Armstrong D, Kuan P, Allison E. Epigenetic meta-analysis across three civilian cohorts identifies NRG1 and HGS as blood-based biomarkers for post-traumatic stress disorder. *Epigenomics*. 2018;10(12):1585-1601.
